# Drought characterization over Indian sub-continent using GRACE-based indices

**DOI:** 10.1038/s41598-022-18511-2

**Published:** 2022-09-14

**Authors:** Shivam Rawat, Abinesh Ganapathy, Ankit Agarwal

**Affiliations:** Department of Hydrology, Indian Institute of Technology, Roorkee, 247667 India

**Keywords:** Mathematics and computing, Environmental social sciences

## Abstract

Drought is a natural disaster affects water resources, agriculture, and social and economic development due to its long-term and frequent occurrence. It is crucial to characterize and monitor drought and its propagation to minimize the impact. However, spatiotemporal assessment of drought characteristics over India at the sub-basin scale based on terrestrial water storage is unexplored. In this study, the Terrestrial water storage anomalies (TWSA) obtained from a Gravity Recovery and Climate Experiment and precipitation data are used to characterize the propagation of drought. Combined Climatological Deviation Index (CCDI) and GRACE-Drought Severity Index (GRACE-DSI) were computed as CCDI utilizes both precipitation and TWSA data while GRACE-DSI uses only TWSA data. Our results showed that GRACE-DSI exhibits significant negative trends over most of the Indian sub-basins compared to CCDI, indicating that most of the drought events are due to depletion of TWS. While other sub-basins show changing trends for GRACE-DSI and CCDI. The number of sub-basins showing significant negative trends for GRACE-DSI is more than that for CCDI. Hence TWS is depleting for most of the subbasins in India. Our results show that Indo-Gangetic plains face many drought events during 2002–2004, 2009–2014 & 2015–2017. Maximum drought duration and drought severity obtained for the area of North Ladakh (not draining into Indus basins) by GRACE-DSI are 26 months (2002–2004) and − 44.2835, respectively. The maximum drought duration and drought severity obtained for the Shyok sub-basin by CCDI is 17 months (2013–2015) and − 13.4392, respectively. Monthly trend analysis revealed that 39 & 23 no. of sub-basins show significant negative GRACE-DSI trends for October and CCDI for November, respectively. At the same time, the seasonal trend shows that total 34 and 14 sub-basins exhibited a significant negative trend at post-monsoon Kharif season for both the GRACE-DSI & CCDI, respectively.

## Introduction

Drought is a severe weather condition characterized by a lack of moisture. It is broadly characterized by meteorological, hydrological, agricultural, and human-induced drought. Drought in India occurs in regions with high as well as sparse rainfall. About 68% of the country is prone to drought at various levels. Thirty five percent of the regions receive rainfall between 750 and 1125 mm are considered drought-prone, and 33% receive less than 750 mm are subject to chronically drought-prone. Water deficiency conditions in the Himalayan region are also not unusual. In the past, India has faced major droughts that have had a long-lasting impact on water resources, agriculture, economy, and social livelihood^1^. In India, some of the historic droughts caused famines that resulted in the death of millions of people^2^. A drought of 2015 affected crop production and water availability in the Indo-Gangetic plain and Maharashtra region^3^. The 2015–2016 drought resulted in significant groundwater depletion in India's Indo-Gangetic plains and southern states, and about 330 million people in ten states were affected by this drought^4^. Deficiency in terrestrial water storage was particularly severe during the droughts of 2004, 2009, and 2012, with the peak in the Ganga basin in 2009^5^. Therefore, it is essential to monitor, assess and quantify drought so that proper mitigation strategies can be evaluated and implemented successfully. Jain et al.^6^ work on the assessment of vulnerability to drought using the Integrated Drought Vulnerability Index.

Due to the complex nature of drought and dependency on several hydrological parameters, a large variety of drought monitoring indices have been developed. Most of the drought indices like Palmer Drought Severity Index (PDSI)^7^, Standardized precipitation index(SPI)^8^, Standardized Precipitation Evapotranspiration Index (SPEI)^9^, Standardized Runoff Index^10^, Standardized Drought Index^11^, are based on model simulation or observation of land surface states and fluxes while Normalized Difference Vegetation Index^12^, Vegetation Drought Response Index^13^ based on remote sensing techniques but are restricted to show agricultural drought only. PDSI computation is based on the climate of the Mid-Western United States, and it shows limitations for other climatic conditions. SPI and SPEI show multi-time scale features, which create difficulty in choosing an appropriate timescale for drought characterization. McKee et al.^8^ developed the Standardized Precipitation Index for drought characterization based on Precipitation data. SPI has multi-time scale features, making it suitable for drought characterization at different time scales. Vicente-Serrano et al.^9^ developed a new climatic drought index: The standardized Precipitation Evapotranspiration Index (SPEI). SPEI utilizes both Precipitation and Temperature data to characterize drought accurately. SPEI is mathematically similar to SPI and represents drought characteristics better than SPI because of the inclusion of Temperature data. Jain et al.^14^ compare various drought indices like SPI, Effective Drought Index, China Z-Index, Rainfall Departure, and Rainfall Decile based Drought Index and suggest that EDI better correlates with other indices for all time scales.

Presently, after the evolution of Gravity Recovery and Climate Experiment (GRACE), it is now possible that direct observation of Terrestrial water storage (TWS) from GRACE or its deviation from climatologic mean can be used as a metric for drought characterization^15,16^. Some of GRACE drought indices that are used widely for drought characterization nowadays include GRACE-Drought Severity Index (GRACE-DSI)^17^, Water Storage Deficit Index (WSDI)^18^, Combined Climatologic Deviation Index (CCDI)^19^, GRACE-Based Groundwater Drought Index (GGDI)^20^.

Combined Climatologic Deviation Index (CCDI) utilizes both Precipitation and GRACE-TWSA data while GRACE_DSI uses only GRACE-TWSA data^17,19,21^. CCDI includes all aspects of drought occurrences like Meteorological, Agricultural, Hydrological and Anthropogenic activities. Kumar et al.^20^ developed the GRACE-Based Groundwater Drought Index to monitor groundwater storage with the help of GRACE-TWSA, Soil Moisture Storage Anomaly, and canopy water storage anomaly.

Although extensive studies have already been conducted on the drought analysis, temporal changes and the characterization of drought at the sub-basin scale are limited. This study focuses on the sub-basin spatial scale to examine the spatio-temporal changes in the GRACE-DSI and CCDI and characterize drought using the drought indices.

The paper is organized as follows: “Study area and data used” covers the description of the study area and datasets used in this analysis, “Methodology” discusses the methodology and methods utilized in the current study, followed by “Results”, where the obtained results are presented, and finally, “Discussion” and “Conclusion” covers the discussion and conclusions respectively.

## Study area and data used

### Study area

**Figure 1:**
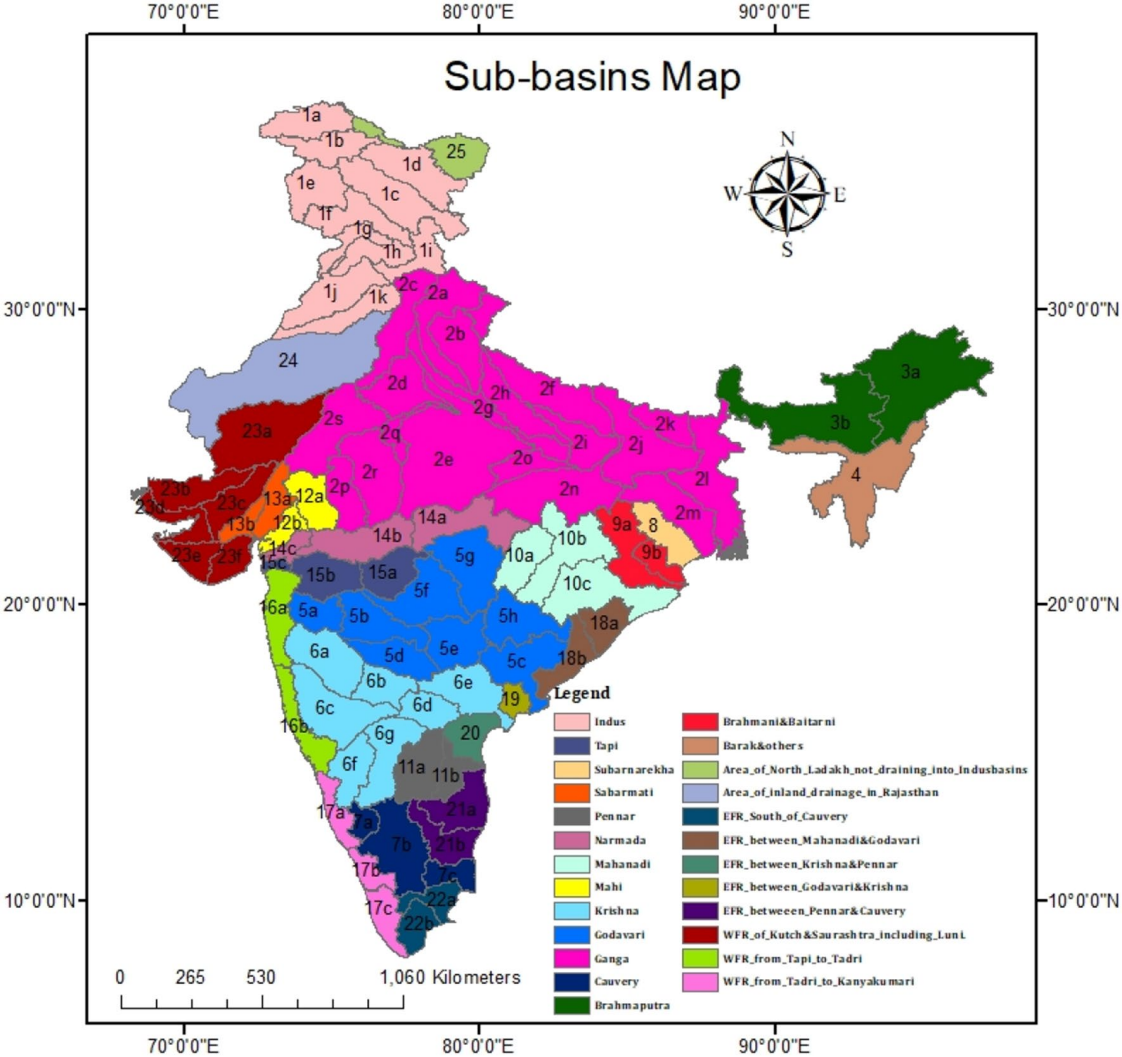
Study area map shows Indian river sub-basins. For details of notation and name of sub-basins, refer to Table S1 in the “Supplementary Section”.

This study analyses drought characteristics for entire India at sub-basin scales. The study area comprises major river basins, including Ganga, Indus, Brahmaputra, Godavari, Krishna, Cauvery, Mahanadi, Sabarmati, Narmada, Tapi, etc. We consider 90 sub-basins within the 25 river basins lying inside the Indian boundary for this study. Catchment area details of basins/sub-basins are provided in Table S1 “Supplementary material”. We have used the Indian sub-basin boundaries following the Central Water Commission (CWC) and India Water Resources Information System (India-WRIS) delineation to analyze the drought characteristics^22^ (Fig. 1). Refer to Table S1 (“Supplementary section”) for the details of notation and naming of basins/sub-basins.

### Data

We only utilized observed data in the analysis to avoid the uncertainties imposed by the modelled data. Further, we have not considered uncertainties caused by the interpolation, of the pointed data, to obtain gridded data.

#### Precipitation

For this study, we have used Indian Meteorological Department (IMD) gridded precipitation data having a spatial resolution of 0.25° × 0.25° (https://imdpune.gov.in/Clim_Pred_LRF_New/Grided_Data_Download.html) for the duration of April 2002 to June 2017 (183 months) for the entire study area^23^. Totally 6955 rain gauge stations all over India is used to produce the IMD gridded data and inverse weighted distance interpolation technique is used to convert the gauge data into gridded data^24^. Further, rainfall records from varying number of rain gauges are used to develop the dataset with increasing gauge density over the time^25^. Although there is a varying density of rain gauges, multiple studies considered IMD precipitation as the reference data to compare global precipitation datasets^26,27^.

#### GRACE-Terrestrial Water Storage Anomaly (GRACE-TWSA)

We have considered GRACE monthly mass grids (RL06 mascon solutions) having a spatial resolution of 0.5° × 0.5° processed at Jet Propulsion Laboratory (JPL) for this study. GRACE dataset provides gridded monthly global water storage/height anomalies relative to time-mean (time baseline from Jan 2004 to Dec 2009)^28^. The Coastal Resolution Improvement (CRI) filter reduces signal leakage errors across coastlines^29^. We have considered data from April 2002 to June 2017 due to a gap in the data for 11 consecutive months after June 2017. We apply the linear interpolation technique for missing data between April 2002 and June 2017^30^ (https://grace.jpl.nasa.gov/data/get-data/jpl_global_mascons/).

## Methodology

In this study, we analyzed the GRACE Mascon and IMD precipitation data (Refer to “Data” for the data characteristics). Once the data is collected, we have performed monthly and seasonal trend analysis for both TWSA and IMD precipitation data at subbasin scales for the entire country to evaluate the trends in data. Regions exhibiting significant trends are identified using the Mann–Kendall test, and the magnitude of change is determined using the Theil–Sen approach (TSA). After that, we computed GRACE-DSI and CCDI index for drought characterization. We have also performed monthly and seasonal trends analysis for GRACE-DSI and CCDI index to analyze drought trends. Further we identified the drought events for both CCDI & GRACE-DSI based on the threshold value of − 0.5 (Table 1). Drought events are defined only if the drought indices value are less than − 0.5 continuously for greater than or equal to three months.

**Table 1:** Range of GRACE-DSI and CCDI for drought characterization based on the study by Satish Kumar et al.^21^.

Drought category	GRACE-DSI/CCDI
W4: exceptionally wet	Two or greater
W3: extremely wet	1.6–1.99
W2: severely wet	1.3–1.59
W1: moderately wet	0.8–1.29
W0: abnormally wet	0.5–0.79
N: near normal	0.49 to − 0.49
D0: abnormally dry	− 0.5 to − 0.79
D1: moderately dry	− 0.8 to − 1.29
D2: severely dry	− 1.3 to − 1.59
D3: extremely dry	− 1.6 to − 1.99
D4: exceptionally dry	− 2 or less

A negative value of the drought index represents the dry condition, while a positive value of the drought index represents the wet condition.

**Figure 2:**
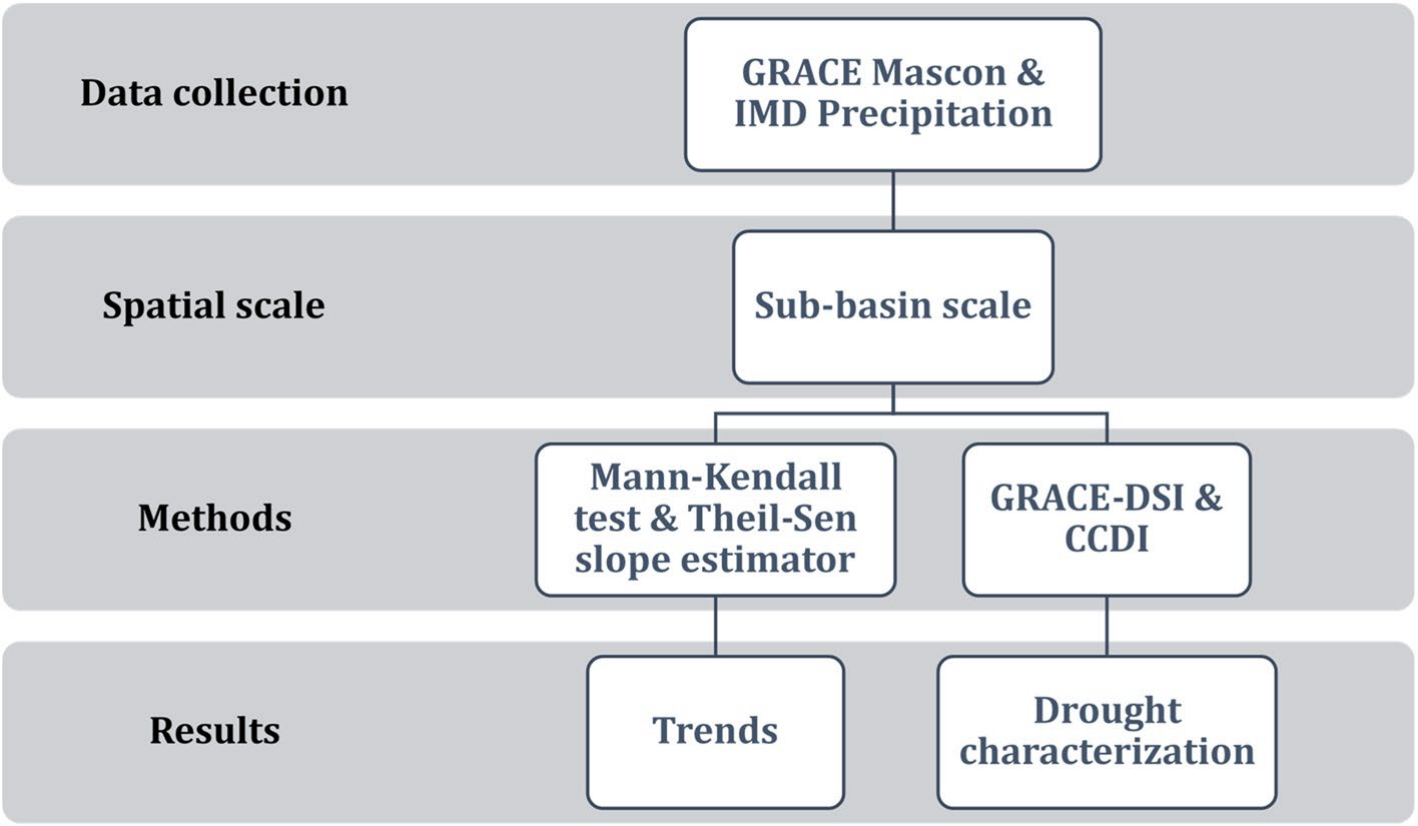
Flow chart of the proposed study discussed above with the spatial scale involved and the methods to evaluate the trend and drought characterization.

Seasonal analysis for Post Monsoon Rabi (January to March), Pre Monsoon (April to June), Monsoon (July to September), and Post Monsoon Kharif (October to December) are done. We have considered only those drought events for which the drought index is less than − 0.5 continuously for greater than equal to three months for both the indices. We have performed this to evaluate whether drought is due to a deficit of TWSA or precipitation deficit or due to a deficit of both Precipitation and TWSA. In this study, the flow chart of the adopted methodology is shown in Fig. 2.

### GRACE-DSI computation

GRACE DSI utilizes only TWS data and evaluates drought events based on the deficit in TWS with respect to its mean. GRACE-DSI can be computed by standardizing TWSA (as in Eq. (1))^17^.

1 $${GRACE-DSI}_{i,j}=\frac{{TWSA}_{i,j}-{\overline{TWSA} }_{j}}{{TWSA}_{\sigma ,j}}$$

where ${TWSA}_{i,j}$ represents TWSA of the year i (i = 2002 to 2017) and month j (j = 1 to 12), ${\overline{TWSA} }_{j}$ is monthly mean TWSA and ${TWSA}_{\sigma ,j}$ is standard deviation of TWSA for month j.

### CCDI computation

CCDI utilizes both Precipitation and GRACE-TWSA data. It can identify the drought caused due to either deficit in Precipitation or TWS or due to partial contribution of both. For CCDI computation, we first found the Precipitation Anomaly (PA), the deviation of monthly precipitation from their mean monthly precipitation. Deviation of monthly PA from their mean monthly PA, we get Precipitation Anomaly Residual (PAR). Similarly, we have to find the Terrestrial Water Storage Anomaly Residual (TWSAR) by taking the deviation of monthly TWSA from their mean monthly TWSA. After computation of TWSAR and PAR, we have to find Combined Deviation (CD), the summation of TWSAR and PAR. By standardization of CD, we get CCDI.

2 $${PA}_{i,j}={P}_{i,j}-{P}_{\mu }$$

3 $${PAR}_{i,j}={PA}_{i,j}-{\overline{PA} }_{j}$$

4 $${TWSAR}_{i,j}={TWSA}_{i,j}-{\overline{TWSA} }_{j}$$

5 $${CD}_{i,j}={TWSAR}_{i,j}+{PAR}_{i,j}$$

6 $${CCDI}_{i,j}=\frac{{CD}_{i,j}-\overline{CD}}{{CD }_{\sigma }}$$

where ${P}_{i,j}$ represents the amount of precipitation in year i (i = 2002 to 2017) and month j (j = 1 to 12), ${P}_{\mu }$ the monthly average precipitation, and ${PA}_{i,j}$ is PA in the year i and month j, ${TWSA}_{i,j}$ represents TWSA of the year i and month j, ${\overline{TWSA} }_{j}$ is monthly mean TWSA, ${PAR}_{i,j}$ & ${TWSAR}_{i,j}$ respectively represents PAR and TWSAR for the year i and month j, ${CD}_{i,j}$ is the combined deviation for the year i and month j, $\overline{CD }$ is monthly mean CD and ${CD}_{\sigma }$ is standard deviation CD for month j.

### Mann–Kendall Trend Analysis (MK Test)

Mann Kendall Test is a non-parametric test used to determine the presence or absence in a given time series data^31,32^. The null hypothesis, H_0_, states there is no monotonic trend, and this is tested against one of three possible alternatives hypothesis, H_α_: (1) there is a monotonic upward trend, (2) there is a monotonic downward trend, or (3) there is either a monotonic upward or a downward trend. MK test statistics can be defined as

7 $$S=\sum_{i=1}^{n-1}\sum_{j=i+1}^{n}sgn({X}_{j}-{X}_{i})$$

where n denotes the length of the dataset, ${X}_{i}$ and ${X}_{j}$ Represents data points in time series i and j, respectively (i < j).

8 $$sgn\left(Xj-Xi\right)=\left\{\begin{array}{c}-1,if\, {X}_{j}-{X}_{i}<0\\ 0,if\, {X}_{j}-{X}_{i}=0\\ +1, if\, {X}_{j}-{X}_{i}>0\end{array}\right\}$$

It has been documented that for n ≥ 10, statistics S is normally distributed with

9 $$\mathrm{E}\left(\mathrm{S}\right)=0$$

10 $$V\left(S\right)=\frac{n\left(n-1\right)\left(2n+5\right)-\sum_{i=1}^{m}{t}_{i }({t}_{i }-1)(2{t}_{i }+5)}{18}$$

where E(S) is the mean, V(S) is the variance of S, m is the number of tied groups, and t_i_ is the size of the i-th tied group. The standard normal test statistics Z is given by

11 $$Z=\left\{\begin{array}{c}\frac{S+1}{\sqrt{V\left(S\right)}}, if\, S<0\\ 0, if\, S=0\\ \frac{S-1}{\sqrt{V\left(S\right)}}, if\, S>0\end{array}\right\}$$

A positive Z score represents an increasing trend, whereas a negative Z score represents a decreasing trend. We have considered a significance level value of 0.05 for this study.

### Theil-Sen's slope estimator

Theil-Sen's approach is used to estimate the magnitude of change^33,34^. Theil-Sen's slope (β) is defined as

12 $$\beta =median\left(\frac{{X}_{j}-{X}_{i}}{j-i}\right)$$

where ${X}_{i}$ and ${X}_{j}$ Represents data points in time series i and j, respectively (i < j). Thus, a positive value of β represents an upward trend, whereas a negative value of β represents a downward trend.

### Drought duration and drought severity

Drought duration is the period where the drought index value is below the fixed threshold value (for this study, we have taken a threshold value of − 0.5). Drought Severity is the cumulative value of the drought index within the drought duration. The drought duration and drought severity of each event computed for GRACE-DSI and CCDI are listed in Tables S2 and S3 in the “Supplementary Material”.

## Results

After computing the GRACE-DSI and CCDI, drought events obtained from the GRACE-DSI and CCDI are highlighted and presented in “GRACE-DSI identified drought event analysis” and “CCDI identified drought event analysis” alongside the precipitation anomaly of the respective sub-basins. The spatio-temporal trend pattern of the drought indices at the monthly and seasonal windows is shown in “Monthly and seasonal trend analysis”.

### GRACE-DSI identified drought event analysis

**Figure 3:**
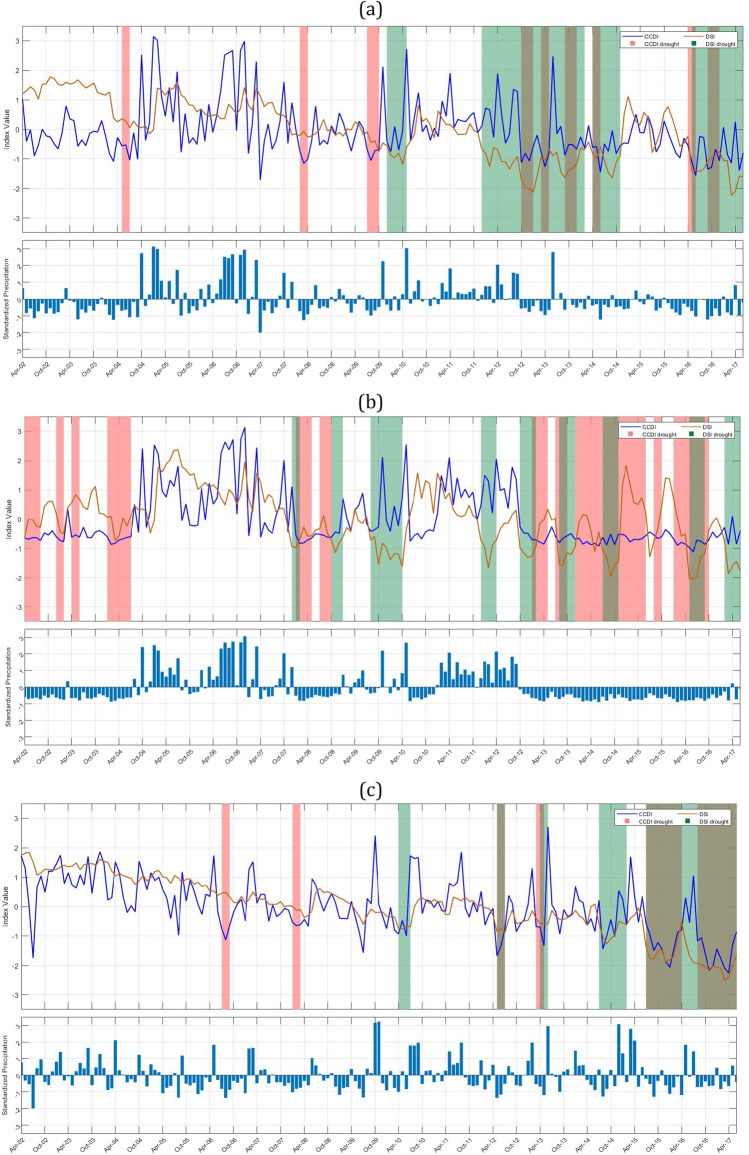

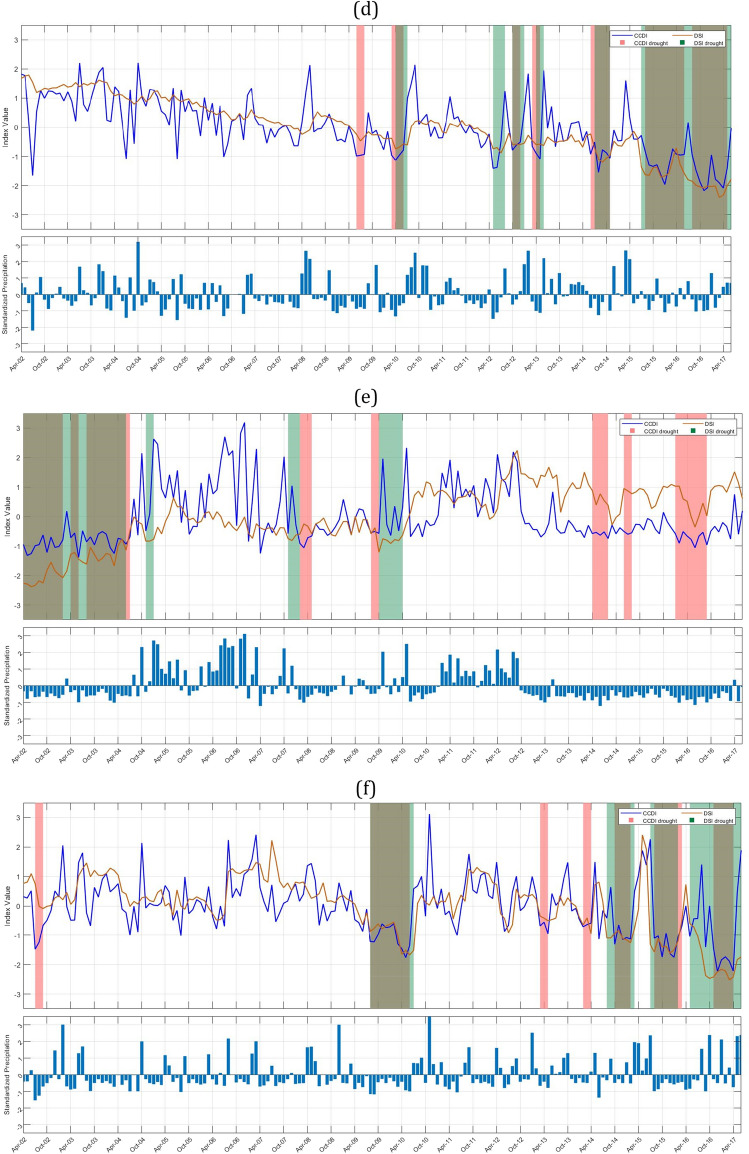

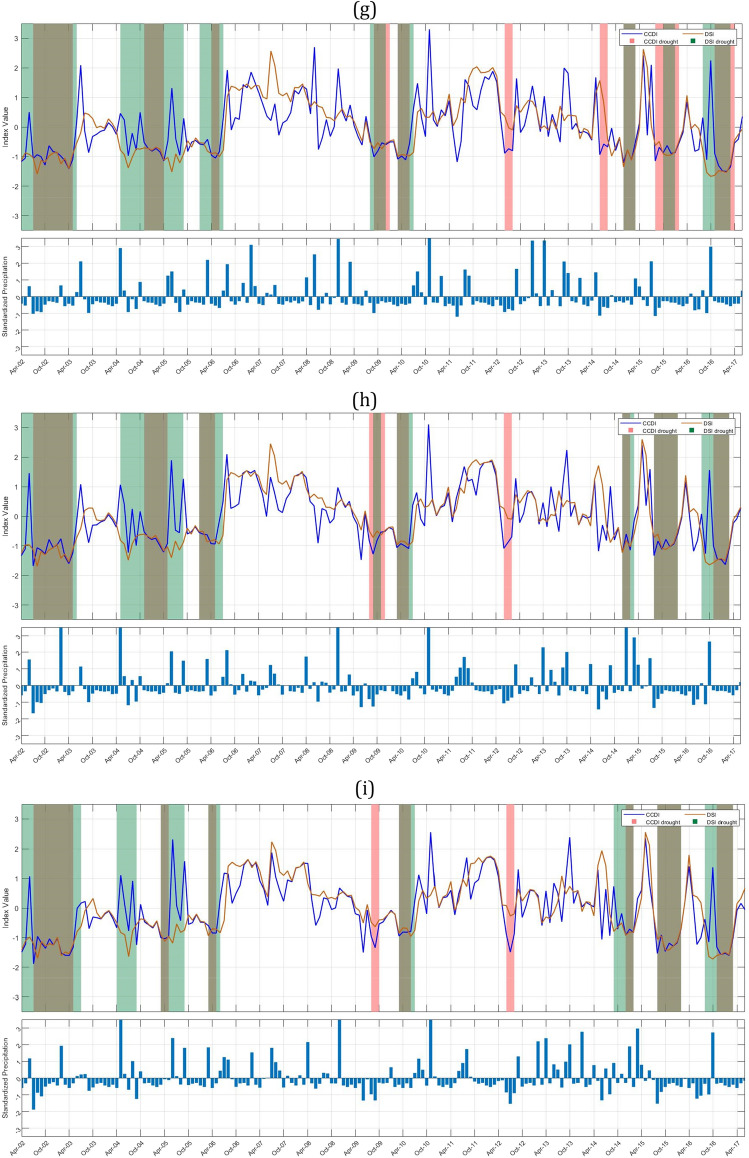

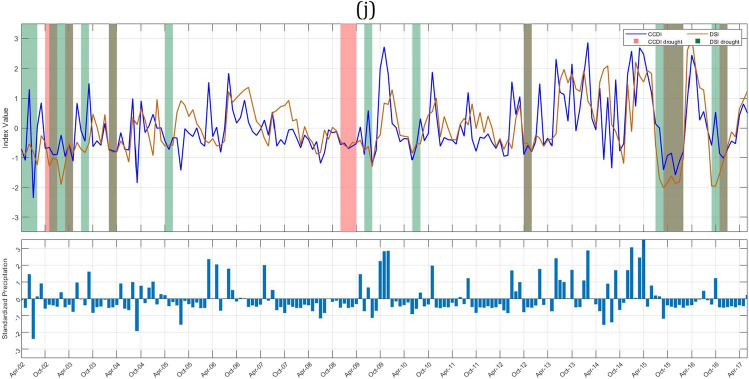
Time series of CCDI (blue line) and GRACE-DSI (orange line) are plotted along with the drought events identified based on CCDI (pink bands) and GRACE-DSI (green bands). The bottom bar graph shows the standardized precipitation variation over time (April 2002–June 2017). This plot is to understand whether the occurrence of drought is due to the deficit in precipitation, TWS, or both. (**a**) Indus Upper sub-basin (**b**) Shyok (**c**) Ramganga (**d**) Yamuna Upper (**e**) Area of North Ladakh not draining into Indus basin (**f**) Luni Upper (**g**) Luni lower (**h**) Saraswati (**i**) Sabarmati lower (**j**) Narmada lower sub-basin.

To study the drought characteristics over Indian subbasins, we have plotted drought events identified by GRACE-DSI and CCDI indices for 90 subbasins as shown in Fig. 3. A dry spell continuing for three or more months is only considered for drought characterization. Any intermediate wet spell during this dry spell continuing three or fewer months is considered a temporary anomaly within this persisting dry streak.

GRACE-DSI utilizes only precipitation data and evaluates drought events based on the deficit in TWS. Our results show that among Indus sub-basins, Indus upper sub-basin (Fig. 3a, 1c) shows a maximum of four drought events DE1 (Dec 2009–May 2010), DE2 (Dec 2011–Feb 2014), DE3 (Apr 2014–Nov 2014), DE4 (May 2016-June 2017), and highest drought duration and drought severity are 26 months (2011–2014) and − 31.5826 respectively. Among these, most of the drought events occur due to a deficit in TWS and only for a short period in between; In Figure 3b the Shyok sub-basin (1d) shows eight drought events: DE1 (Oct 2008–Jan 2009), DE2 (Aug 2009–Apr 2010), DE3 (Dec 2011–Apr 2012), DE4 (Oct 2012–Feb 2013), DE5 (Aug 2013–Dec 2013), DE6 (July 2014–Nov 2014), DE7 (May 2016–Sept 2016), DE8 (Feb 2017–June 2017) and maximum drought duration and drought severity obtained for the Shyok sub-basin are eight months (2009–2010) and − 9.9854 respectively. In Fig. 3b, it is clear that DE1, DE2, DE3, and DE8 occur due to a deficit in TWS, while other events occur due to a deficit in both precipitation and TWS. The Ramganga sub-basin (2b) shows three drought events (Fig. 3c) DE1 (Apr 2010–July 2010), DE2 (July 2014–Feb 2015), DE3 (July 2015–June 2017), and the highest drought duration and drought severities are 23 months (2015–2017) and − 42.4152, respectively. Major drought events in the Ramganga sub-basin are due to the deficit in precipitation and TWS, while some drought events only occur due to a deficit in TWS. The Yamuna upper sub-basin (2c) shows five drought events (Fig. 3d) DE1 (Apr 2010–July 2010), DE2 (May 2012–Aug 2012), DE3 (Oct 2012–Jan 2013), DE4 (July 2014–Nov 2014), DE5 (July 2015–June 2017), and highest drought duration and drought severity are 23 months (2015–2017) and − 41.4251 respectively. The area of North Ladakh not draining into the Indus basin (25) has shown three drought events (Fig. 3e) DE1 (Apr 2002–June 2004), DE2 (Nov 2007–Feb 2008), DE3 (Oct 2009–Apr 2010), and the highest drought duration and drought severity has shown are 26 months (2002–2004) and − 44.2835 respectively. It is interesting to note in this sub-basin that all the GRACE-DSI characterized drought events are observed in the first half of the study period (2002-2010). The Luni upper sub-basin (23a) shows four drought events (Fig. 3f) DE1 (Aug 2009–July 2010), DE2 (Aug 2014–March 2015), DE3 (July 2015–Feb 2016), DE4 (May 2016–June 2017), and the highest drought duration and drought severity shown are 13 months (2016–2017) and − 26.2762, respectively. The Luni lower sub-basin (23b) shows eight drought events (Fig. 3g) DE1 (July 2002–June 2003), DE2 (May 2004–Sept 2005), DE3 (Jan 2006–July 2006), DE4 (Aug 2009–Dec 2009), DE5 (March 2010–July 2010), DE6 (Dec 2014–March 2015), DE7 (Oct 2015–Jan 2016), DE8 (Aug 2016–March 2017) and highest drought duration and drought severity shown are 16 months (2004–2005) and − 15.9402 respectively. The Saraswati sub-basin (23c) shows seven drought events (Fig. 3h) DE1 (Apr 2002–June 2003), DE2 (May 2004–Sept 2005), DE3 (Jan 2006–July 2006), DE4 (March 2010–July 2010), DE5 (Dec 2014–March 2015), DE6 (Aug 2015–Feb 2016), DE7 (Aug 2016–March 2017), and highest drought duration and drought severity has shown are 16 months (2004–2005) and − 15.4911 respectively. It can also be noted that although all these sub-basins Luni upper (23a), Luni lower (23b) & Saraswati (23c) are adjacent to each other, they show distinct pattern of drought events. Especially in the Luni upper sub-basin all the drought events are between 2009 and 2017, whereas other two sub-basins have diversed drought events through out the study period. Further, we may also need to examine that whether this is due to the difference in the administrative boundary since major portion of the Luni upper sub-basin lies in Rajasthan state and other two sub-basins in Gujarat. The Sabarmati lower sub-basin (13b) shows eight drought events (Fig. 3i) DE1 (Apr 2002–July 2003), DE2 (Apr 2004–Sept 2004), DE3 (March 2005–Sept 2005), DE4 (March 2006–June 2006), DE5 (March 2010–July 2010), DE6 (Sept 2014–Feb 2015), DE7 (Aug 2015–Feb 2016), DE8 (Aug 2016–March 2017) and highest drought duration and drought severity shown are 15 months (2002–2003) and − 19.5338 respectively. The Narmada lower sub-basin (14c) shows five drought events (Fig. 3j) DE1 (Apr 2002–Aug 2003), DE2 (Nov 2003–Sept 2004), DE3 (March 2005–June 2005), DE4 (Aug 2015–Feb 2016), DE5 (Aug 2016–Feb 2017), and highest drought duration and drought severity shown are 16 months (2002–2003) and − 25.3915 respectively. The Tapi lower sub-basin shows five drought events DE1 (Apr 2002–Aug 2003), DE2 (Jan 2004–Sept 2004), DE3 (March2005–June2005), DE4 (Aug 2015–Feb 2016), DE5 (Aug 2016–Feb 2017), and the highest drought duration and drought severity has shown are 16 months (2002–2003) and − 25.3915 respectively. We have listed all the drought events obtained through GRACE-DSI, drought duration, and drought severity over the major Indian sub-basins (Supplementary Table S3). We also provide the drought events plots for all sub-basins in Supplementary Fig. S3.

### CCDI identified drought event analysis

CCDI utilizes both precipitation and GRACE-TWSA data. It can identify the drought caused due to either deficit in precipitation or TWS or due to partial contribution of both as shown in Fig. 3.

The Indus upper sub-basin (1c) shows a maximum of four drought events (Fig. 3a) DE1 (July 2009–Oct 2009), DE2 (Oct 2012–Jan 2013), DE3 (Sept 2013–Dec 2013), DE4 (Sept 2016–Dec 2016), and highest drought duration and drought severity are three months (2016) and − 4.36056 respectively. Among Indus sub-basins, the Shyok sub-basin (1d) shows seven major drought events: DE1 (Apr 2002–Aug 2002), DE2 (Jan 2004–July 2004), DE3 (Jan 2008–Oct 2008), DE4 (Jan 2013–May 2013), DE5 (July 2013–Oct 2013), DE6 (Dec 2013–June 2015), DE7 (Jan 2016–Oct 2016). The maximum drought duration and severity obtained for the Shyok sub-basin is 17 months (2013–2015) and − 13.4392, respectively. We can notice from Fig. 3b that drought events DE1, DE2, and DE3 occur majorly due to deficit in precipitation, in addition there is no GRACE-DSI characterized drought events during that period. Although Indus upper and Shyok sub-basins are adjacent to each other, maximum drought duration and severity with respect to GRACE-DSI and CCDI have contrasting characteristics. This necessitates the importance of downscaling of the drought characterization to sub-basin level and application of multiple drought indices with difference properties. Brown bands are obtained for drought due to both deficits in precipitation and TWS. The Ramganga sub-basin (2b) shows two drought events (Fig. 3c) DE1 (July 2015–Apr 2016) and DE2(Aug 2016–June 2017), and the highest drought duration and drought severity are 10 months (2016–2017) and − 17.6832, respectively. The Yamuna upper sub-basin (2c) shows four drought events DE1 (March 2010–June 2010), DE2(June 2014–Nov 2014), DE3(Aug 2015–June 2016), DE4(Aug 2016–May 2017), and the highest drought duration and drought severity are 10 months (2015–2016) and − 16.6557 respectively. Further, drought events occurring in Yamuna upper are due to the deficit in precipitations and TWS (Fig. 3d). The area of North Ladakh, not drainage into the Indus basin, (25) shows five drought events (Fig. 3e) DE1(Apr2002–Feb2003), DE2(Aug2003–July2004), DE3(Feb2008–May2008), DE4(Apr2014–Aug2014), DE5(Jan2016–Sept2016) and maximum drought duration and drought severity has shown are 11 months (2003–2004) and − 10.8656 respectively. It is interesting to observe that DE1 and DE2 occur due to a deficit in precipitation and TWS, while other events in the later part of the study period occur only due to a precipitation deficit. The Luni upper sub-basin (23a) shows four drought events (Fig. 3f) DE1(Aug 2009–June 2010), DE2(Oct 2014–Feb 2015), DE3(Aug 2015–March 2016), DE4(Nov 2016–Apr 2017), and the highest drought duration and drought severity are 10 months (2009–2010) and − 11.9381 respectively. The Luni lower sub-basin (23b) shows seven drought events (Fig. 3g) DE1(July 2002–May 2003), DE2(Nov 2004–Apr 2005), DE3(Sept 2009–Jan 2010), DE4(March 2010–June 2010), DE5(Dec 2014–March 2015), DE6(Aug 2015–Feb 2016), DE7(Nov 2016–Apr 2017) and highest drought duration and drought severity are 10 months (2002–2003) and − 10.203 respectively. The Saraswati sub-basin (23c) shows six drought events (Fig. 3h) DE1(July 2002–May 2003), DE2(Nov 2004–May 2005), DE3(Sept 2009–Jan 2010), DE4(March 2010–June 2010), DE5(Aug 2015–Feb 2016), DE6(Nov 2016–March 2017) and highest drought duration and drought severity are 10 months (2002–2003) and − 12.9316 respectively. CCDI characterized drought patterns of these three sub-basins are exhibiting similar characteristics as GRACE-DSI, with the contrasting characteristics between Luni upper and Luni lower & Saraswati sub-basin in the first hallf of the study period (2002-2009). The Sabarmati lower sub-basin (13b) shows three drought events (Fig. 3i) DE1(July 2002–May 2003), DE2(Aug 2015–Feb 2016), DE3(Nov 2016–March 2017), and the highest drought duration and drought severity are 10 months (2002–2003) and − 14.7505, respectively. Finally, the Narmada lower sub-basin (14c) has four CCDI-characterized drought events (Fig. 3j) DE1(Oct 2002–May 2003), DE2(Nov 2003–May 2004), DE3(Aug 2015–Feb 2016), DE4(Nov 2016–Feb 2017), and the highest drought duration and drought severity are 7 months (2002–2003) and − 12.8709, respectively. We have listed the major drought events identified by CCDI, drought duration, and drought severity for all Indian sub-basins (Supplementary Table S2).

### Monthly and seasonal trend analysis

**Figure 4:**
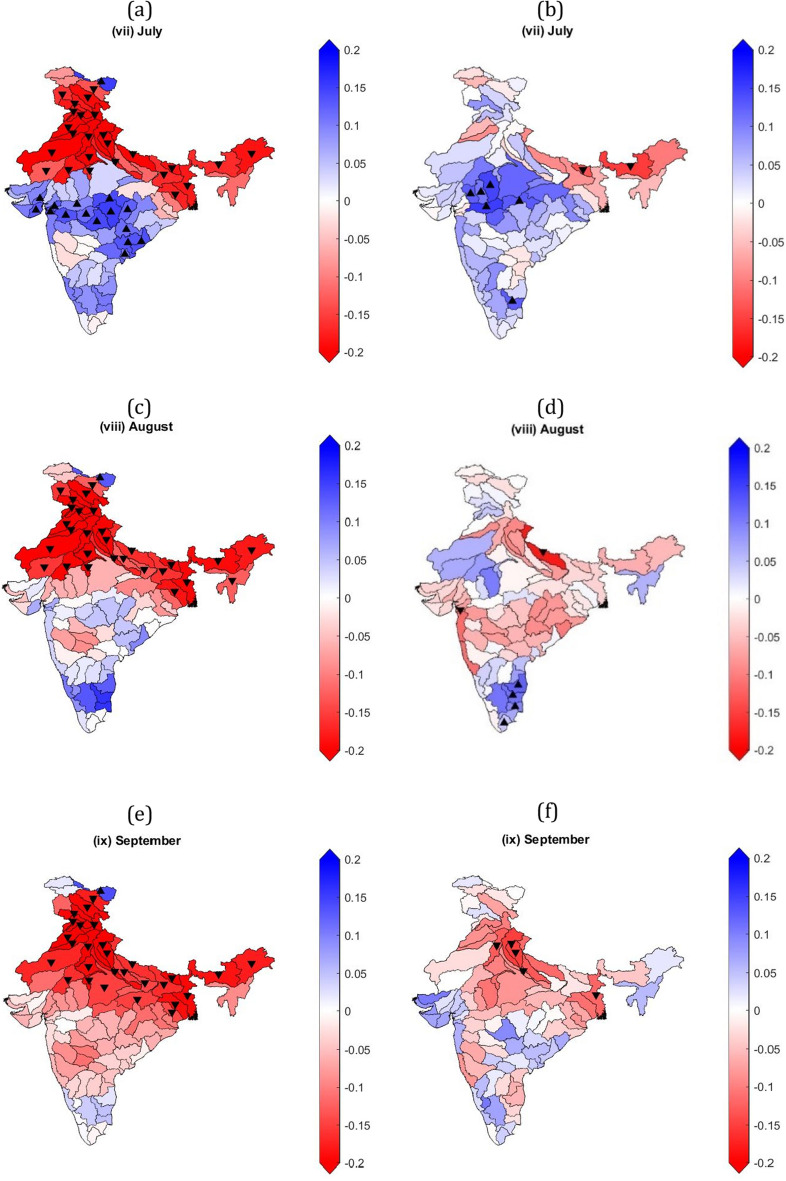
Monthly GRACE-DSI/CCDI trends using the Mann Kendall trend test and Theil-Sen's slope estimator over major Indian river basins for July, August, and September months. (**a**) GRACE-DSI trends for July month, (**b**) CCDI trends for July month, (**c**) GRACE-DSI trends for August month, (**d**) CCDI trends for August month, (**e**) GRACE-DSI trends for September month, (**f**) CCDI trends for September month. Colormap over the basin represents the slope obtained from TSA, △ represents a significant increasing trend, and ▽ indicates a significant decreasing trend based on the Mann–Kendall test.

Monthly trend analysis shows that most of the sub-basins of Ganga and Indus have high significant negative GRACE-DSI trends for July, August, and September months (Fig. 4a,c,e, respectively). Some sub basins of Godavari, Narmada, Sabarmati, and Tapi show significant positive GRACE-DSI trends in July (Fig. 4a). While only a few sub-basins of Ganga and Indus show significant negative CCDI trends for July, August, and September months (Fig. 4b,d,f) respectively). Five sub-basins of Ganga show significant positive CCDI trends in July (Fig. 4b). Other sub-basins show either non-significant positive or negative trends for CCDI and GRACE-DSI. Some of the sub-basins that show significant negative GRACE-DSI trends and significant positive CCDI trends conclude that sub-basin that get sufficient rainfall does not show negative CCDI trends. But for, the sub-basins, which has significant negative trends for both CCDI and GRACE-DSI, depicts that the sub-basin is getting low or no rainfall, due to which TWS is continuously depleting. CCDI monthly trends for most sub-basins are showing changing trends. Monthly and seasonal CCDI and GRACE-DSI trends for all months are provided in Supplementary Figs. S1 and S2, respectively.

**Figure 5:**
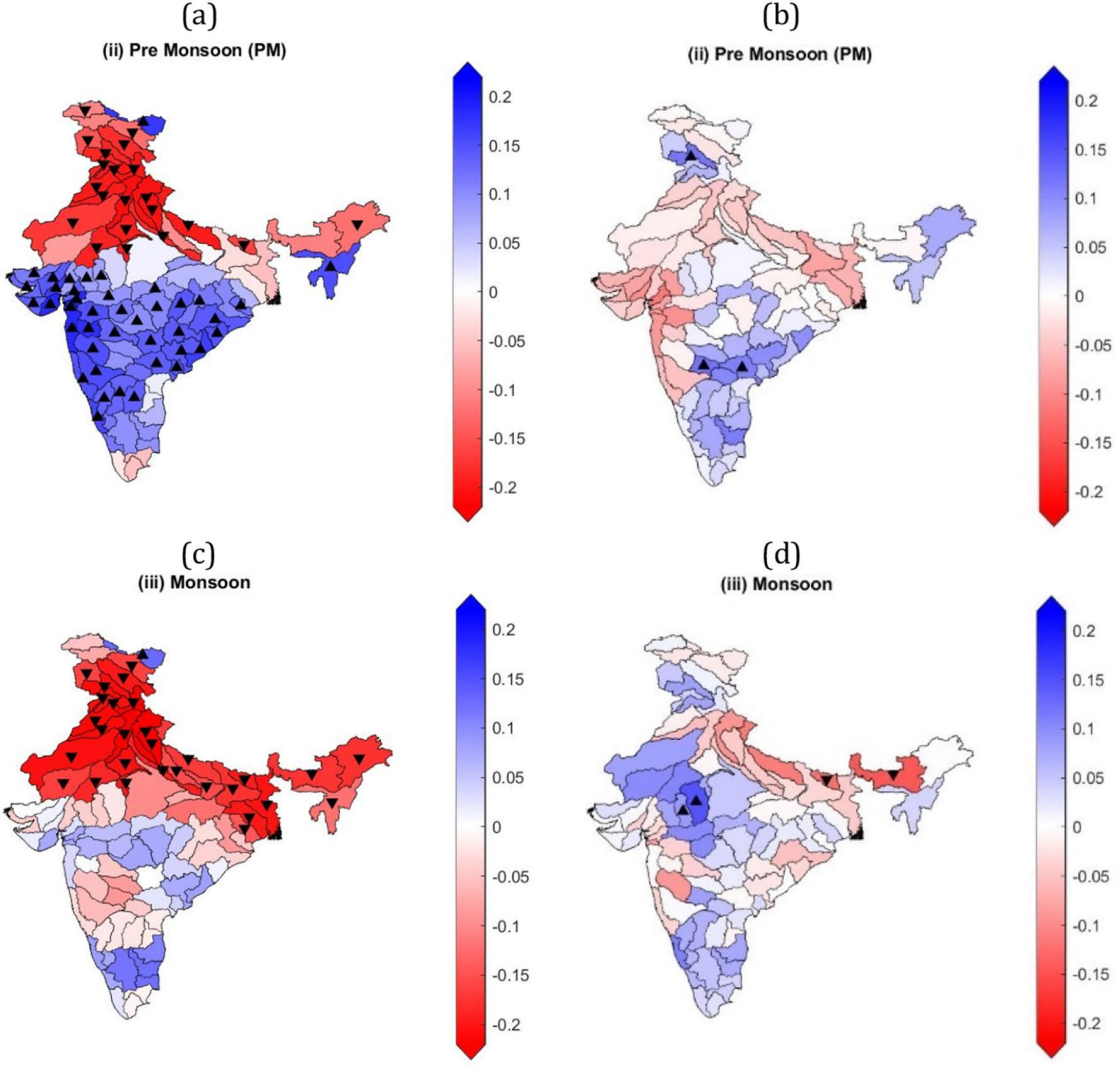
Seasonal CCDI/GRACE-DSI trends using the Mann Kendall trend test and Theil-Sen's slope estimator over major Indian river basins from the Monsoon season. (**a**) GRACE-DSI trends for pre-monsoon season, (**b**) CCDI trends for pre-monsoon season, (**c**) GRACE-DSI trends for monsoon season, and (**d**) CCDI trends for monsoon season, respectively. Colormap over the basin represents the slope obtained from TSA, △ represents a significant increasing trend, and ▽ indicates a significant decreasing trend based on the Mann–Kendall test.

GRACE-DSI's seasonal trends show continuous negative trends for sub-basins of Ganga and Indus throughout the year, while other sub-basins show changing trends (Supplementary Figs. S1 & S2). Sub-basins of Indus and northern sub-basins of Ganga exhibit significant negative GRACE-DSI trends, whereas most of the sub-basins the peninsular river basins show significant positive GRACE-DSI trends for pre-monsoon season (Fig. 5a). This contrasting trend behaviour of the sub-basins in the north and south India need to be examined. The remaining sub-basins show either non-significant positive or negative trends for the pre-monsoon season. However, for CCDI, most sub-basins has either non-significant positive or negative trends for the pre-monsoon season (Fig. 5b). Further for monsoon season, sub-basins of Ganga and Indus exhibit high significant negative GRACE-DSI trends (Fig. 5c). At the same time, only a few sub-basins of Ganga show significant negative CCDI trends for the monsoon season (Fig. 5d). Interestingly, other sub-basins has either non-significant positive or negative trends for both GRACE-DSI and CCDI during monsoon season.

**Figure 6:**
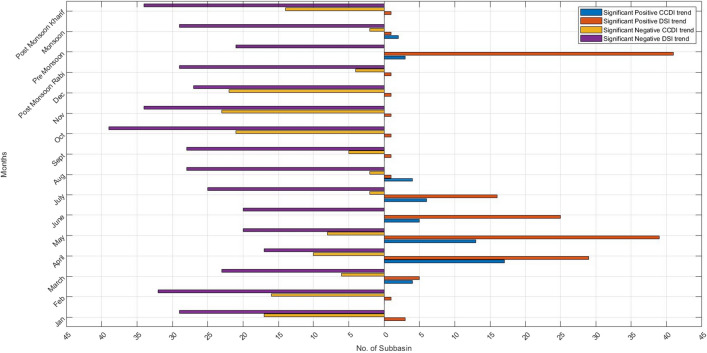
Horizontal bar chart depicting the number of sub-basins showing significant negative trends or significant positive trends for GRACE-DSI and CCDI. The left side is the number of sub-basins showing significant negative trends for CCDI (yellow) and GRACE-DSI (purple), whereas the right side is positive trends for CCDI (blue) and GRACE-DSI (orange).

In Fig. 6, we plot no. of sub-basin showing significant negative or positive trends for GRACE-DSI and CCDI. From Fig. 6, we can observe that most sub-basin shows significant negative trends for GRACE-DSI and CCDI except premonsoon, where GRACE-DSI exhibit both the positive and negative trends. This is mainly because of the higher number of sub-basins in the peninsular India that displayed positive trends during premonsoon. The number of sub-basins showing significant negative trends for GRACE-DSI is also more than that for CCDI. This difference in the characteristics of GRACE-DSI and CCDI may also due to the depletion in TWS in most of the sub-basins for the study period.

## Discussion

In the past, India faces many droughts, which affects millions of people and the country's socio-economic growth. In this study, we have found the drought events over Indian sub-basins using GRACE-DSI and CCDI indices. Our results are consistent with the historic drought of 2000–2003, 2009–2010, 2015–2018^2,35^. GRACE-DSI shows high drought duration events with high severity for most sub-basins, which signifies that terrestrial water storage is depleting^5^. We have compared drought events of both CCDI and GRACE-DSI; we come to know GRACE-DSI characterized drought events are due to the deficit in TWS only. However, when CCDI only shows drought events, it concludes that it is mainly due to the precipitation deficit. But when both CCDI and GRACE-DSI show drought events, it is due to the partial contribution of both deficits in precipitation and TWS. In this study, we also noted that the Indo-Gangetic plain faces many drought events, as verified by the results^3,36^. Rodell et al.^37^ concluded that groundwater is being depleted in the Indian states of Punjab, Rajasthan, and Haryana (including Delhi). Further, the drought indices trend contrast between north and south India, where north India has relatively higher decreasing trend are in congruence with the earlier study^38^, which deals with the terrestrial water storage depletion. Excessive utilization of groundwater for agriculture in rainfall deficit conditions has depleted groundwater in most parts of the world, hence ultimately leading to a drought crisis. Some sub-basin that show significant negative GRACE-DSI trends and significant positive CCDI trends explain that sub-basin that get sufficient rainfall does not show negative CCDI trends. But for sub-basin, which shows significant negative trends for both CCDI and GRACE-DSI, conclude that sub-basin gets low or no rainfall due to which TWS continuously depleting. Further, GRACE-DSI characterized drought events in the sub-basins of Godavari, Krishna, Cauvery is also shown by GGDI during 2008–2010, 2003–2004 & 2015–2016, and 2015–2016 respectively^20^, which informs that groundwater table depletes during this period and leads to drought events. Krishna & Godavari river basins experienced drought during 2003–2004, 2003–2004 & 2008–2010, is also observed by Sinha et al.^19^, which is also explained through GGDI and GRACE-DSI. In their study, Sinha et al.^18^ find that the drought events of 2002 and 2004 are noteworthy in peninsular, west-central, and north-western India. In contrast, the drought event of 2010 is noticeable over central, west-central, and north-western India. During 2016–2018, Southern India faced a severe water crisis^39^, that can also be observed in our study. Mishra^35^ reveals that the drought of the year 2015–2018 affected surface water and groundwater availability in the southern part of India. The meteorological drought of 2015–2018 has the most prolonged drought duration of 41 months. The study further stated that rapid depletion of groundwater and long-term drought could lead to severe problems of water availability in India. The quick deficit in groundwater in north India makes the region vulnerable to drought impacts ^40^.

Satish Kumar et al.^20^ use six drought indices and found that CCDI and GRACE-DSI effectively study drought characteristics. Overall, the CCDI and GRACE-DSI show promising results. When we work on a large scale, both GRACE-DSI and CCDI correlate very much because working on a large scale averages the effect of precipitation. GRACE-based drought indices are very helpful in characterizing the drought in the absence of ground station data. Also, the accuracy of GRACE data is high. Further CCDI includes all aspects of drought occurrences like Meteorological, Agricultural, Hydrological and Anthropogenic activities. Although there are some limitations, like GRACE-data sets only available from April 2002 and GRACE data spatial resolution is also relatively low. Errors in precipitation and TWSA can lead to CCDI or GRACE-DSI index errors. Evaluation of the hydroclimatoligical teleconnections^41,42^ linked with the drought events could be the possible future trajectory of this study.

## Conclusion

In this study, during 2002–2017, the drought characteristics were examined and evaluated using the GRACE-DSI and CCDI as a metric over major river sub-basins in India. The key findings from this study are:

1. GRACE-DSI shows significant negative trends over most of the Indian sub-basins relative to CCDI, indicating that most of the drought events are due to depletion of TWS.
2. The maximum drought duration and drought severity computed by CCDI is 17 months and − 13.4392, respectively for Shyok subbasin.
3. The maximum drought duration and drought severity observed by GRACE-DSI is 26 months and − 44.2835, respectively for the subbasin in the North Ladakh region.
4. Multiple drought events have been observed for the Indo-Gangetic plains between 2002 and 2004, 2009–2014 & 2015–2017.
5. Almost all the sub-basins have shown the drought events for both the CCDI & GRACE-DSI between 2015 and 2017 in congruence with the earlier studies.

Although many subbasins show increase in the drought over the time, there are sub-basins that has multiple drought events in the earlier time period i.e., 2002–2005 as well. Further, trend analysis revealed negative trend in the CCDI & GRACE-DSI values for most of the subbasins with varying magnitudes in different seasons. However, we also need to keep in mind that the study period is only between 2002 and 2017 due to the limited data availability. Also quality of gridded precipitation data is mainly depending on the density of the rain gauges. Thus, these arguments could only be strengthened with increasing observed data quantity & quality over the time.

## Supplementary Information

Supplementary Information.

## Data Availability

The datasets analysed during the current study are available in the IMD (https://imdpune.gov.in/Clim_Pred_LRF_New/Grided_Data_Download.html) & GRACE (https://grace.jpl.nasa.gov/data/get-data/jpl_global_mascons/) websites.
