# Supplementary material for: Drought characterization over Indian sub-continent using GRACE-based indices
Source: Sci Rep. 2022 Sep 14;12:15432. doi: 10.1038/s41598-022-18511-2 (PMC9474877; doi:10.1038/s41598-022-18511-2)
Supplement: Supplementary file 1 — Supplementary Information. [file 41598_2022_18511_MOESM1_ESM.docx]

**Drought Characterization over Indian sub-continent using GRACE-based indices**

Shivam Rawat, Abinesh Ganapathy, and Ankit Agarwal^*^

Department of Hydrology, Indian Institute of Technology Roorkee, 247667, India

*Correspondence to: Ankit Agarwal (ankit.agarwal@hy.iitr.ac.in)

**Supplementary Material**

**Table S1.** Catchment area details of Indian Basins/Sub-basins (Source: India-WRIS)

| **Notation** | **Basin/Sub-basin Name** | **Area (sq.km)** |
| --- | --- | --- |
| 1 | Indus  (1a) Gilgit,  (1b) Indus lower,  (1c) Indus upper,  (1d) Shyok,  (1e) Jhelum,  (1f) Chenab,  (1g) Ravi,  (1h) Beas,  (1i) Sutlaj upper,  (1j) Sutlaj lower,  (1k) Ghaghar and others | 453931.87  27101.85  23891.72  46268.85  38724.57  29196.01  29974.29  13566.95  19138.22  21425.47  38578.38  49984.26 |
| 2 | Ganga  (2a) Above Ramganga confluence,  (2b) Ramganga,  (2c) Yamuna upper,  (2d) Yamuna middle,  (2e) Yamuna lower,  (2f) Ghaghara,  (2g) Upstream of Gomti confluence to Muzaffarnagar,  (2h) Gomti,  (2i) Ghaghara confluence to Gomti confluence,  (2j) Gandak and others,  (2k) Kosi,  (2l) Bhagirathi and others,  (2m) Damodar,  (2n) Sone,  (2o) Tons,  (2p) Chambal upper,  (2q) Chambal lower,  (2r) Kali Sindh and others up to the confluence with Parbati,  (2s) Banas | 808334.44  38792.4  30811.48  35584.95  34830.46  125084.38  58728.53  29381.01  29618.82  26403.75  56573.83  19037.96  63059.31  42050.58  64789.32  16857.08  25511.32  11067.89  48511.89  51639.43 |
| 3 | Brahmaputra  (3a) Brahmaputra upper,  (3b) Brahmaputra lower | 186421.6  99040.33  87381.27 |
| 4 | Barak and others | 45622.41 |
| 5 | Godavari  (5a) Godavari upper,  (5b) Godavari middle,  (5c) Godavari lower,  (5d) Manjra,  (5e) Pranhita and others,  (5f) Wardha,  (5g) Weinganga,  (5h) Indravati | 302063.93  21469.99  36289.01  43821.19  29485.75  36108.58  46237.65  49677.35  38974.42 |
| 6 | Krishna  (6a) Bhima upper,  (6b) Bhima lower,  (6c) Krishna upper,  (6d) Krishna middle,  (6e) Krishna lower,  (6f) Tungabhadra upper,  (6g) Tungabhadra lower | 254743.31  44807.62  23649.56  54498.4  22286.59  39438.74  28520.15  41542.29 |
| 7 | Cauvery  (7a) Cauvery upper,  (7a) Cauvery middle,  (7c) Cauvery lower | 85624.44  10961.84  57284.09  17378.51 |
| 8 | Subarnarekha | 25792.16 |
| 9 | Brahmani and Baitarni  (9a) Brahmani,  (9b) Baitarni | 51893.68  37649.47  14244.22 |
| 10 | Mahanadi  (10a) Mahanadi upper,  (10b) Mahanadi middle,  (10c) Mahanadi lower | 139659.15  29794.4  51877.65  57987.1 |
| 11 | Pennar  (11a) Pennar upper,  (11b) Pennar lower | 54243.43  36251.13  17992.3 |
| 12 | Mahi  (12a) Mahi upper,  (12b) Mahi lower | 38336.8  24957.54  13379.26 |
| 13 | Sabarmati  (13a) Sabarmati upper,  (13b) Sabarmati lower | 30678.59  19853.1  10825.49 |
| 14 | Narmada  (14a) Narmada upper,  (14b) Narmada middle,  (14c) Narmada lower | 92670.51  43192.22  40580.48  8897.8 |
| 15 | Tapi  (15a) Tapi upper,  (15b) Tapi middle,  (15c) Tapi lower | 63922.91  28053.09  31759.69  4110.13 |
| 16 | West flowing rivers from Tapi to Tadri  (16a) Bhatsol and others,  (16b) Vasishti and others | 55940  29349.48  27477.72 |
| 17 | West flowing rivers from Tadri to Kanyakumari  (17a) Netravati and others,  (17b) Varrar and others,  (17c) Periyar and others | 56177  18759.52  14163.19  21893.97 |
| 18 | East flowing rivers between Mahanadi and Godavari  (18a) Vamsadhara and others,  (18b) Nagvati and others | 46243.06  21867.4  24375.66 |
| 19 | East flowing rivers between Godavari and Krishna | 10345.16 |
| 20 | East flowing rivers between Krishna and Pennar | 23335.82 |
| 21 | East flowing rivers between Pennar and Cauvery  (21a) Palar and others,  (21b) Ponnaiyar and others | 63646.21  35385.35  28260.85 |
| 22 | East flowing rivers South of Cauvery  (22a) Pamba and others,  (22b) Vaippar and others | 38646.11  18302.72  20343.39 |
| 23 | West flowing rivers of Kutch and Saurashtra, including Luni  (23a) Luni upper,  (23b) Luni lower,  (23c) Saraswati,  (23d) Drainage of Rann,  (23e) Bhadar and other west-flowing rivers,  (23f) Shetranjuli and other east-flowing rivers | 184441.06  79886.74  19735.35  27674.11  21035.42  17936.32  18173.13 |
| 24 | Area of inland drainage in Rajasthan | 139917.03 |
| 25 | Area of North Ladakh not drainage into Indus Basin | 29238.78 |


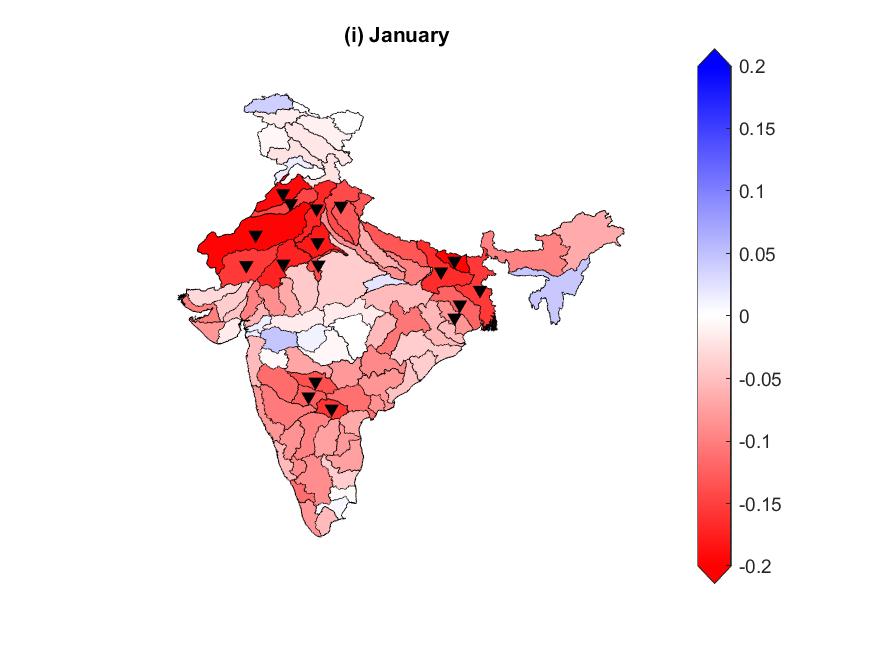

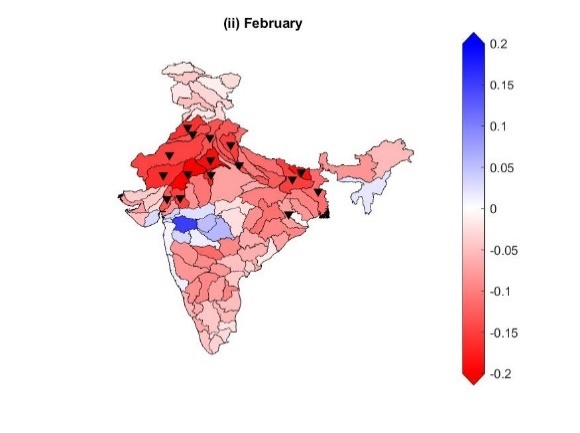

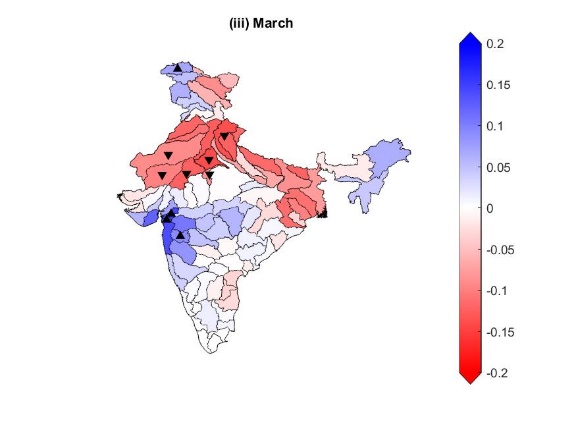


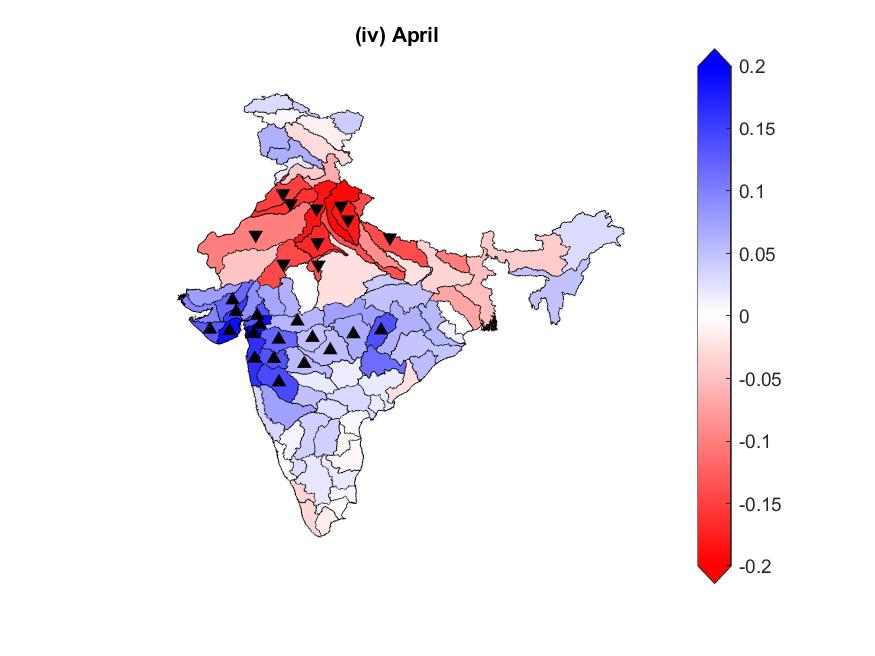

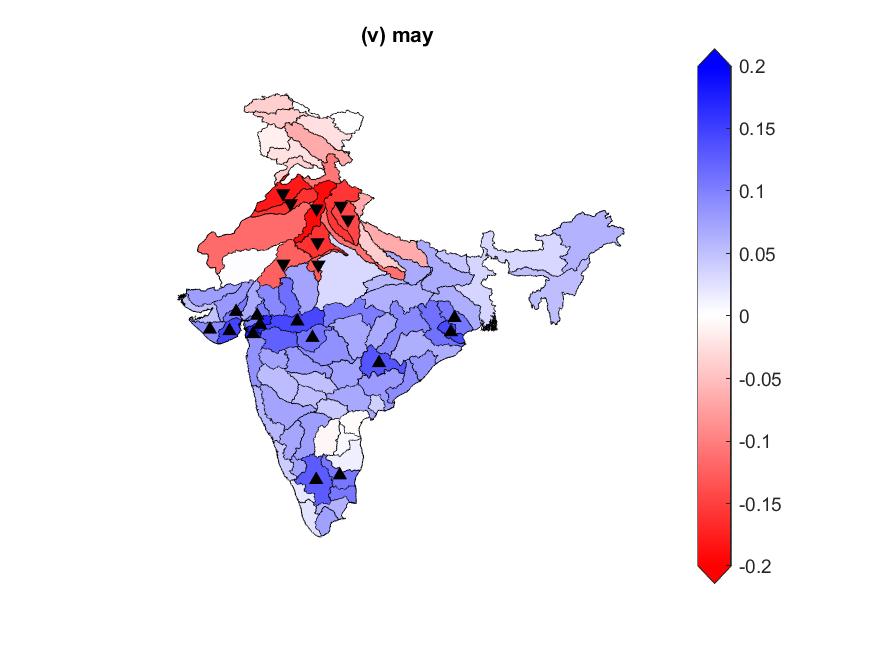

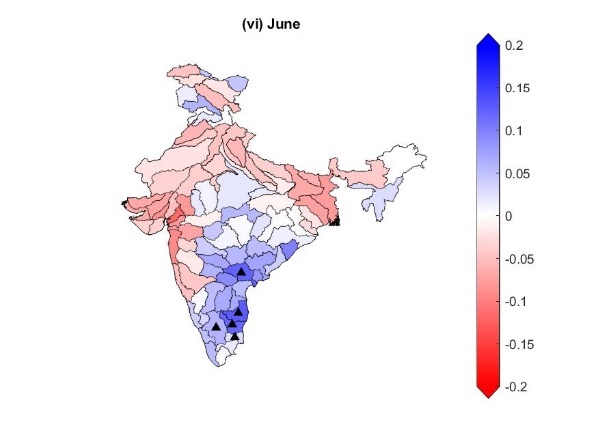


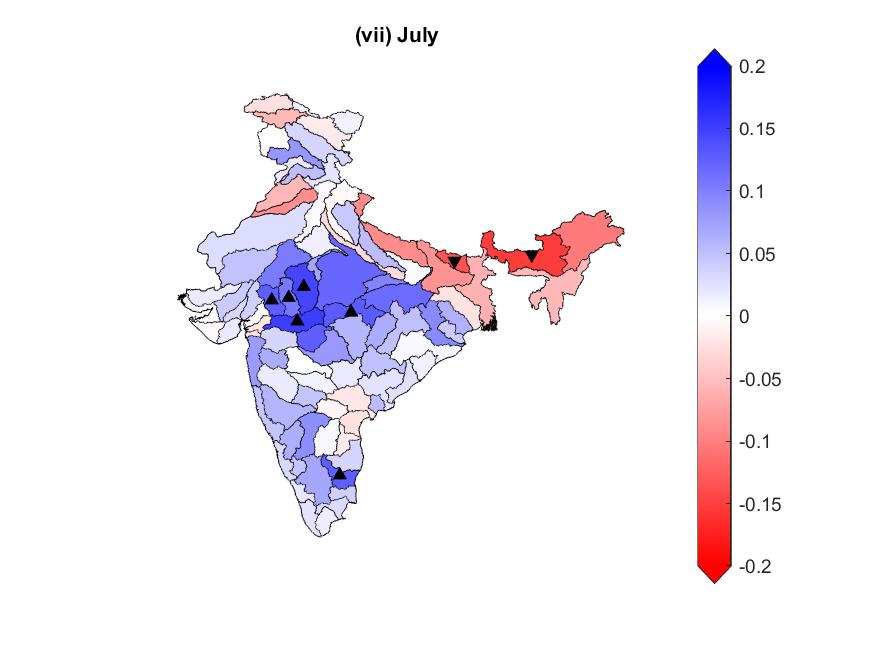

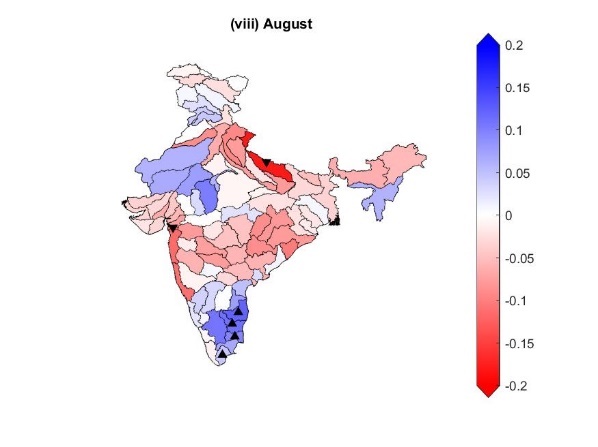

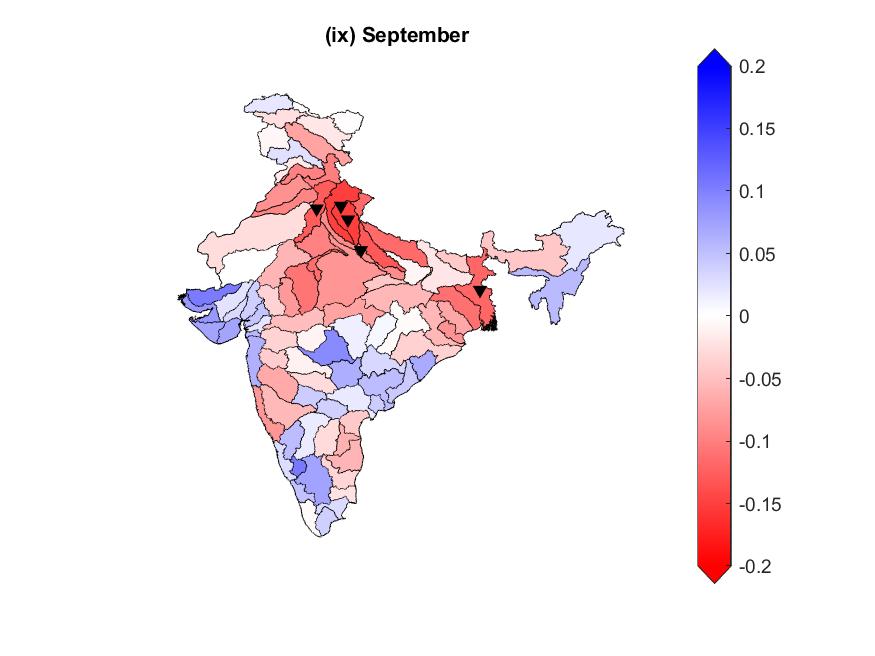


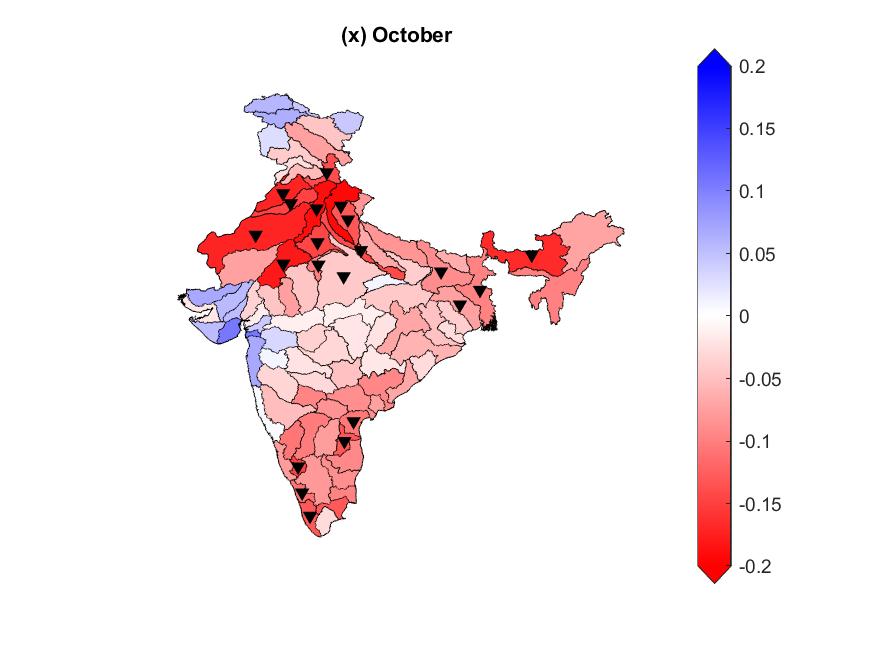

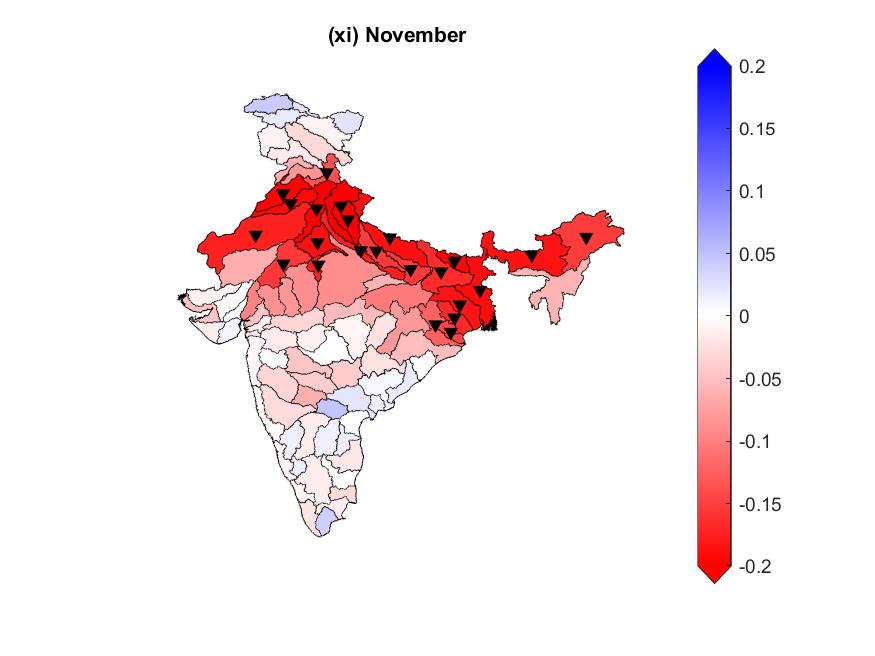

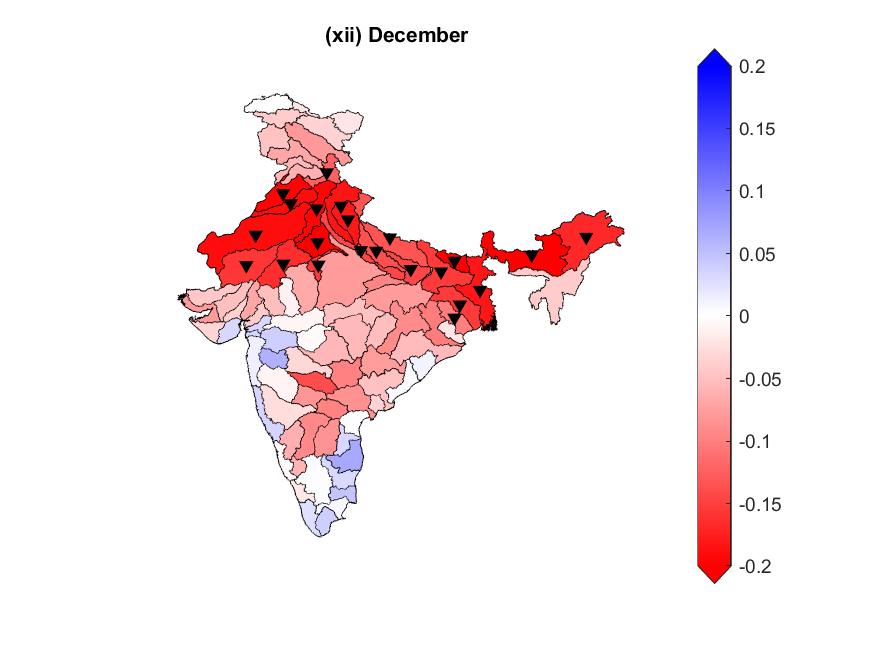


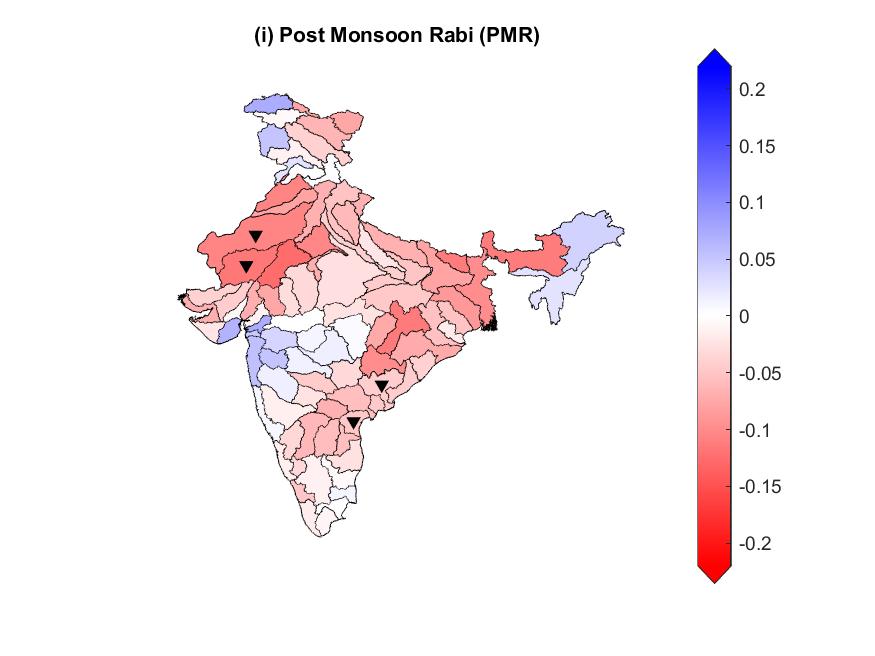

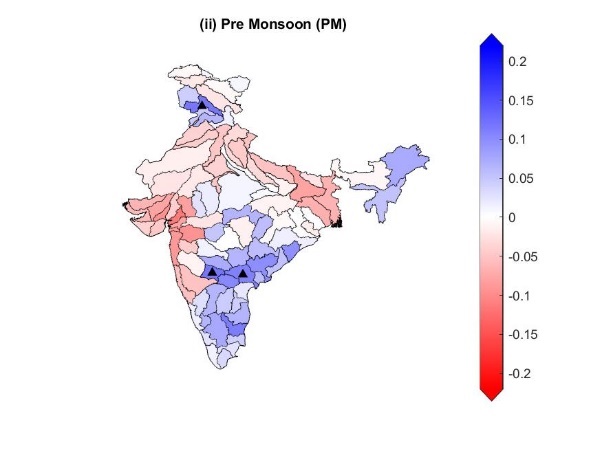

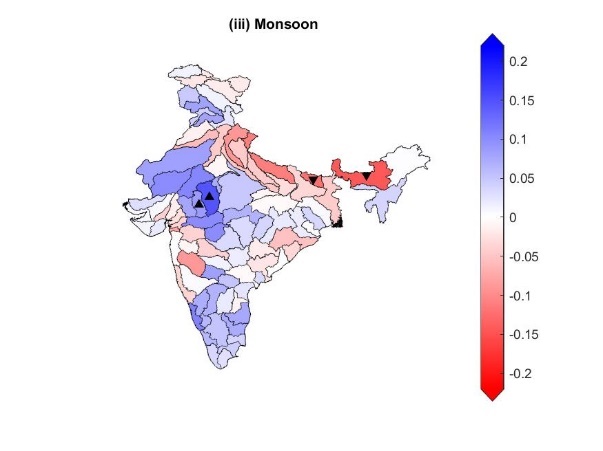


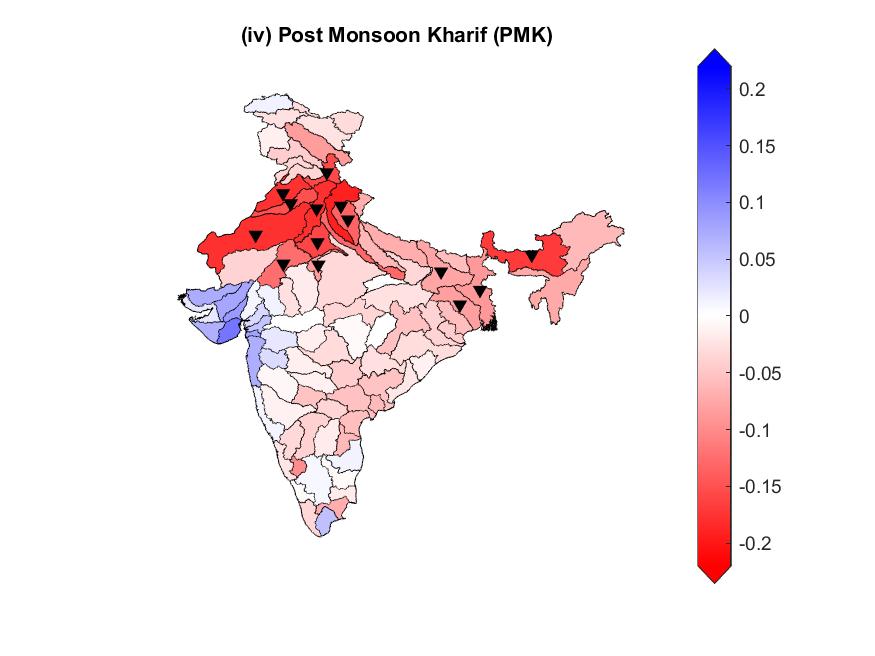


**Figure S1.** Monthly and Seasonal CCDI trends using the Mann Kendall trend test and Theil-Sen's slope estimator over major Indian river basins for individual months from January to December indicated as S2(i)-(xii) respectively and season from Post Monsoon Rabi to Post Monsoon Kharif indicated as S2(i)-(iv) respectively. Colormap over the basin represents the slope obtained from TSA, △ represents a significant increasing trend, and ▽ indicates a significant decreasing trend based on the Mann-Kendall test.


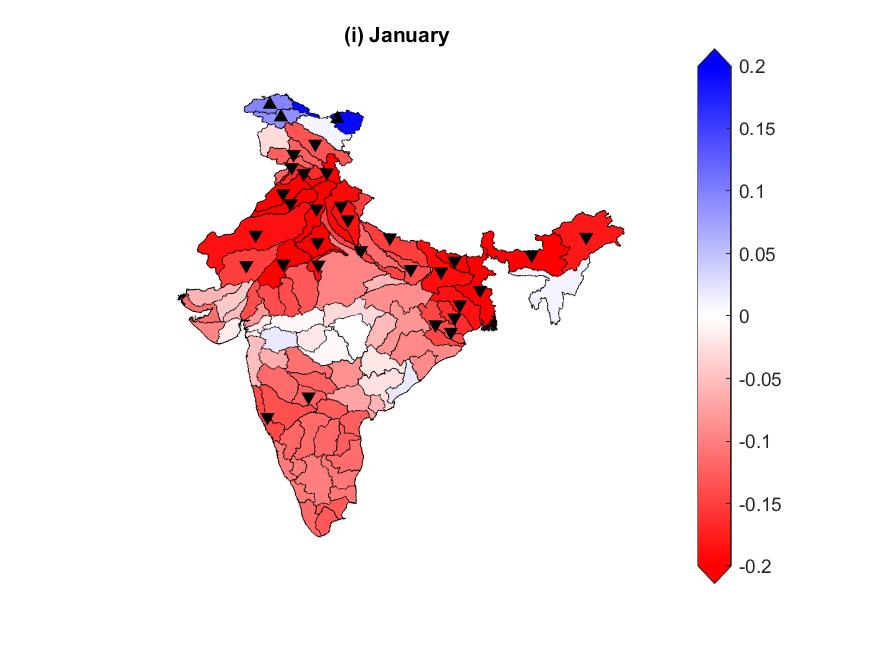

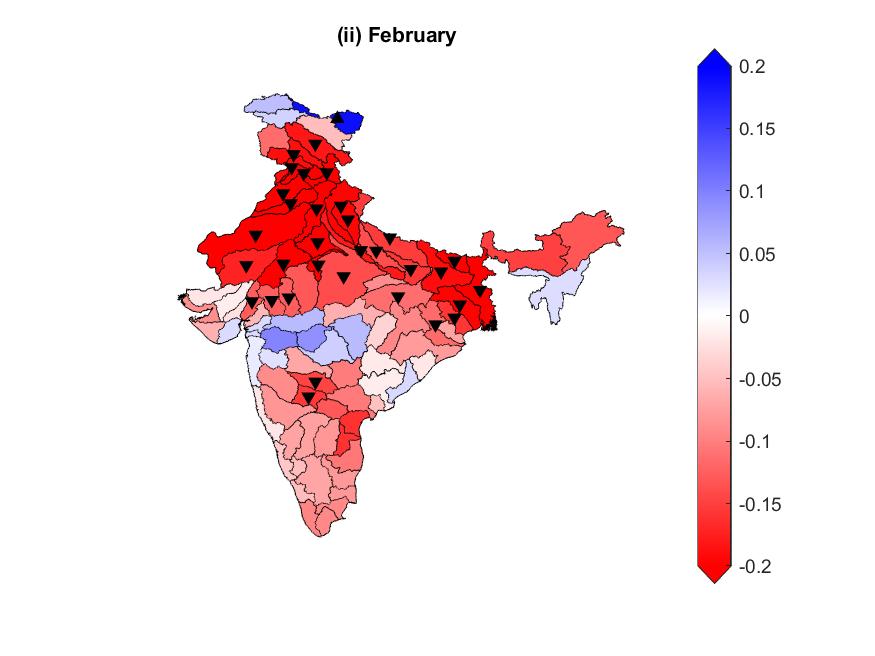

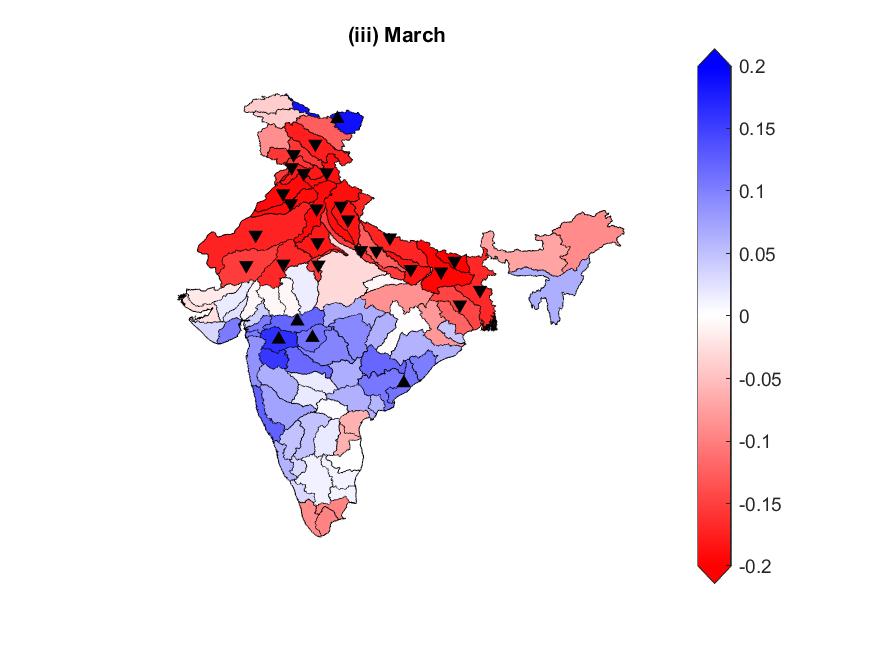


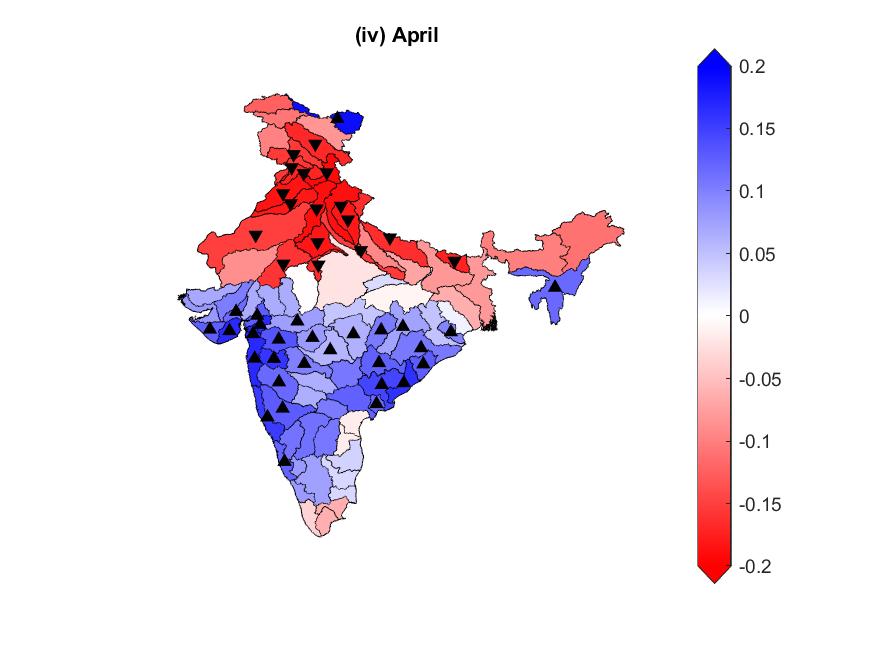

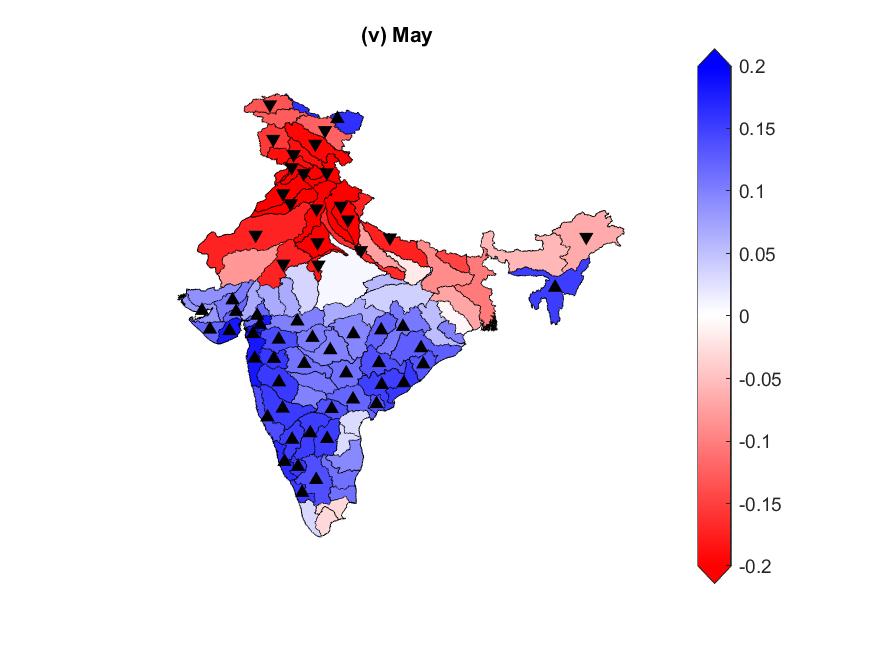

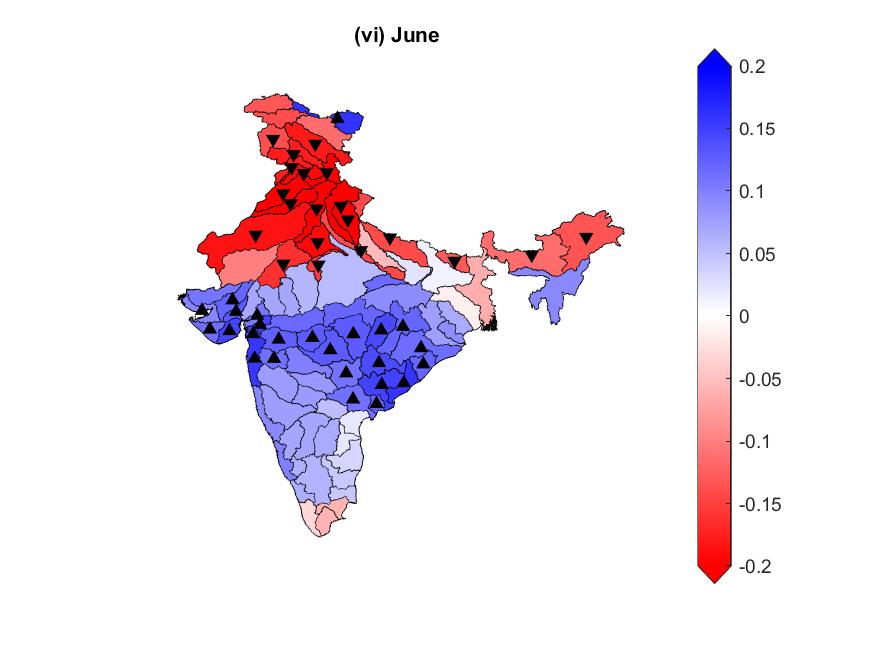


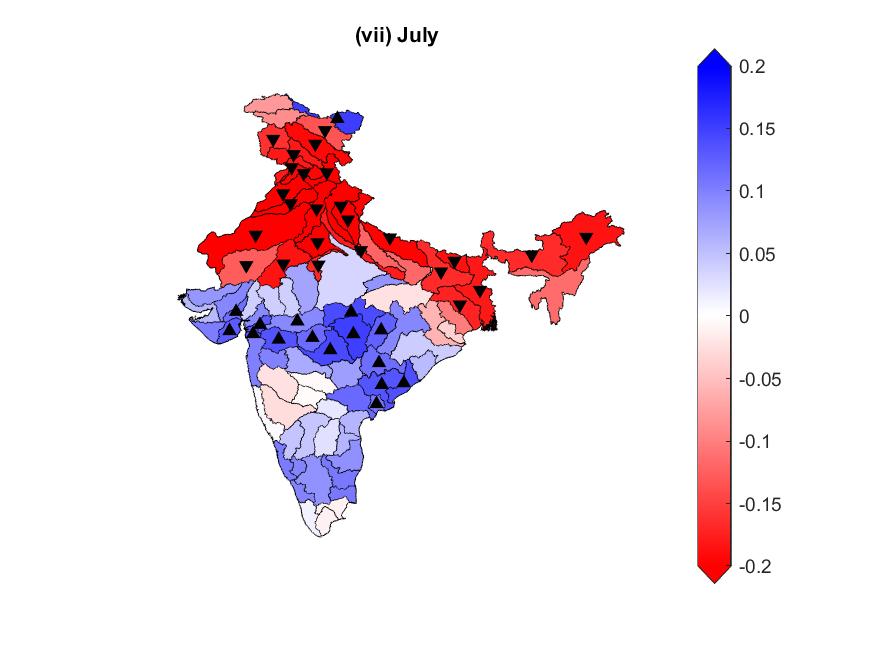

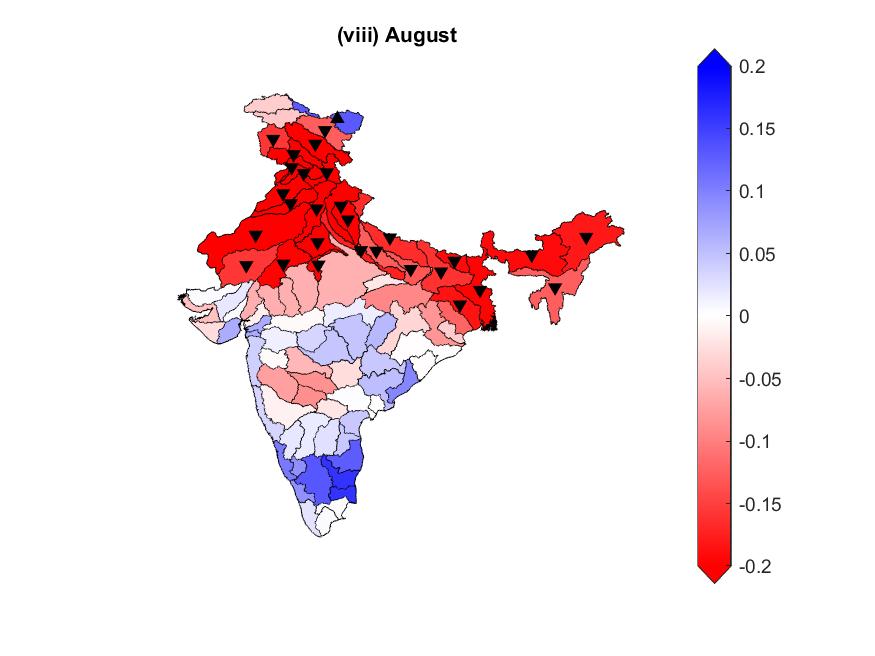

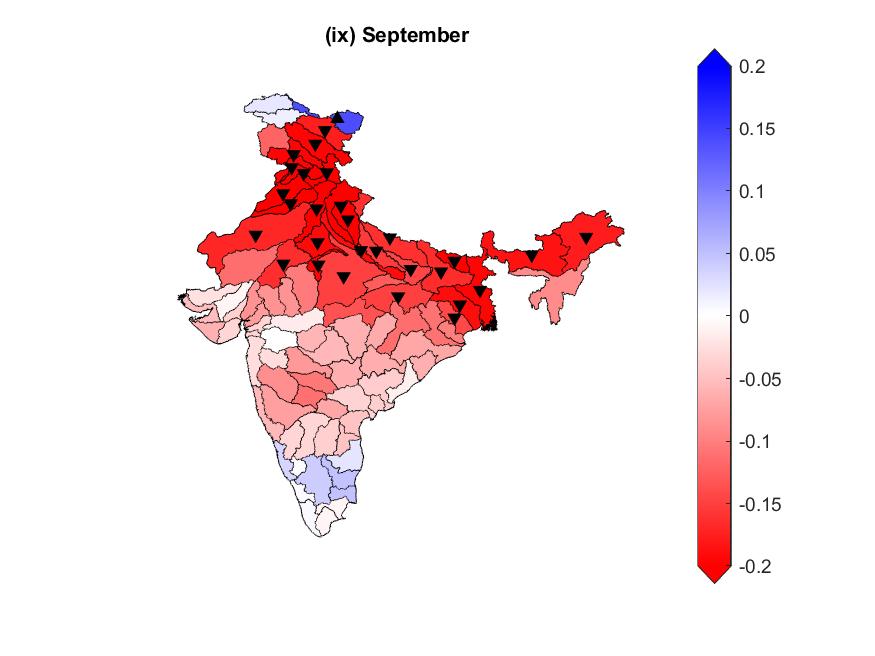


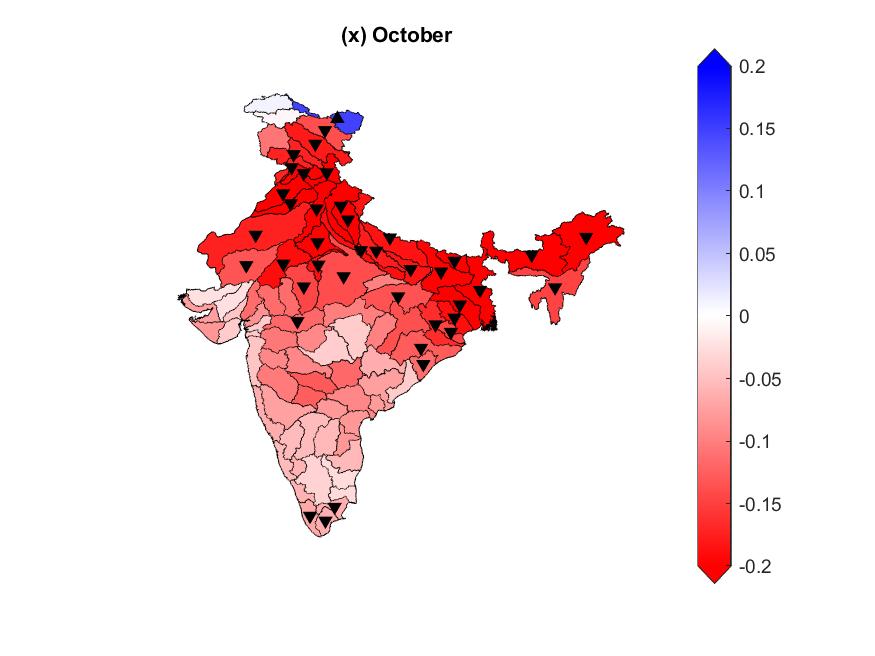

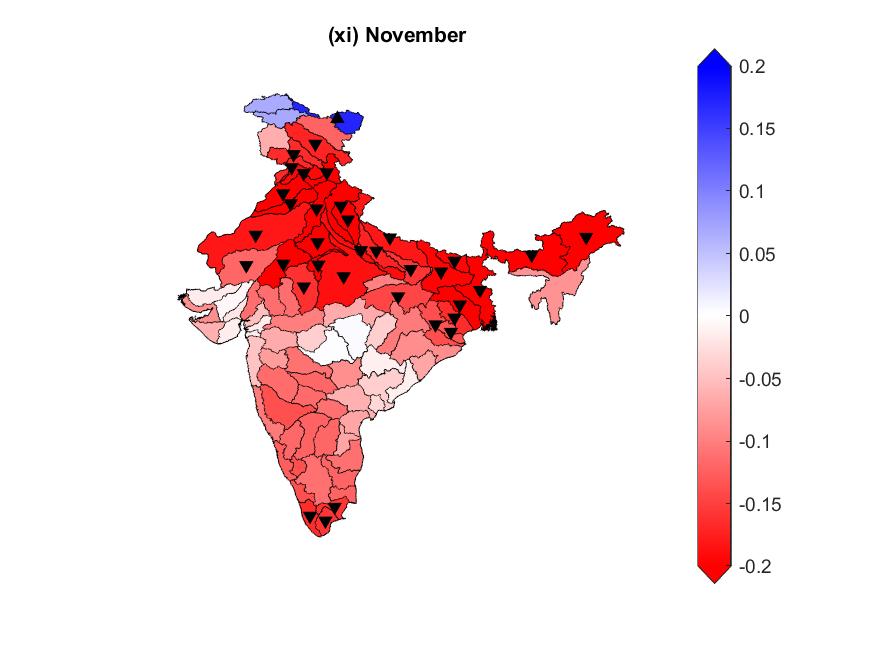

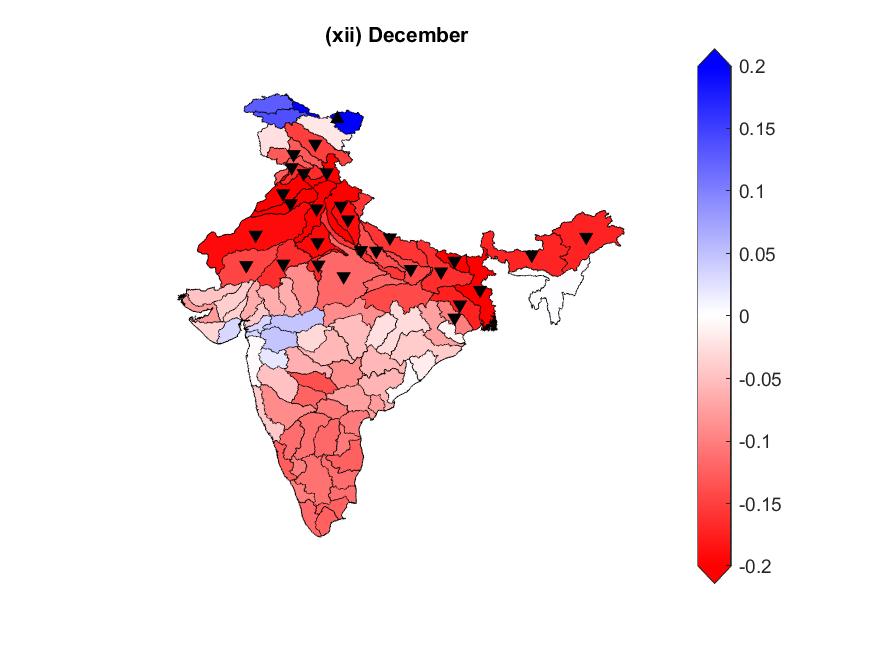


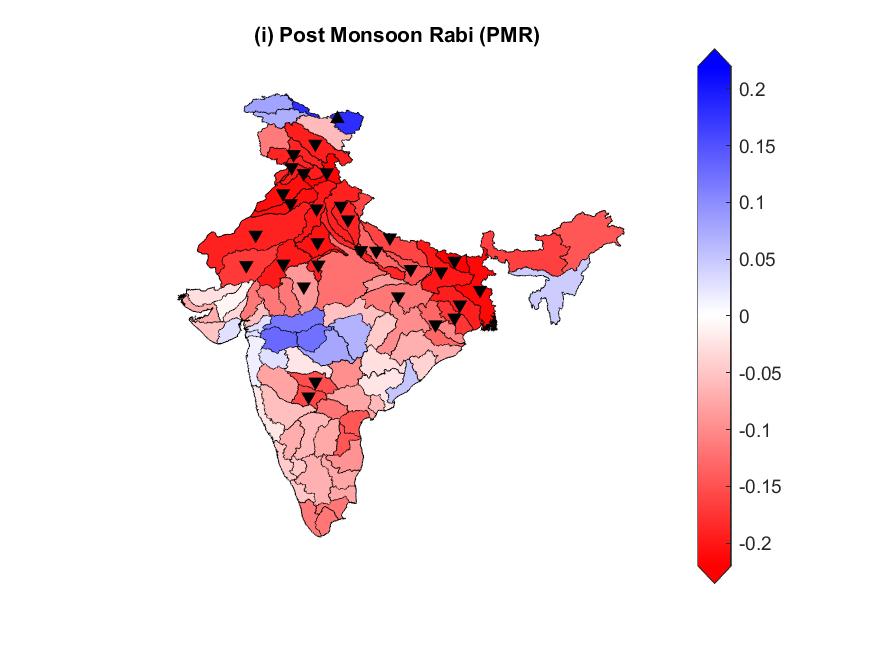

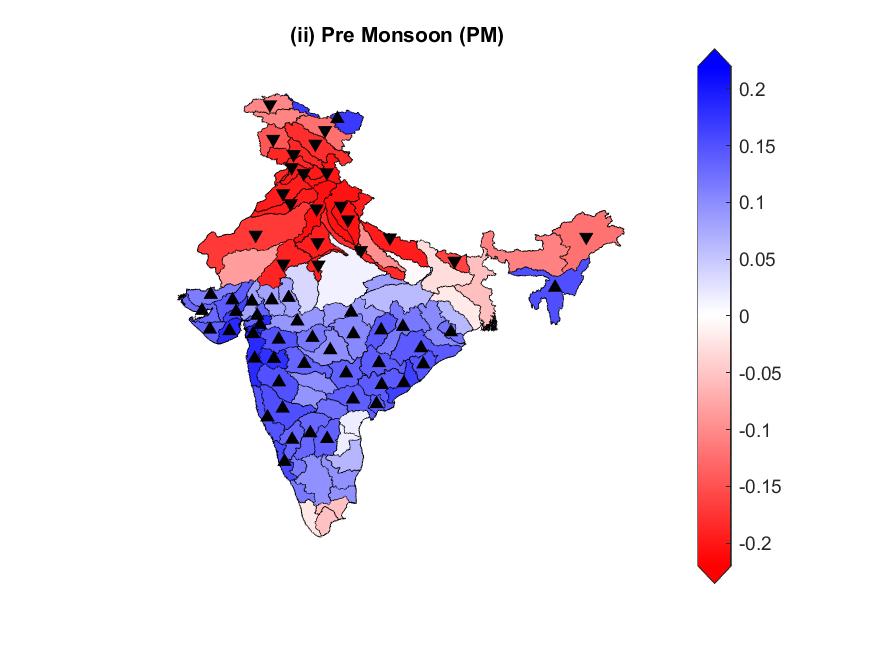

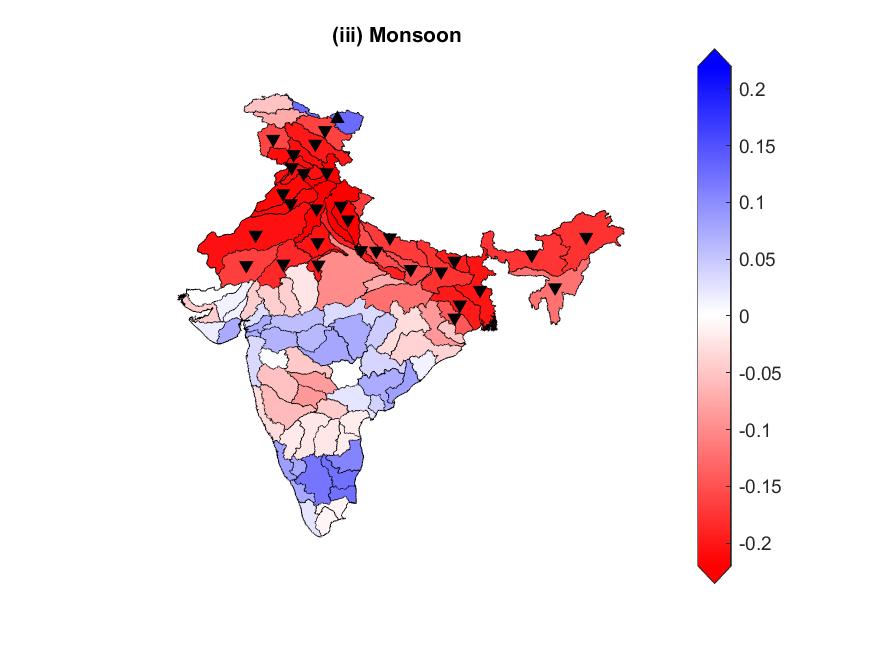


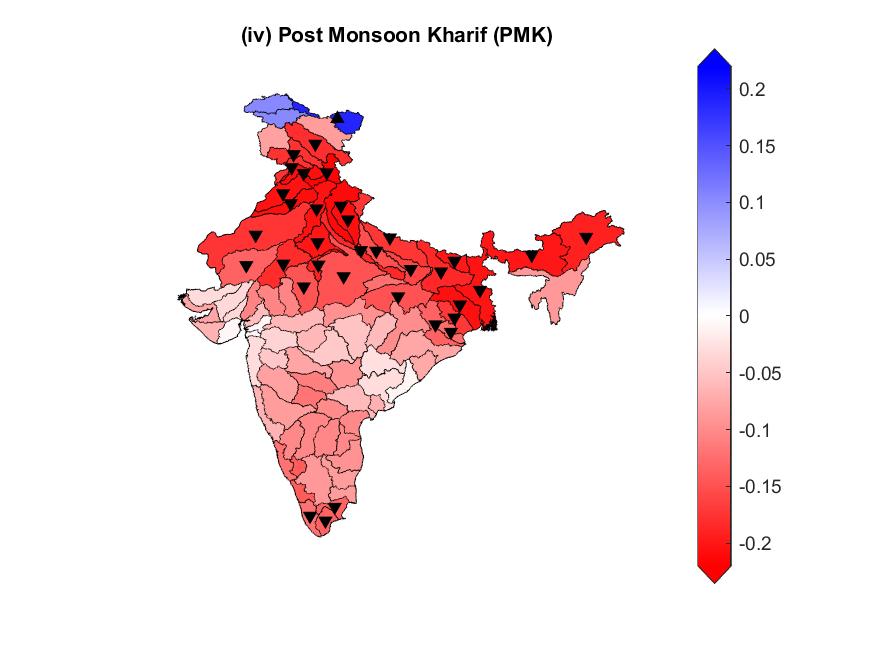


**Figure S2.** Monthly and Seasonal GRACE-DSI trends using the Mann Kendall trend test and Theil-Sen's slope estimator over major Indian river basins for individual months from January to December indicated as S3(i)-(xii) respectively and season from Post Monsoon Rabi to Post Monsoon Kharif indicated as S3(i)-(iv) respectively. Colormap over the basin represents the slope obtained from TSA, △ represents a significant increasing trend, and ▽ indicates a significant decreasing trend based on the Mann-Kendall test.

(I)


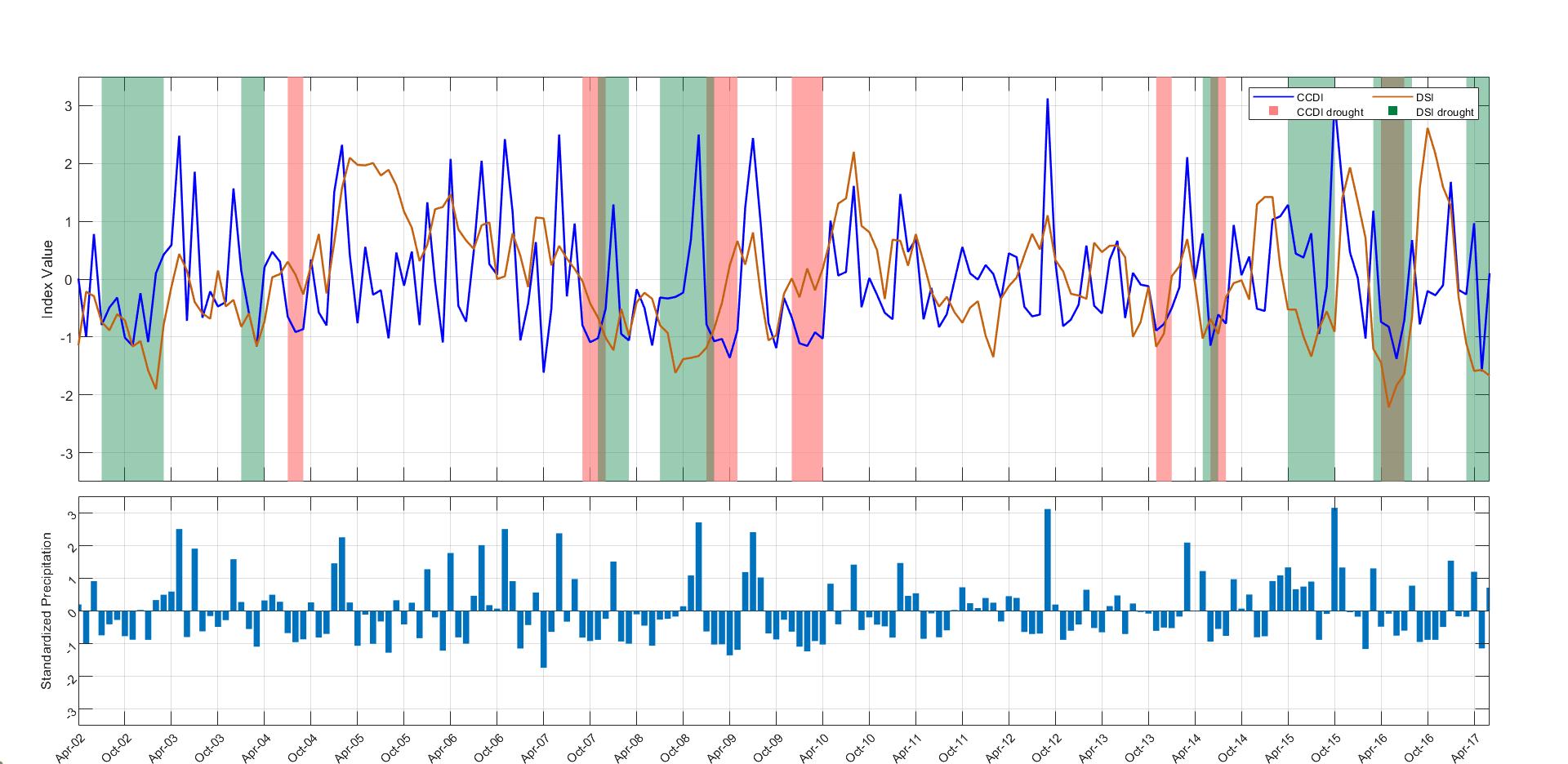


(II)
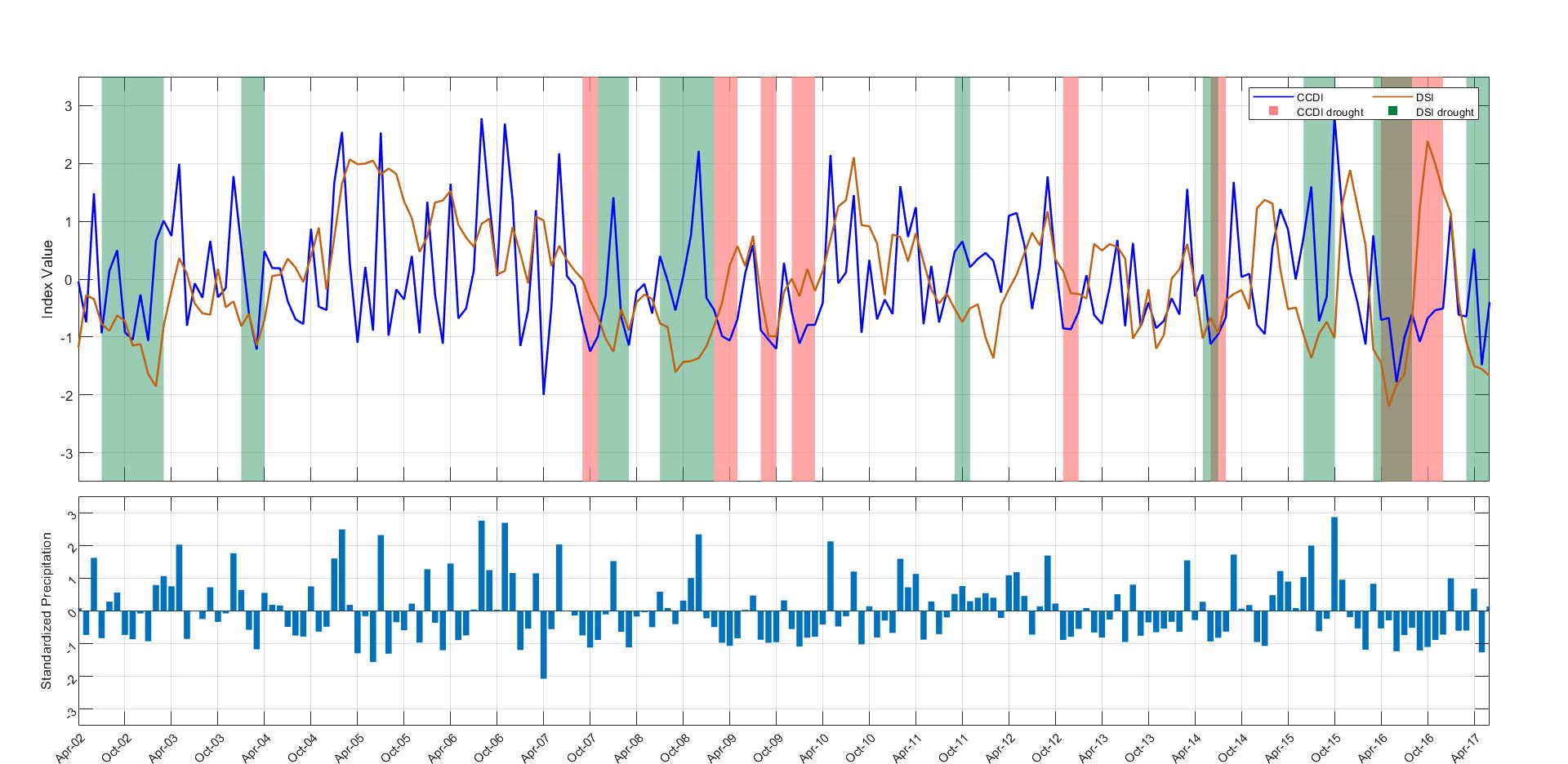


(III)
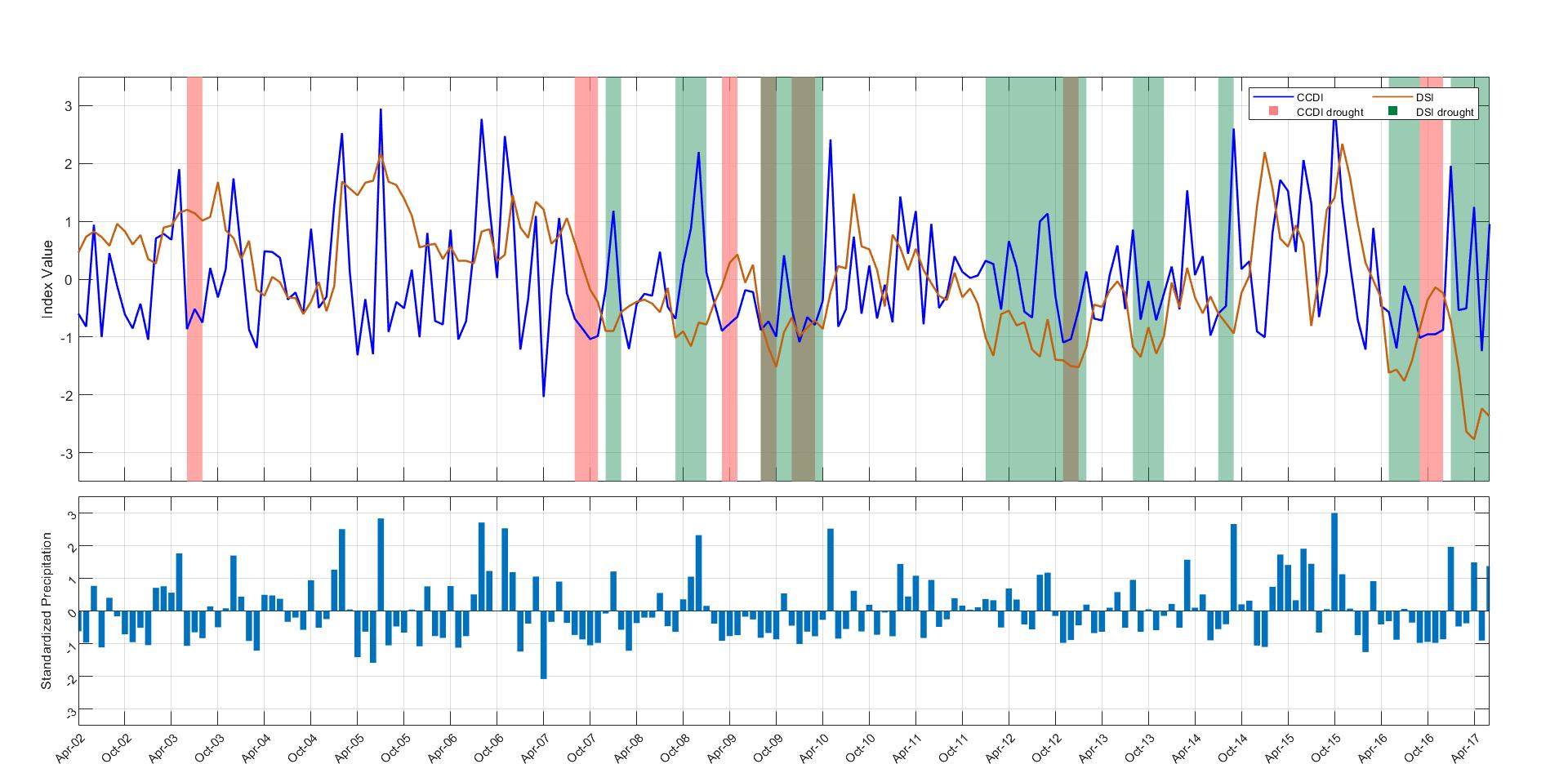


(IV)


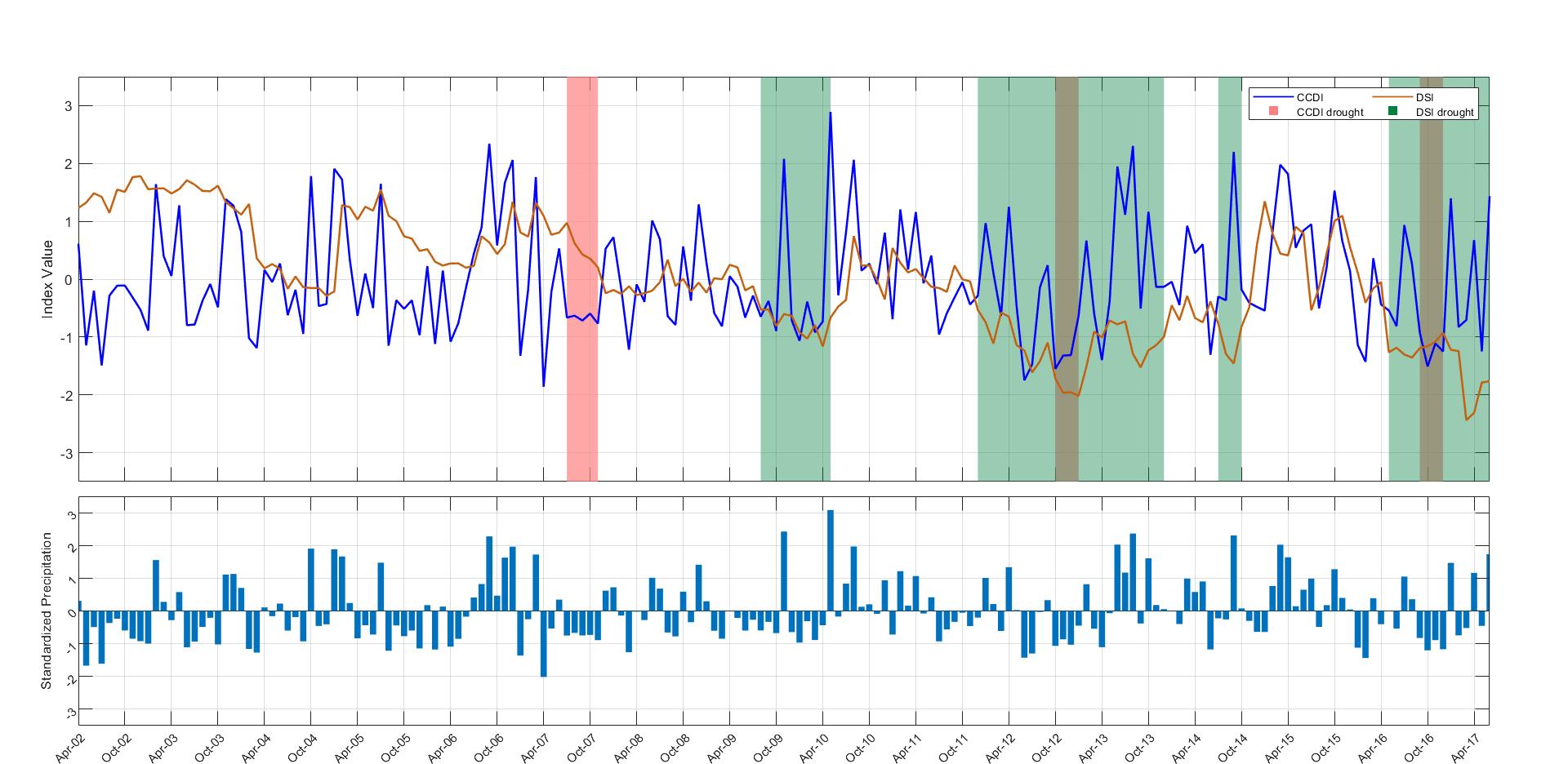


(V)


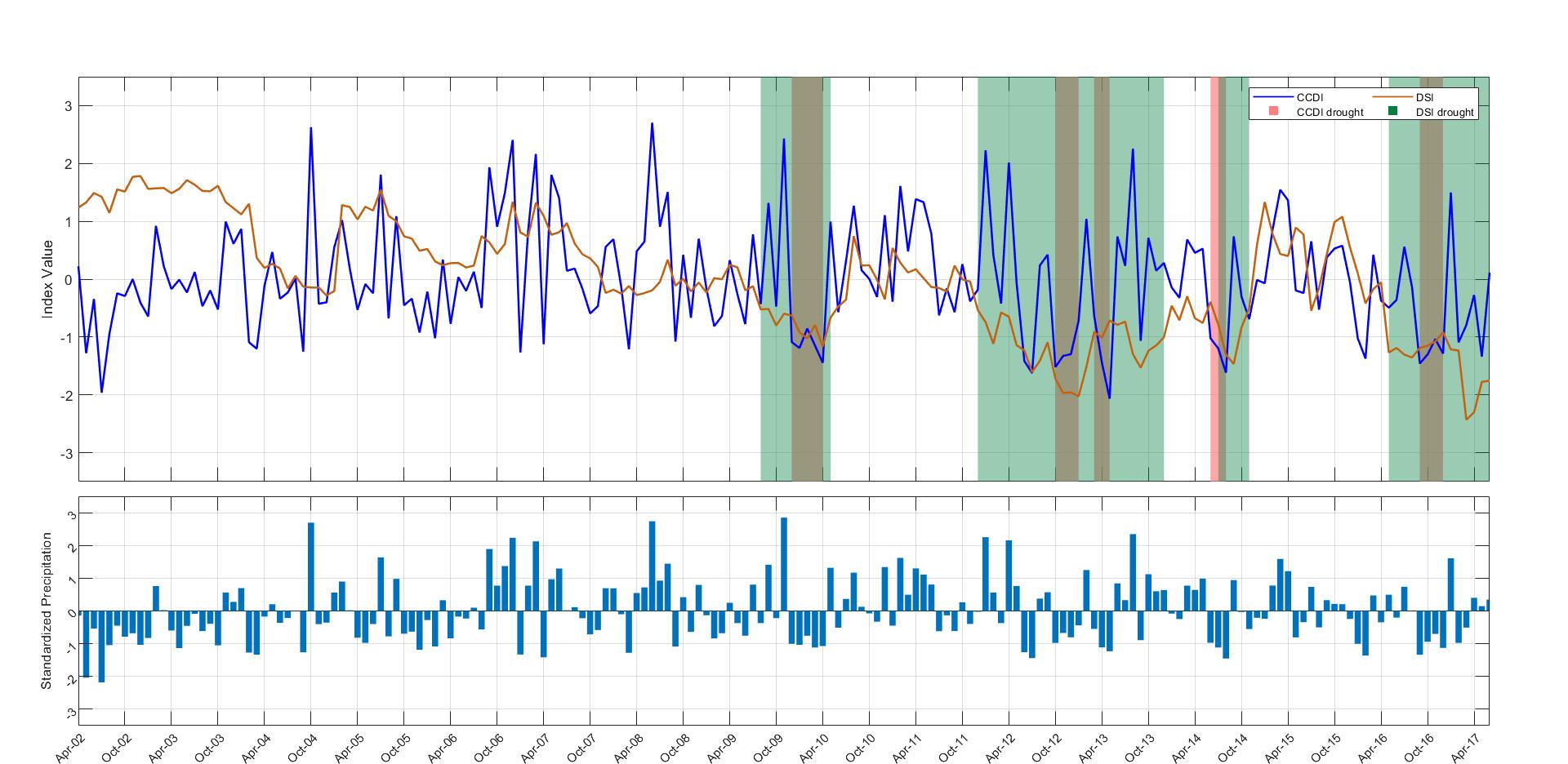


(VI)


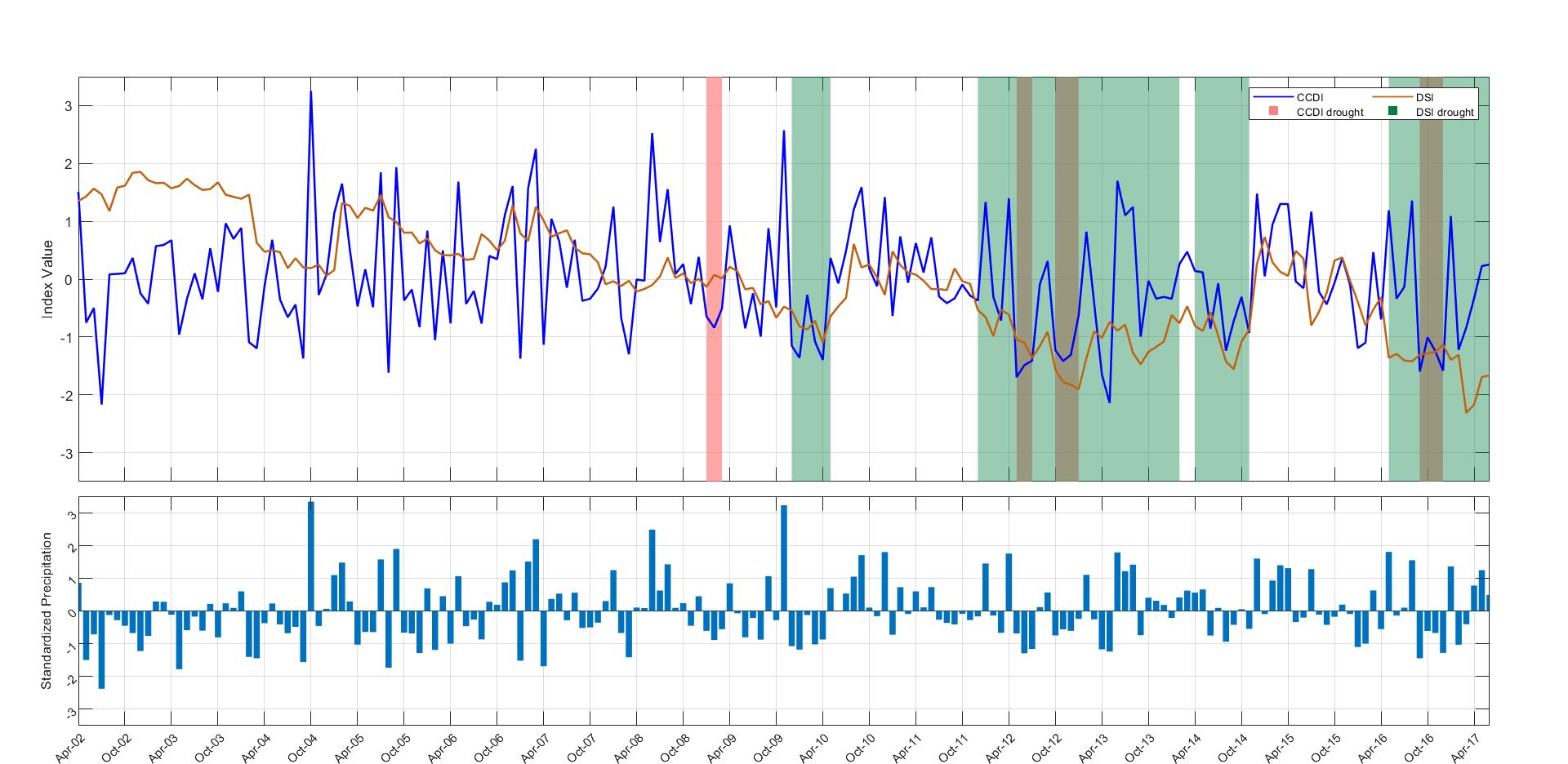


(VII)


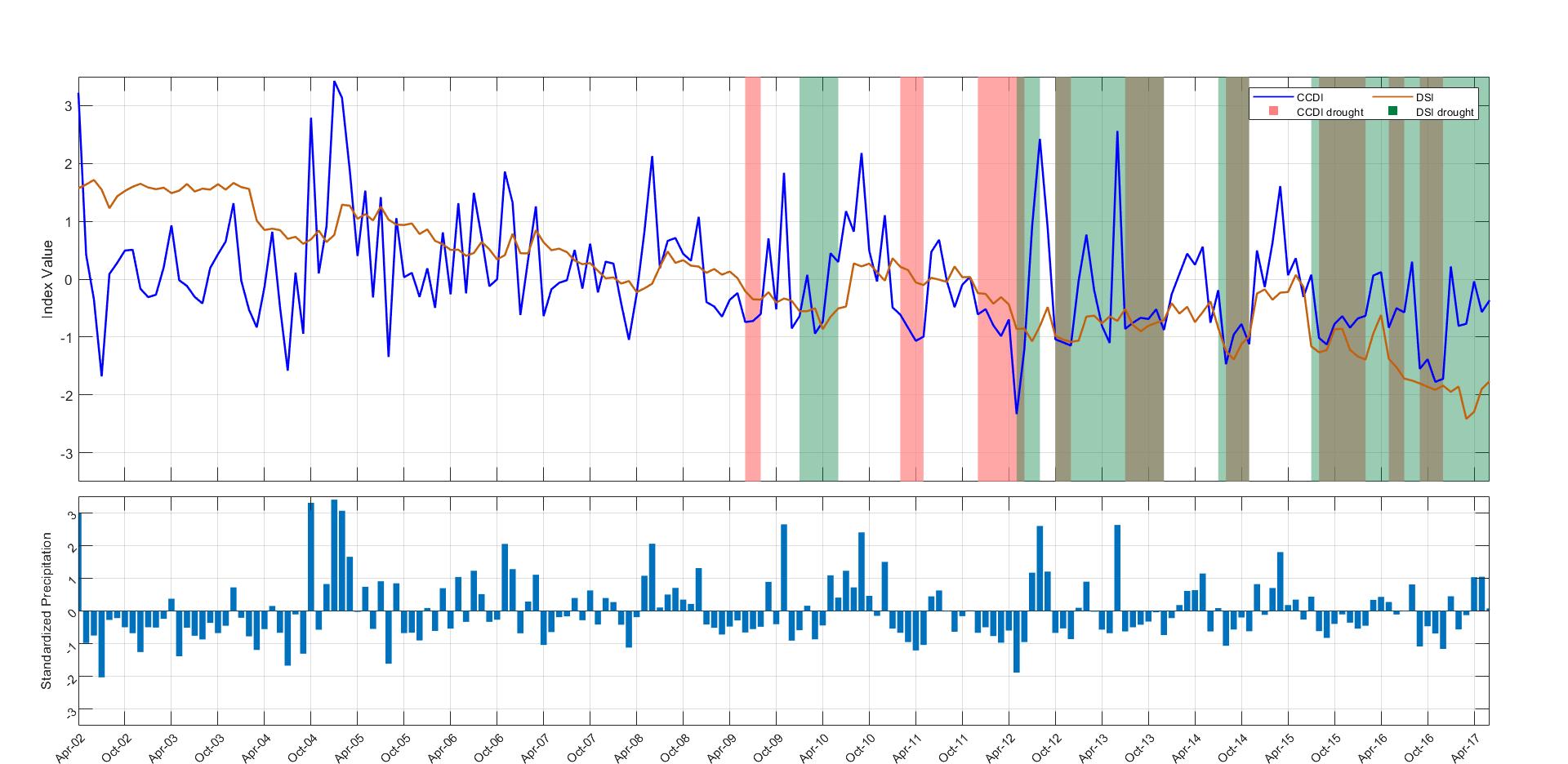


(VIII)


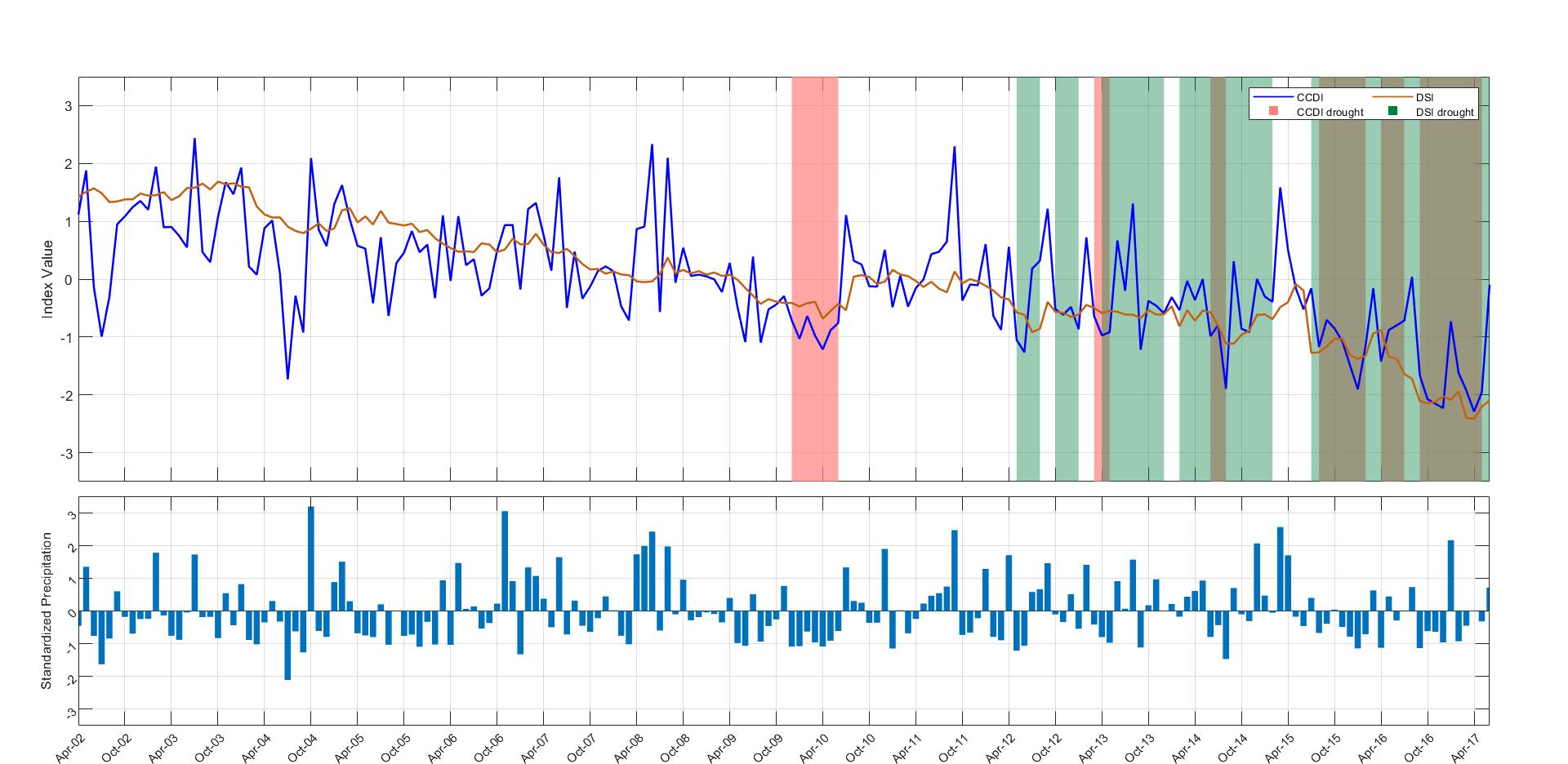


(IX)


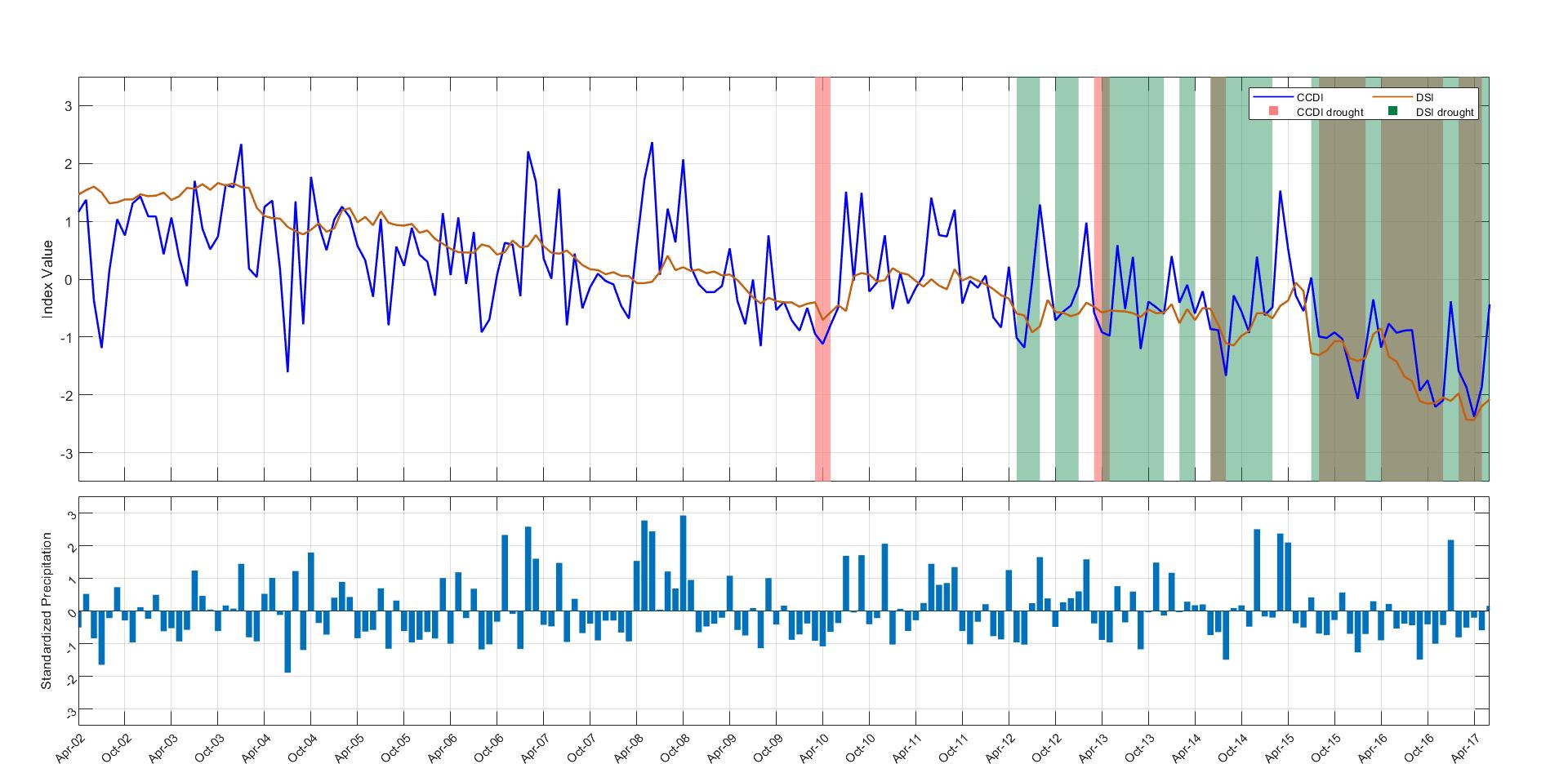


(X)


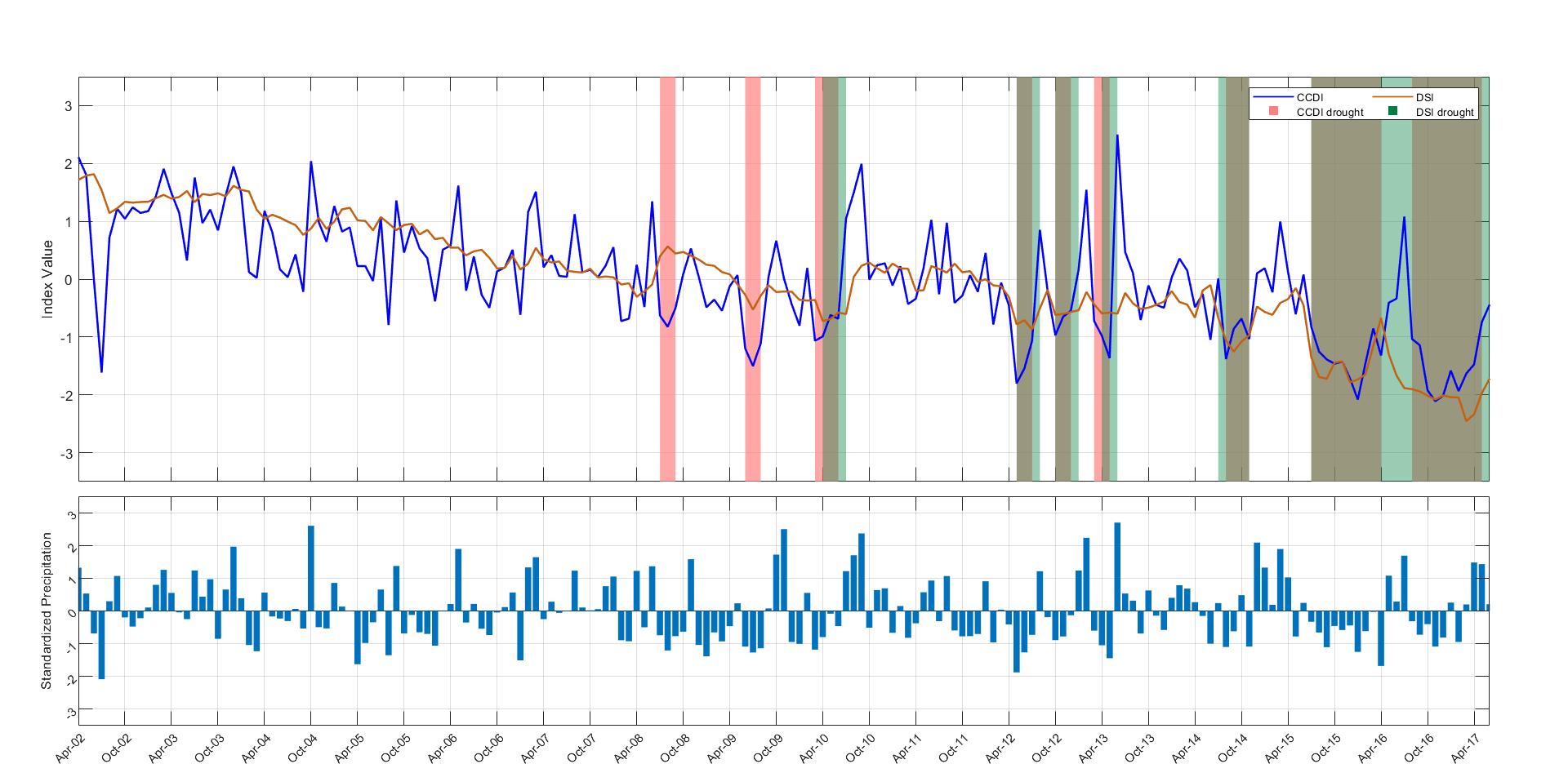


(XI)


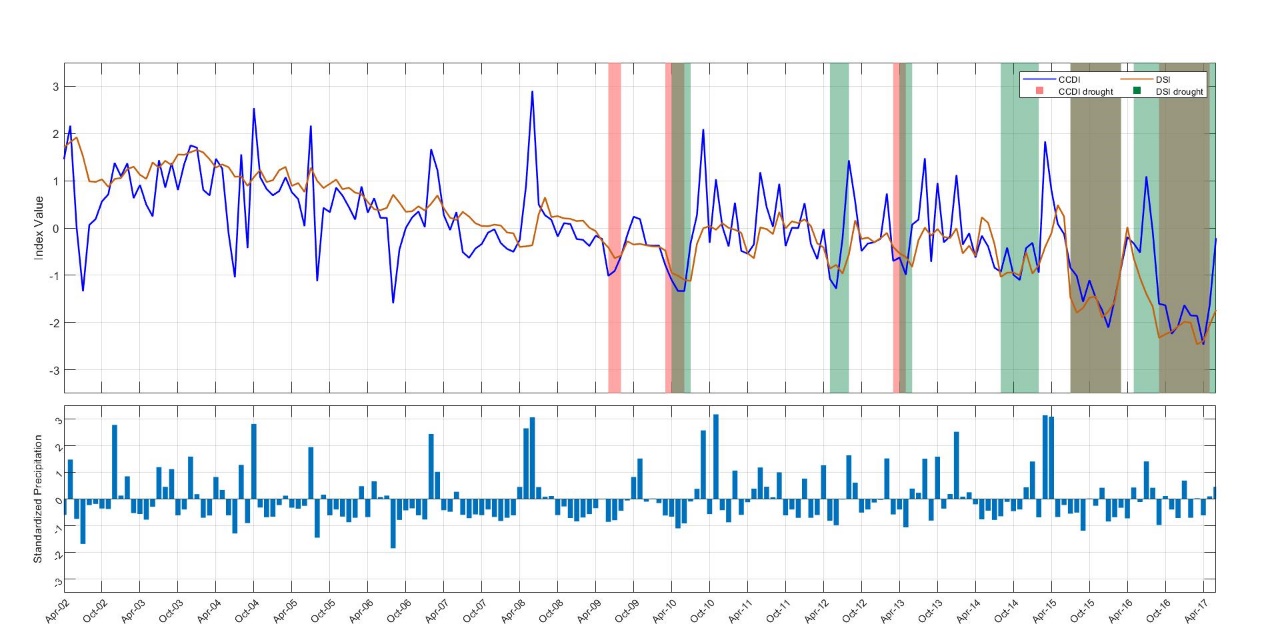


(XII)


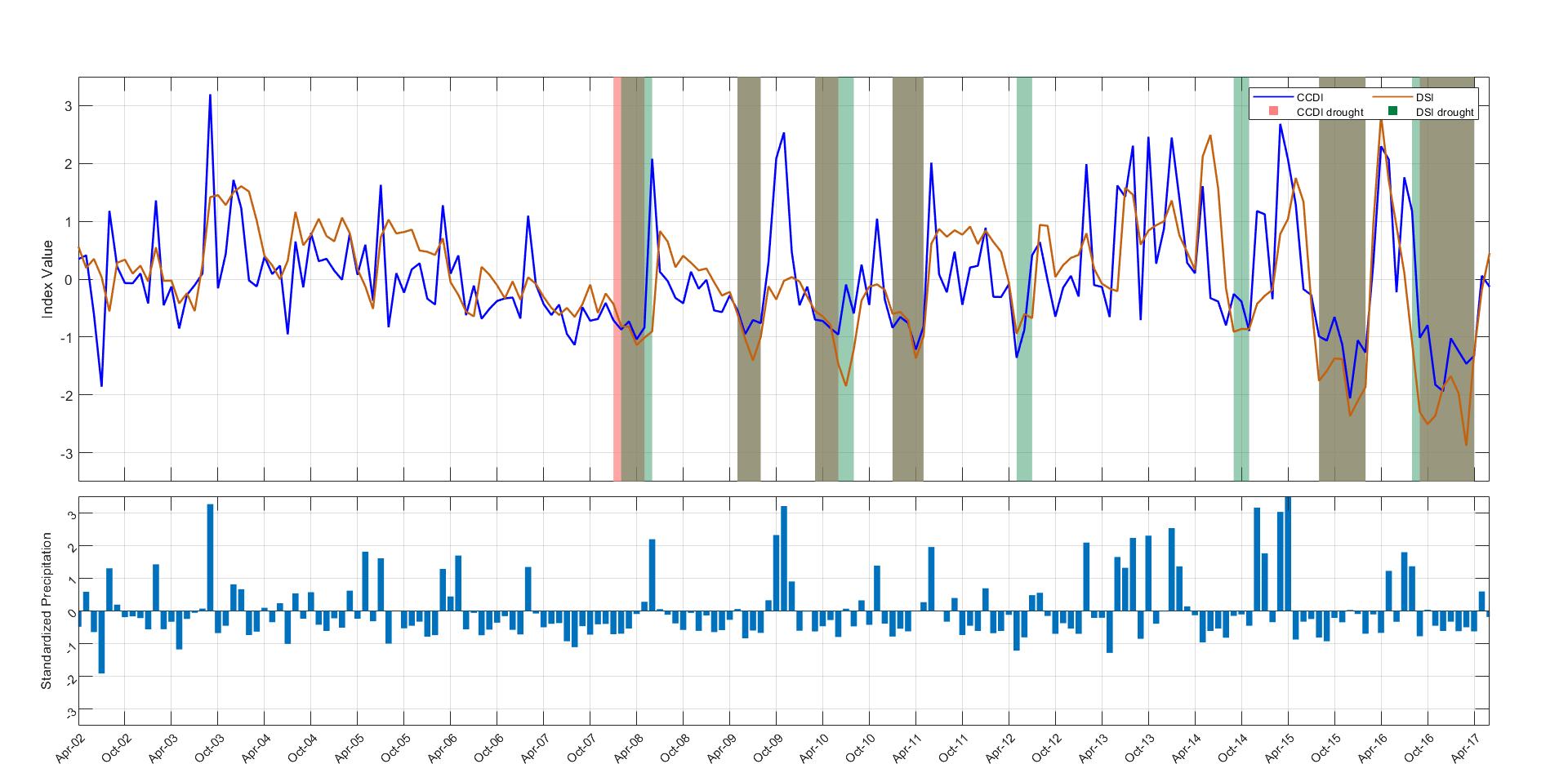


(XIII)

**
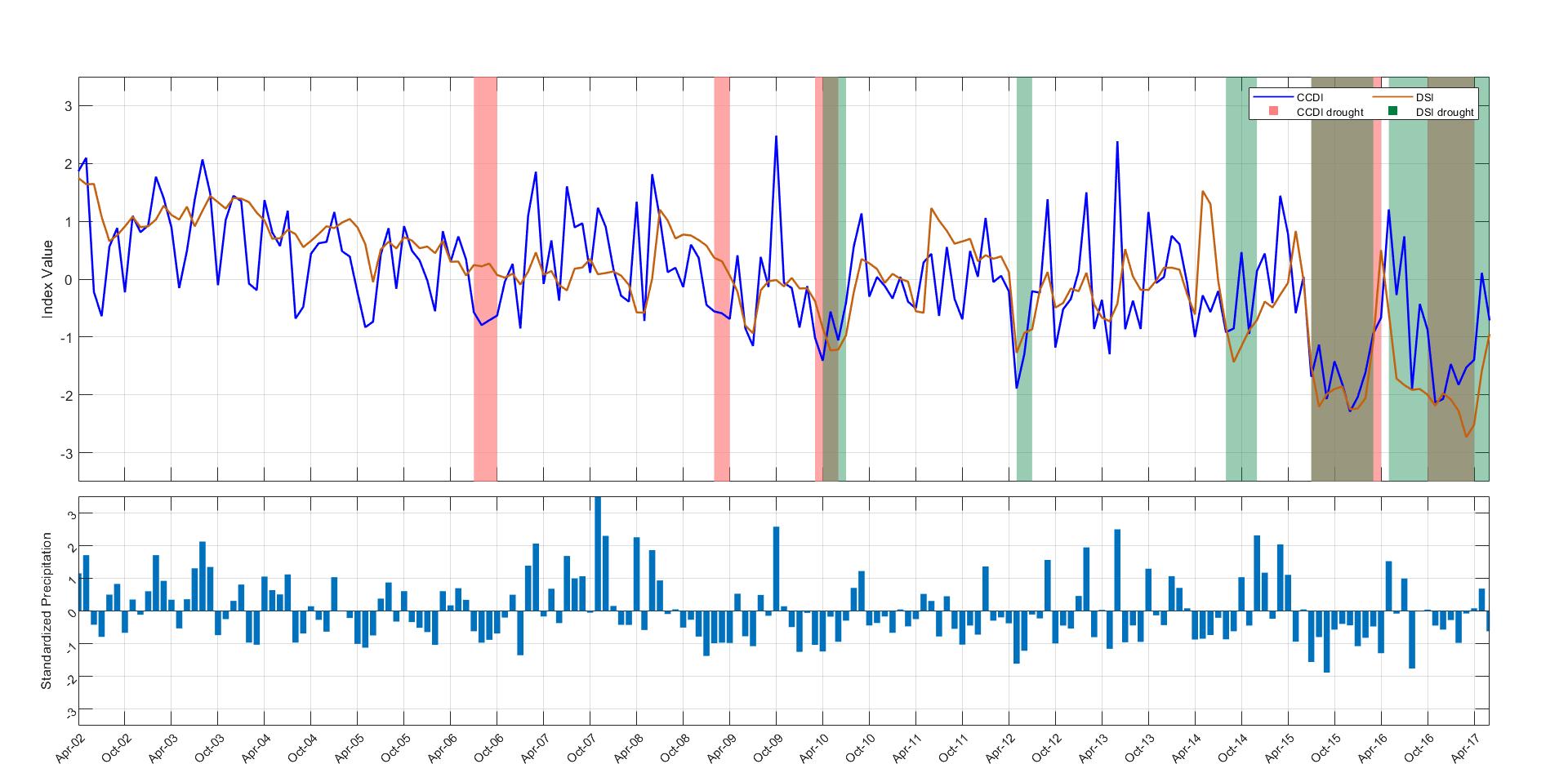
**

(XIV)


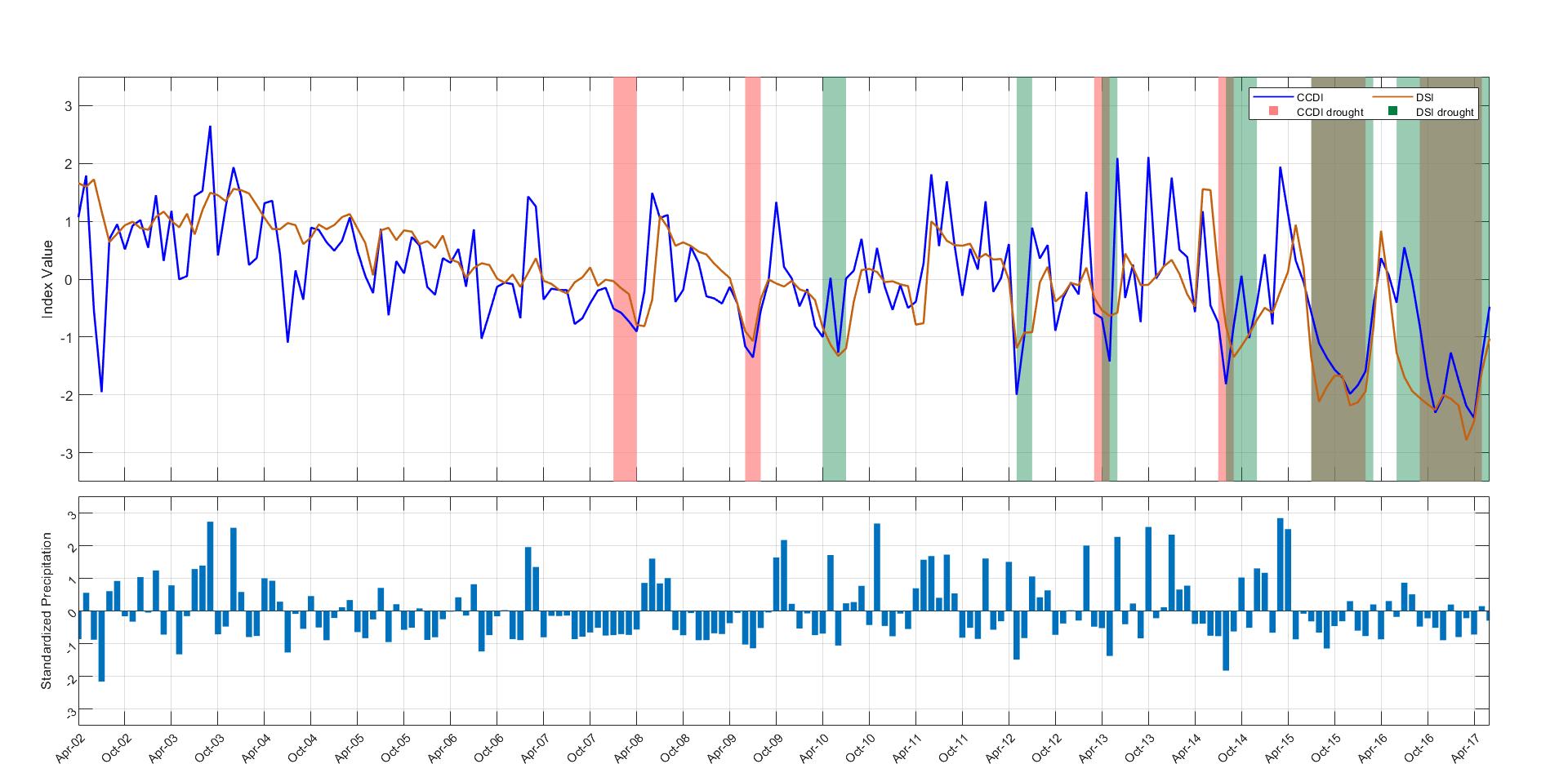


(XV)


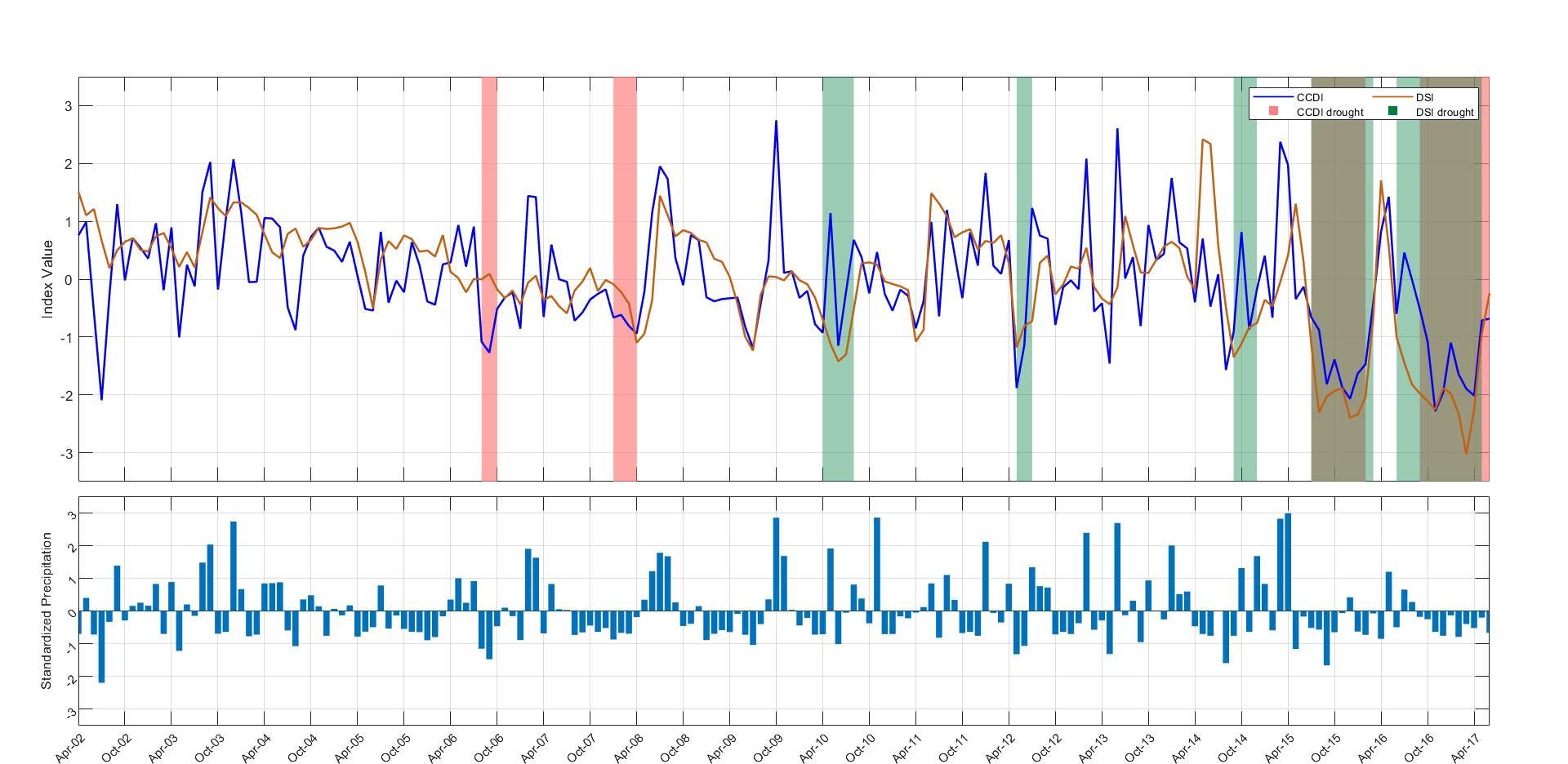


(XVI)


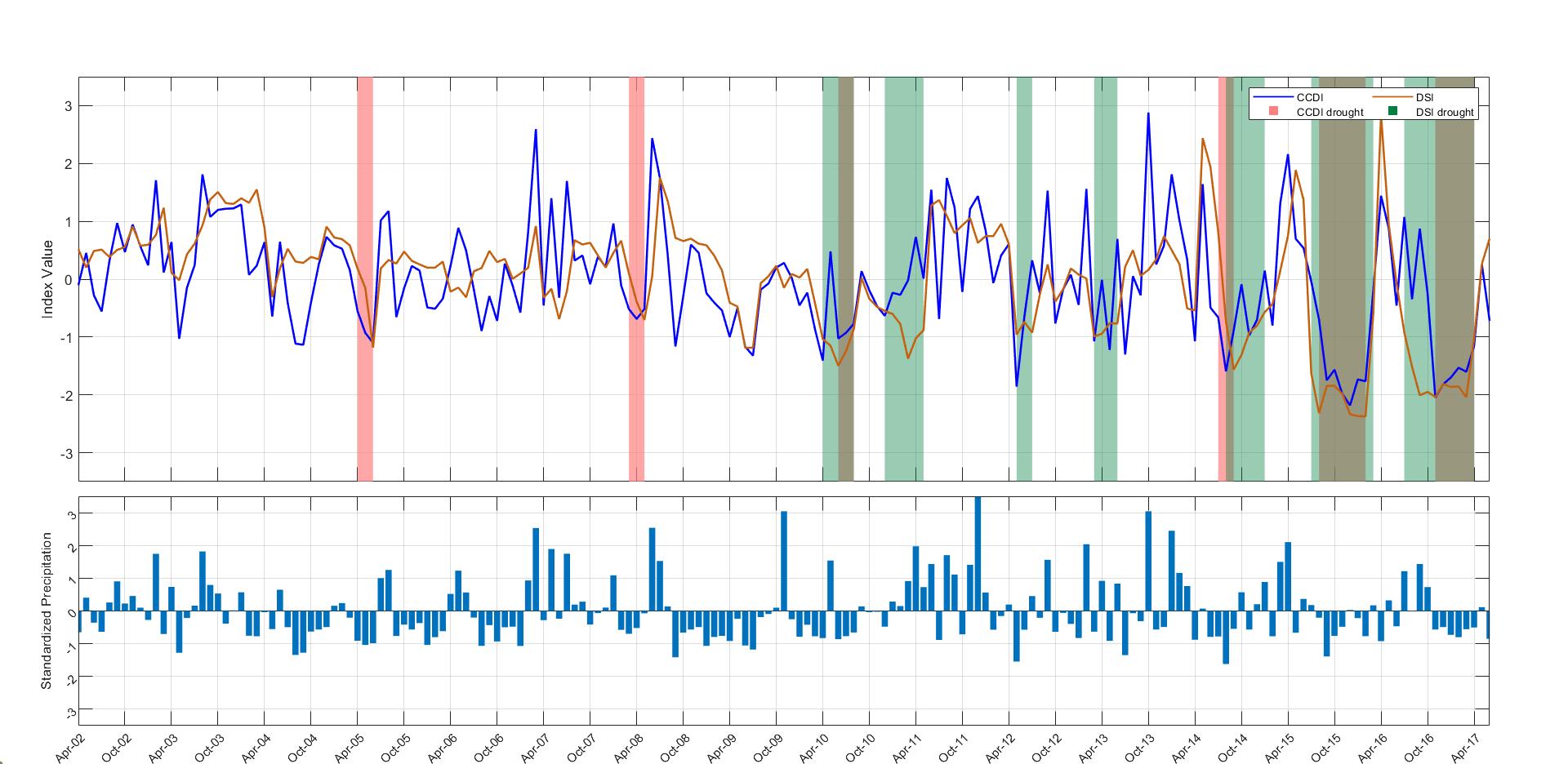


(XVII)

**
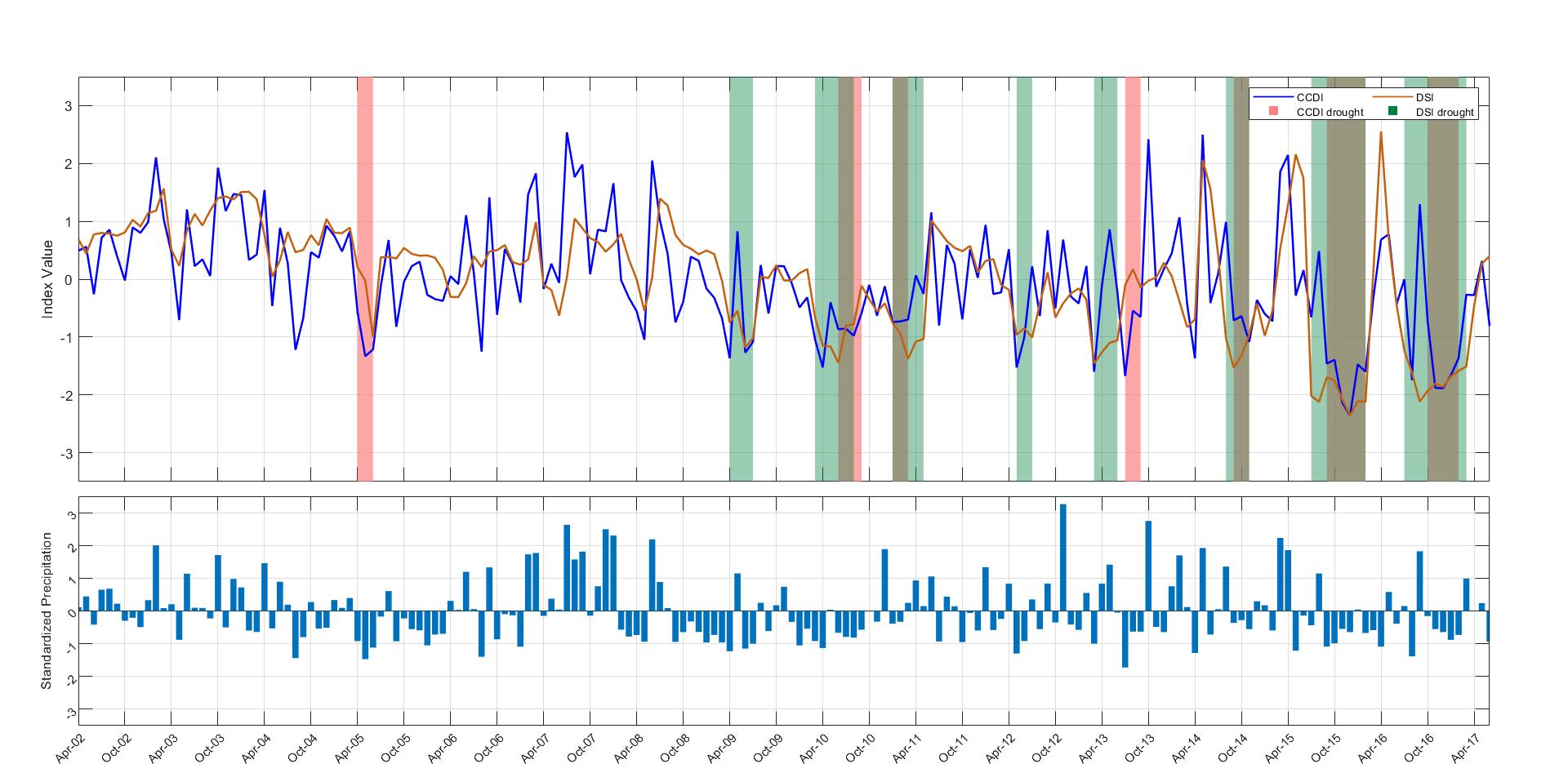
**

(XVIII)

**
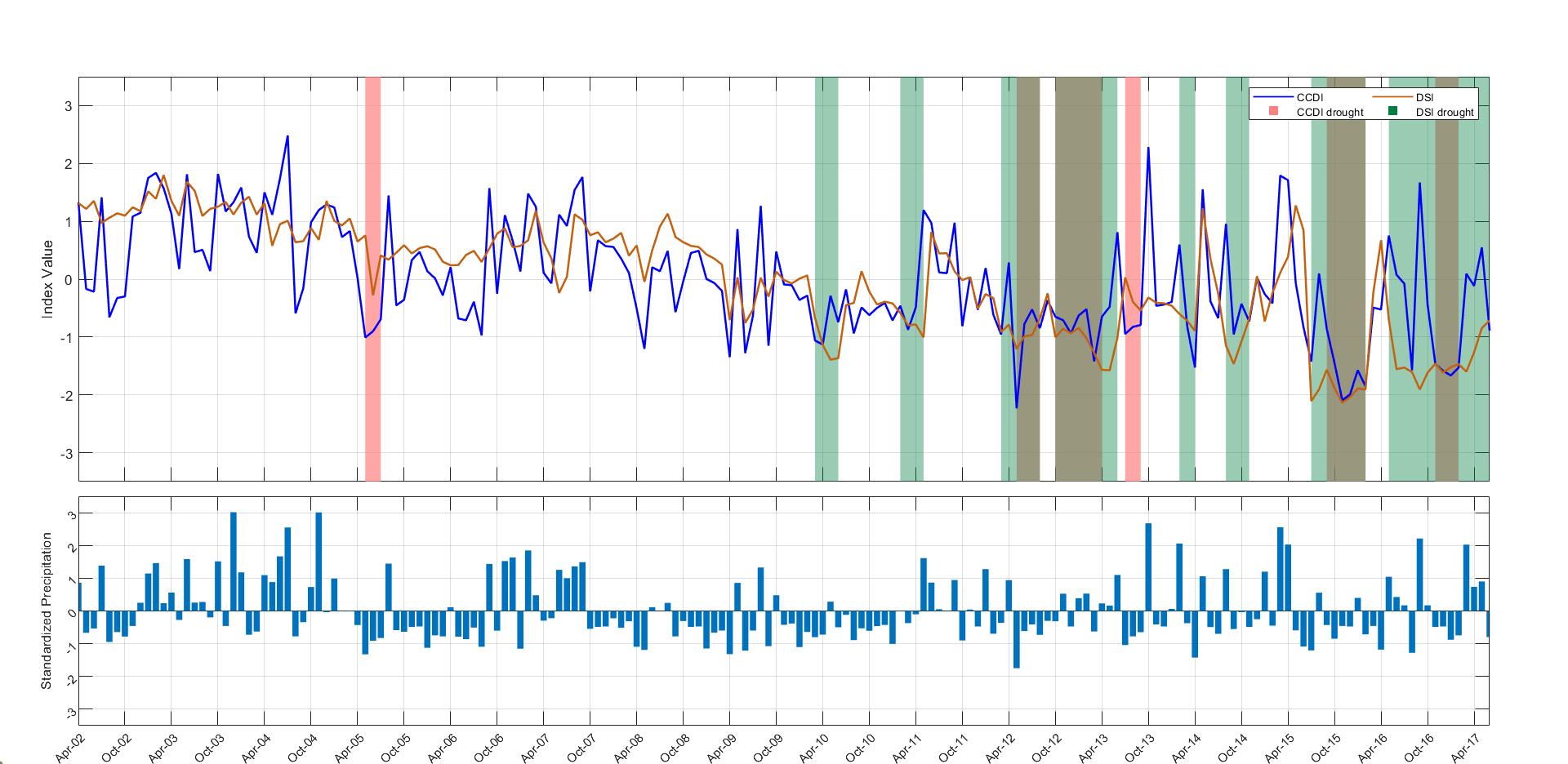
**

(XIX)

**
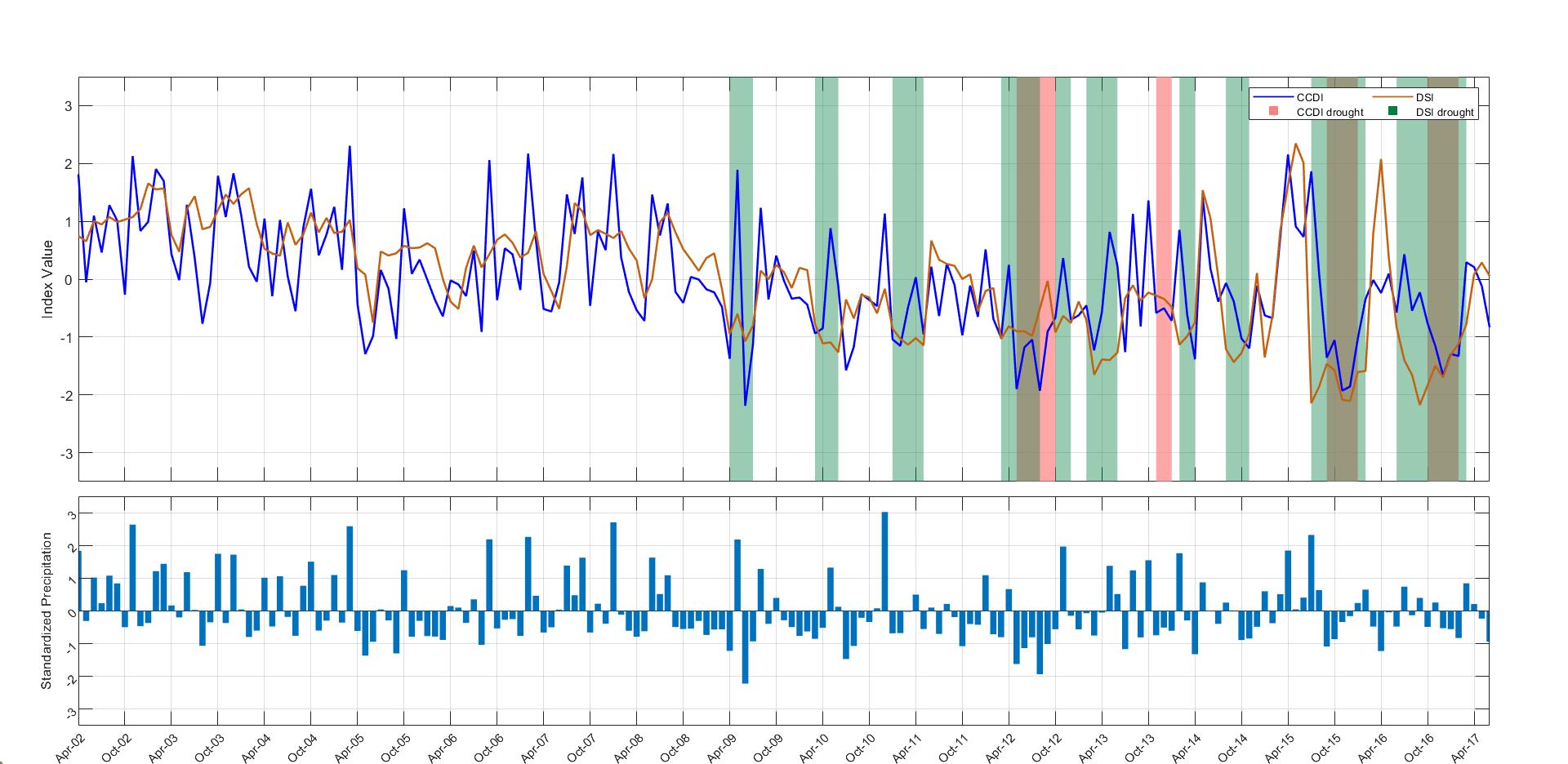
**

(XX)


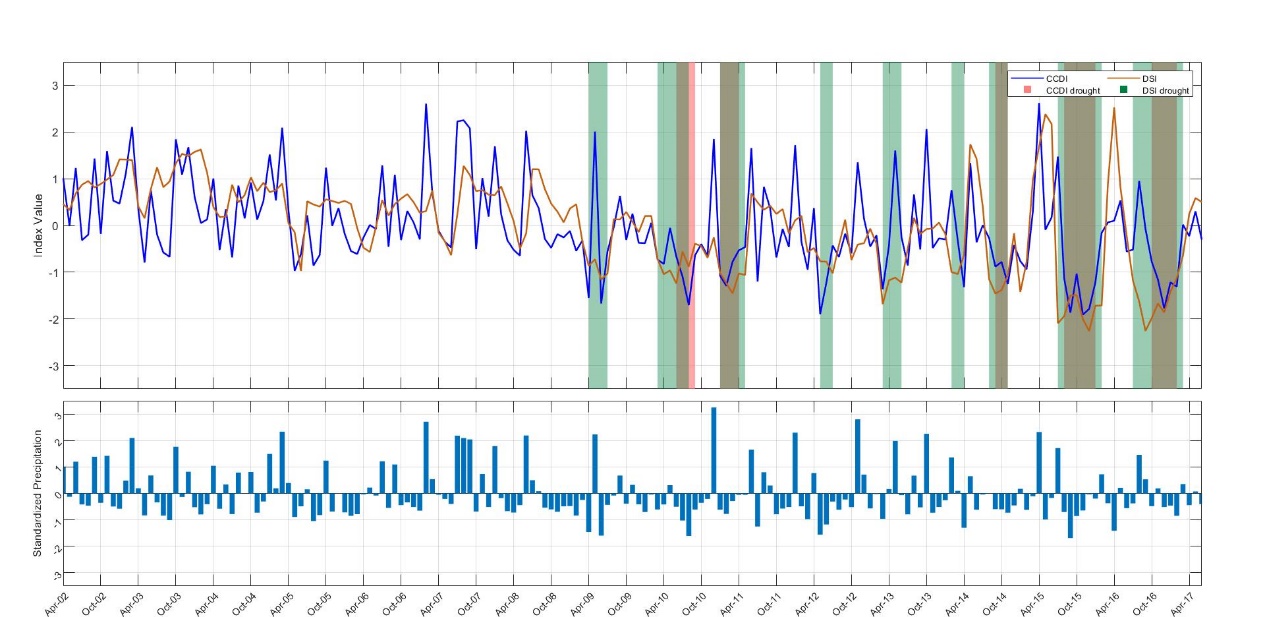


(XXI)

**
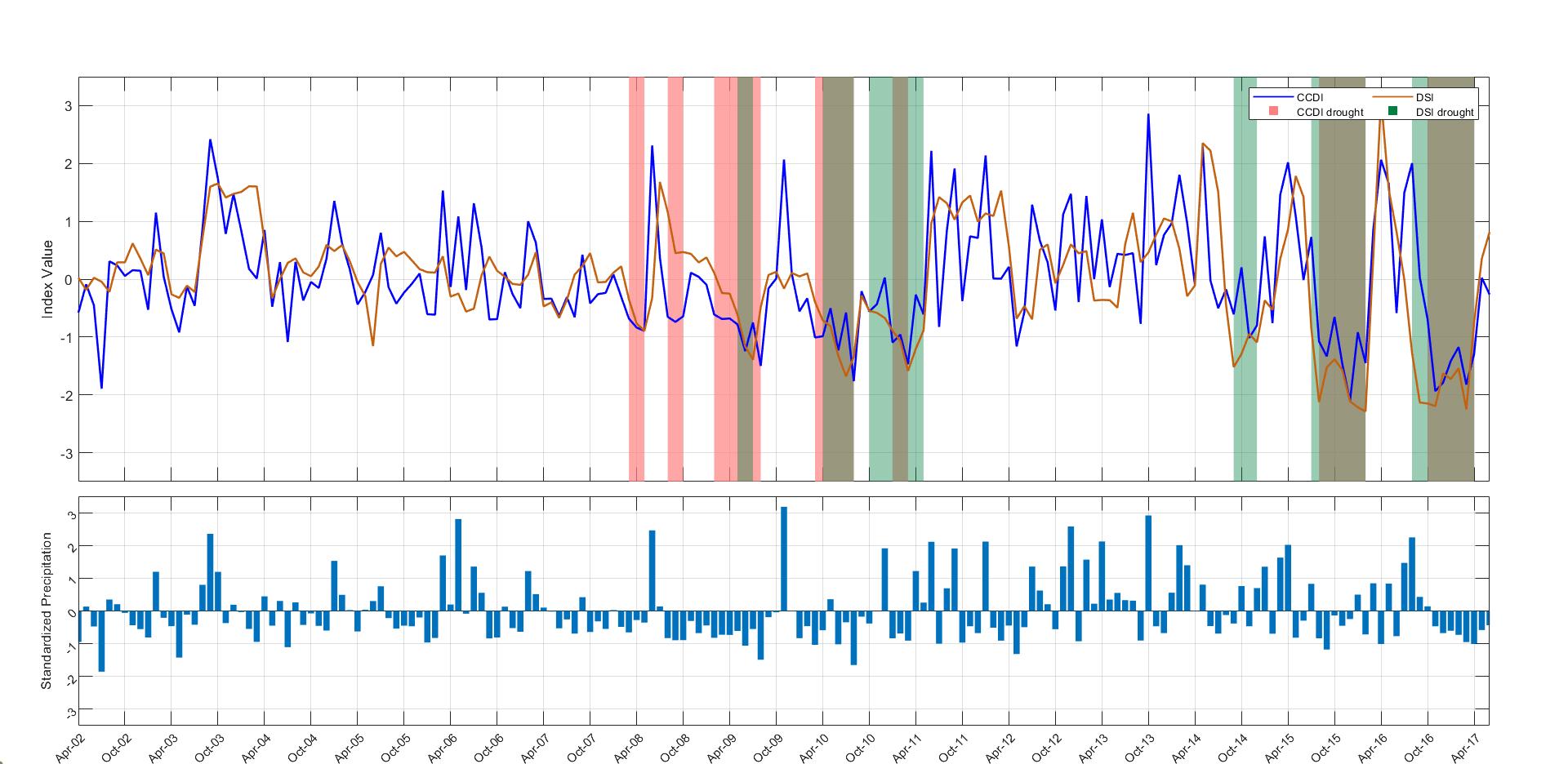
**

(XXII)

**
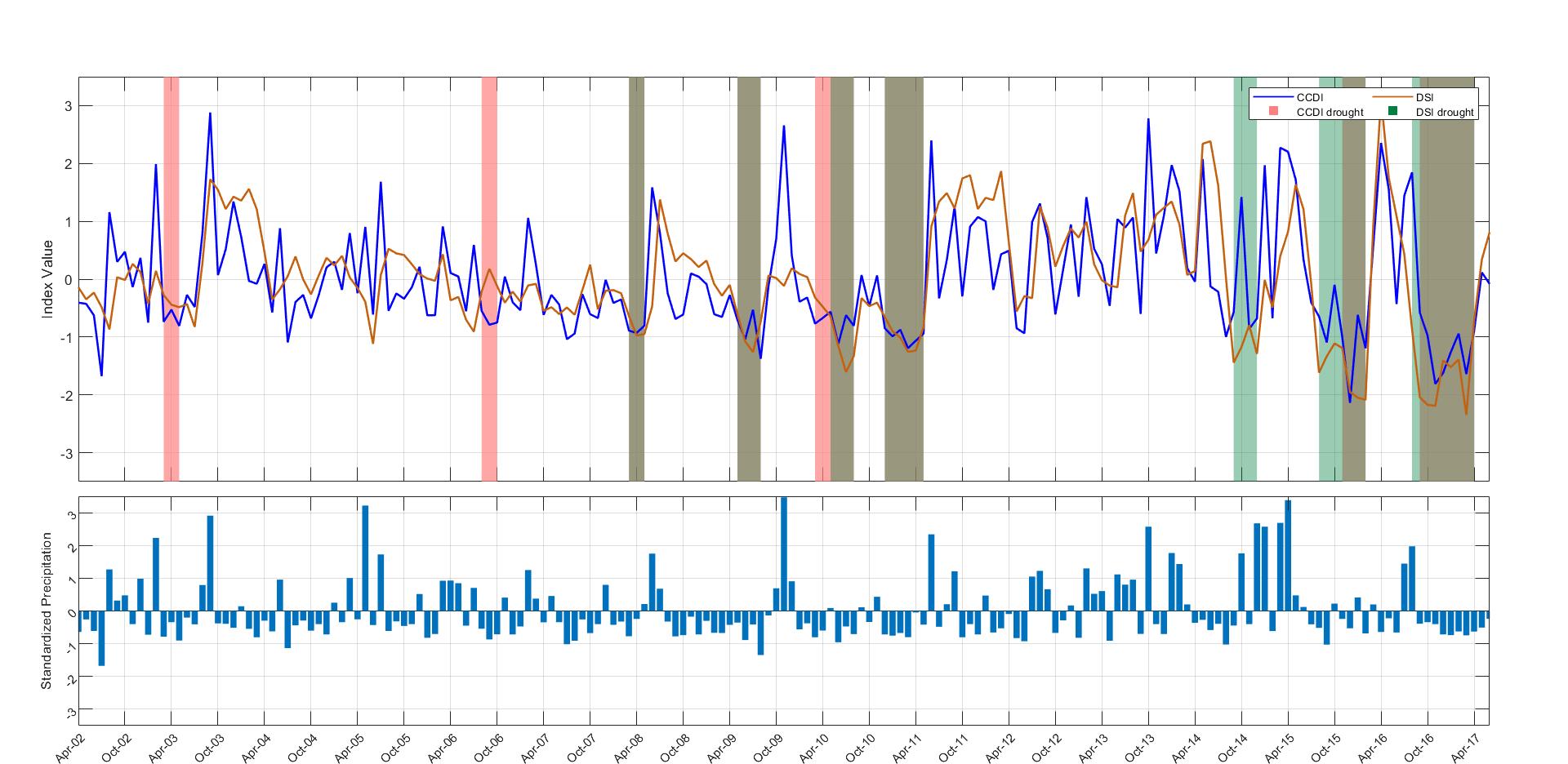
**

(XXIII)

**
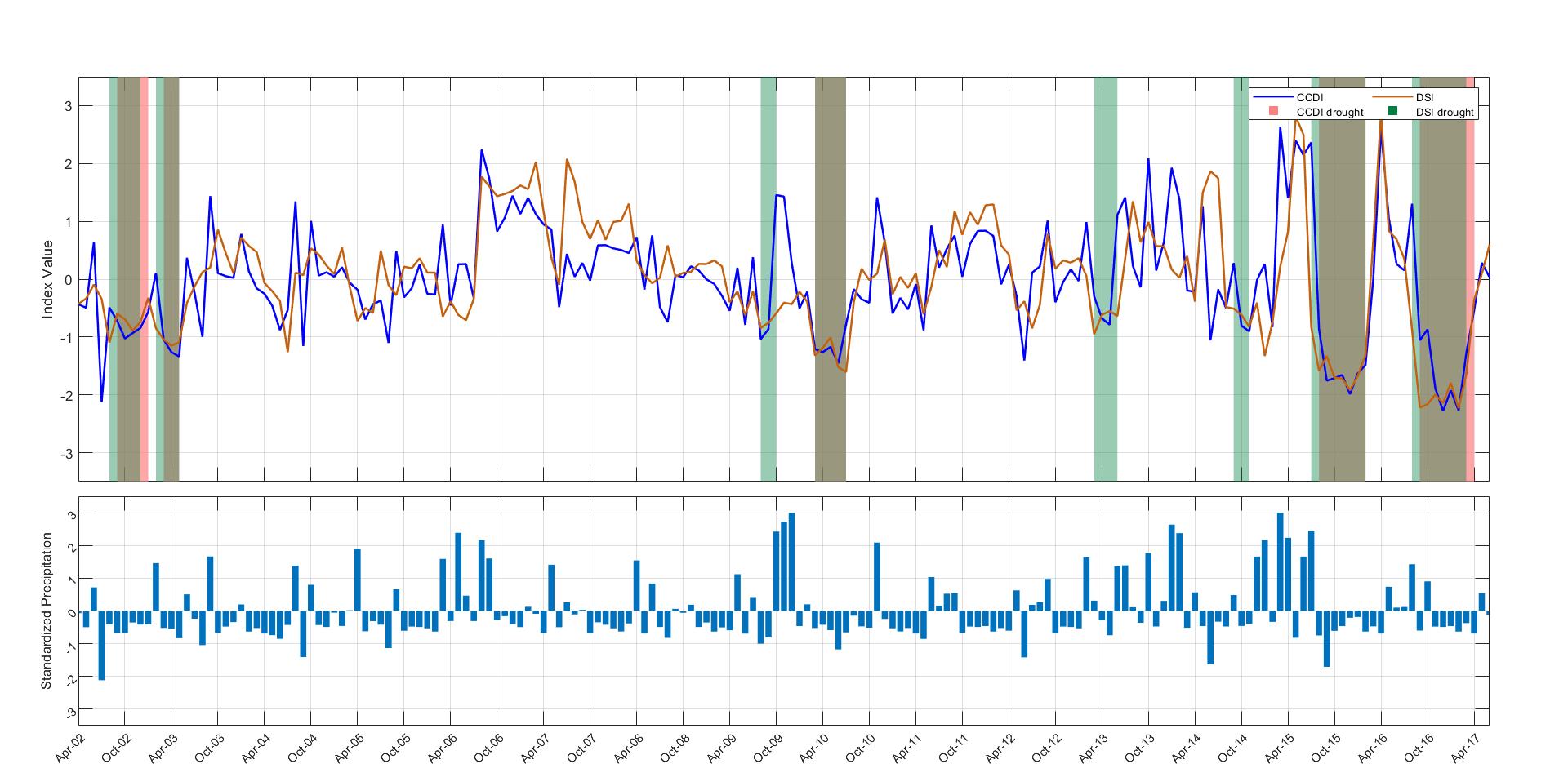
**

(XXIV)

**
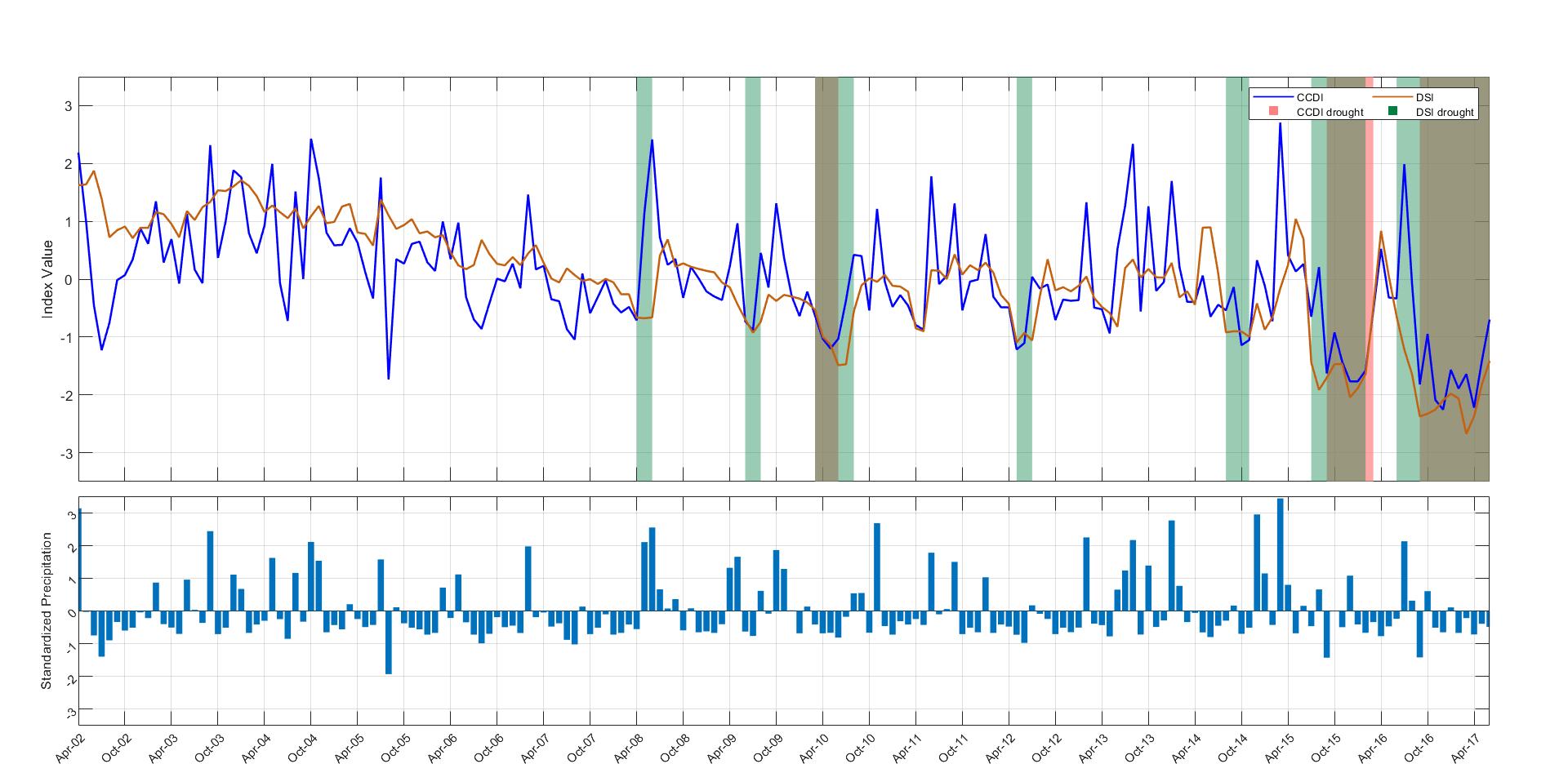
**

(XXV)

**
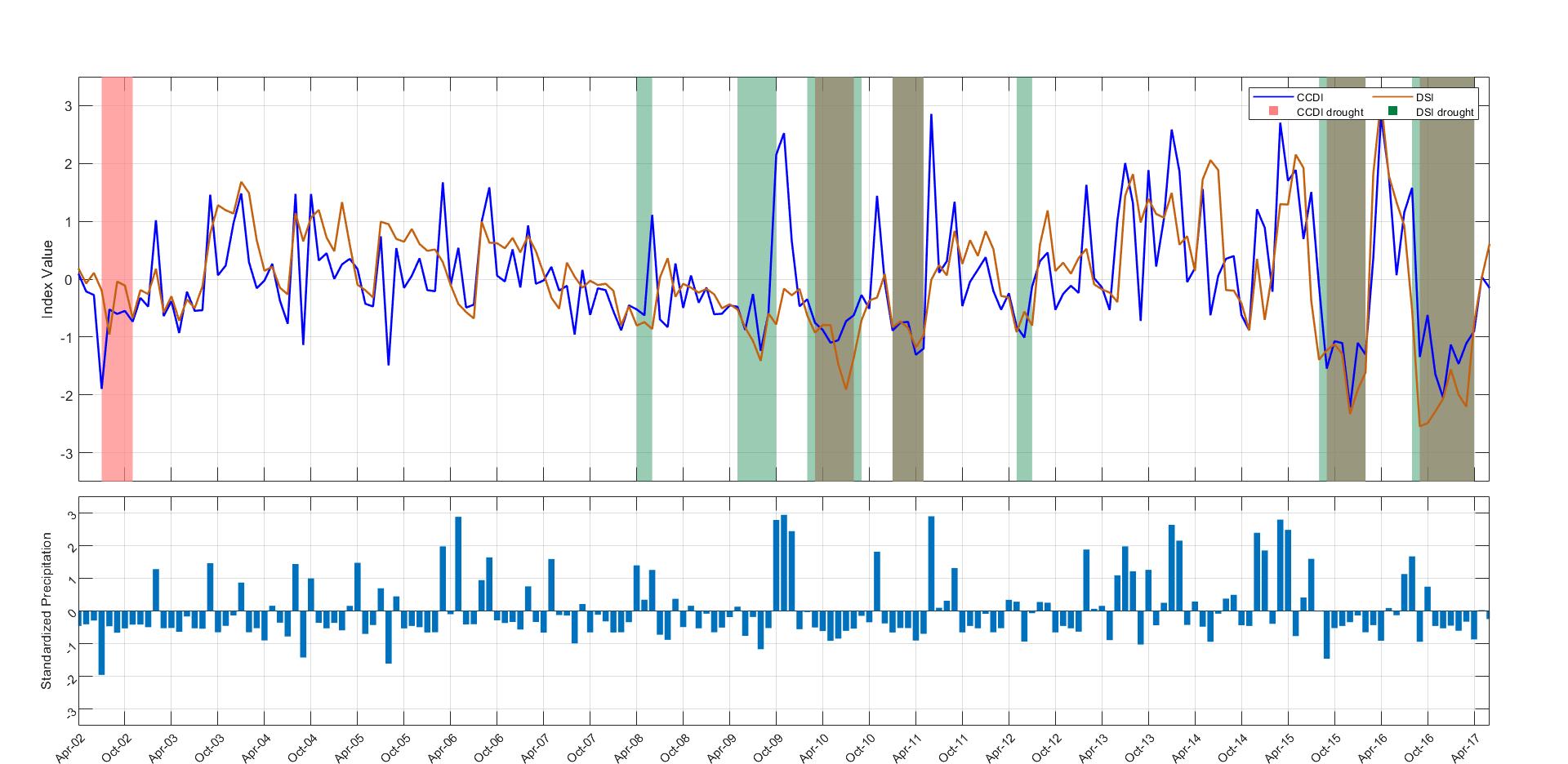
**

(XXVI)

**
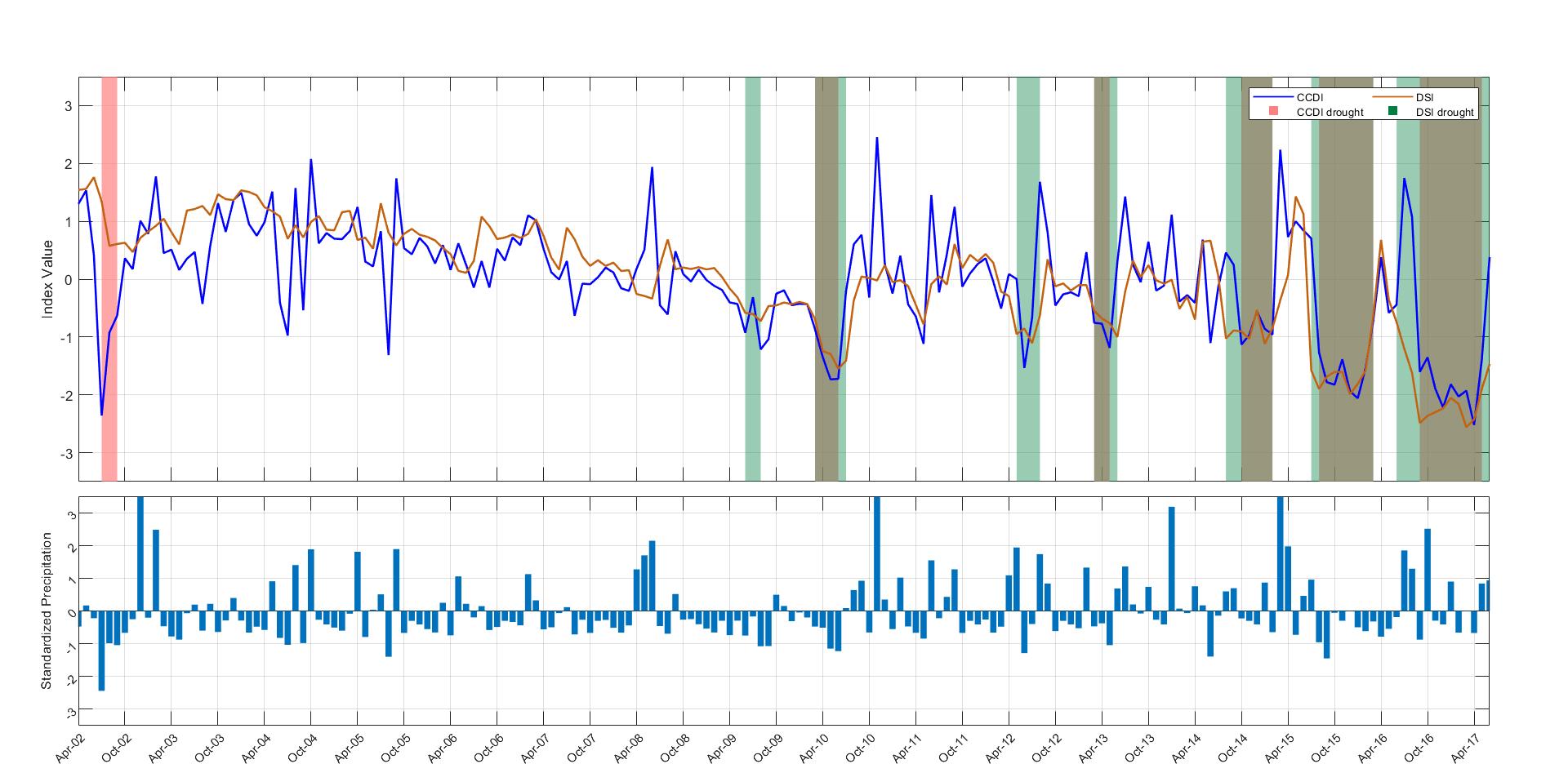
**

(XXVII)

**
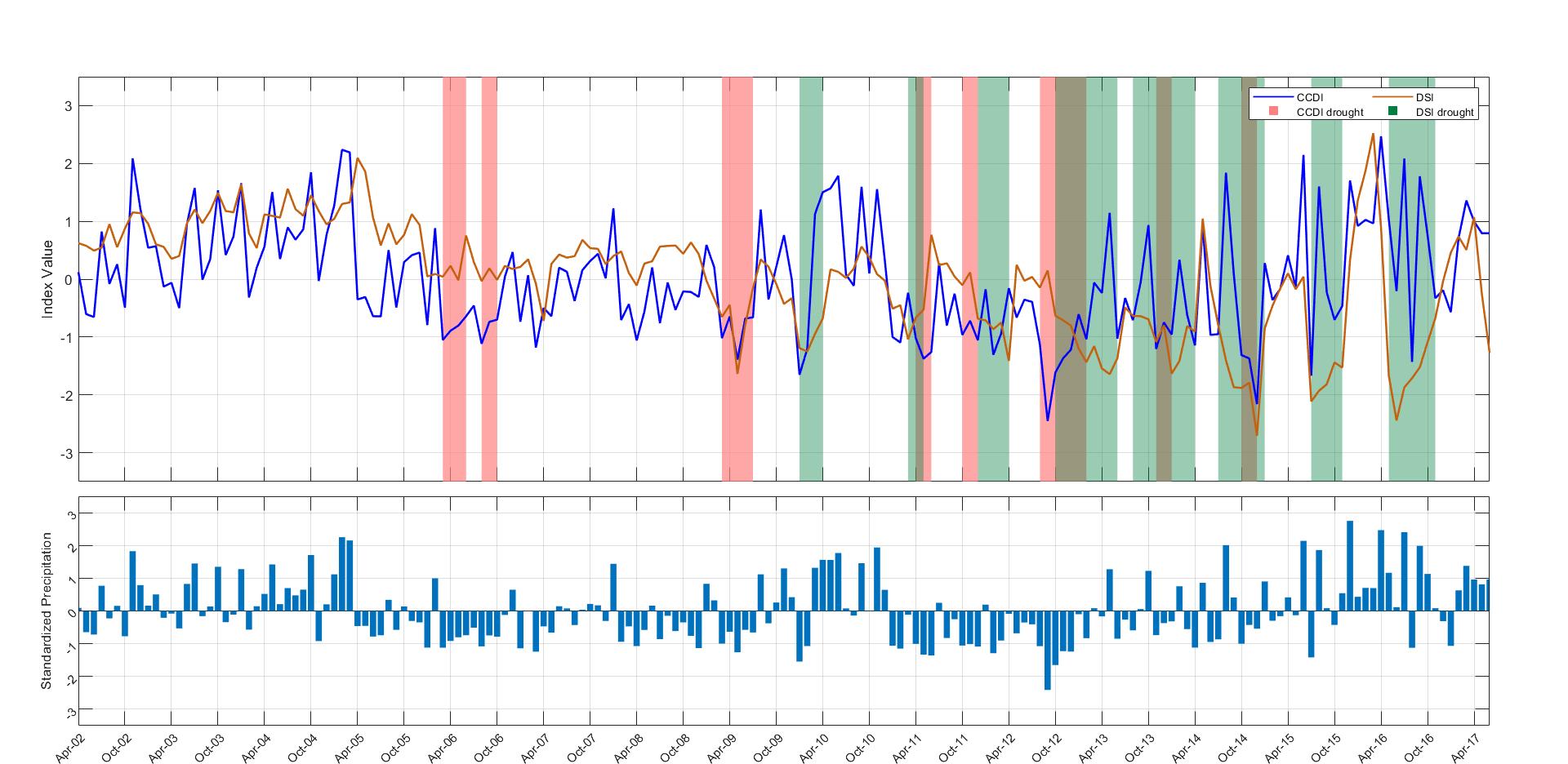
**

(XXVIII)

**
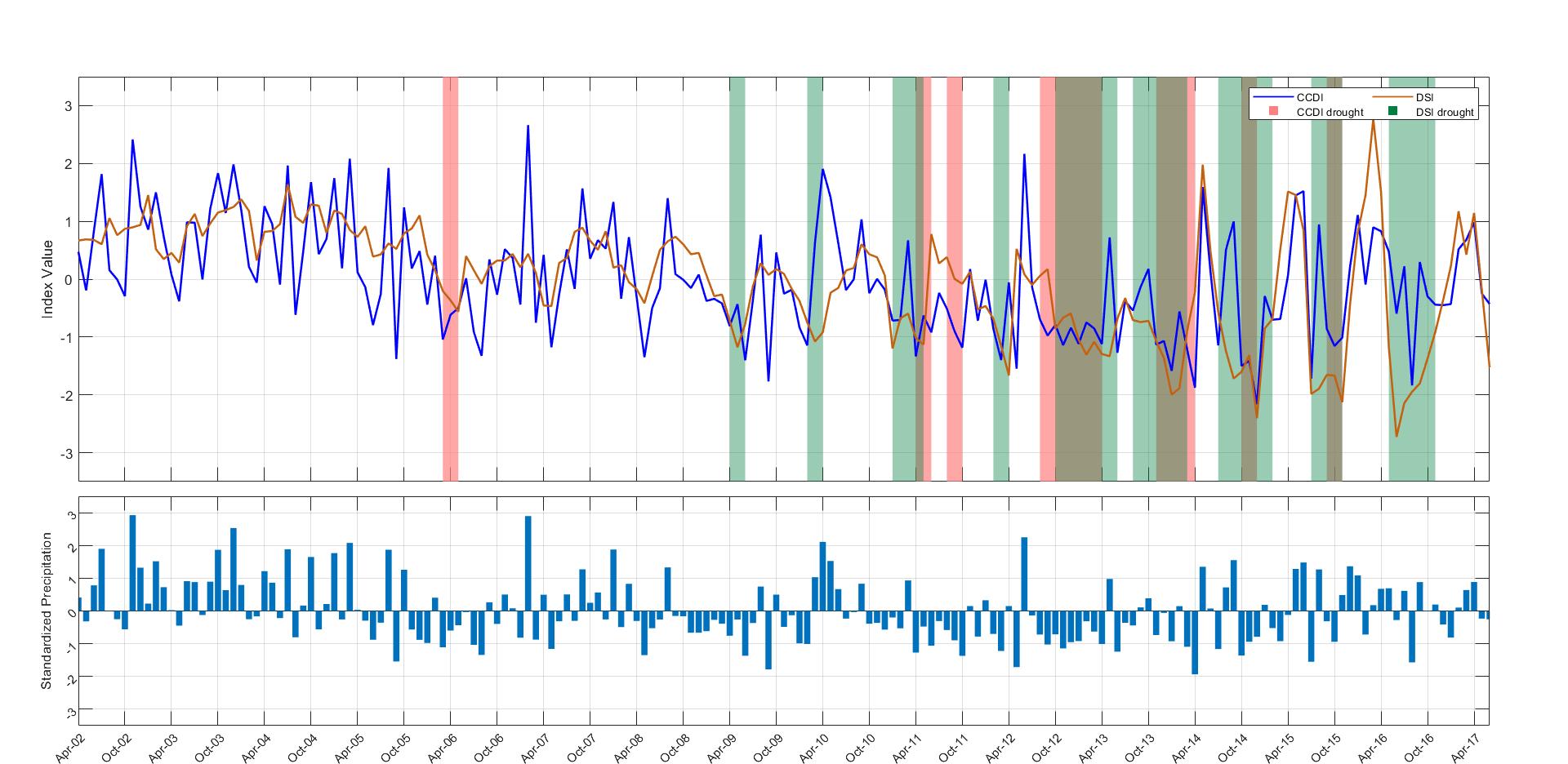
**

(XXIX)

**
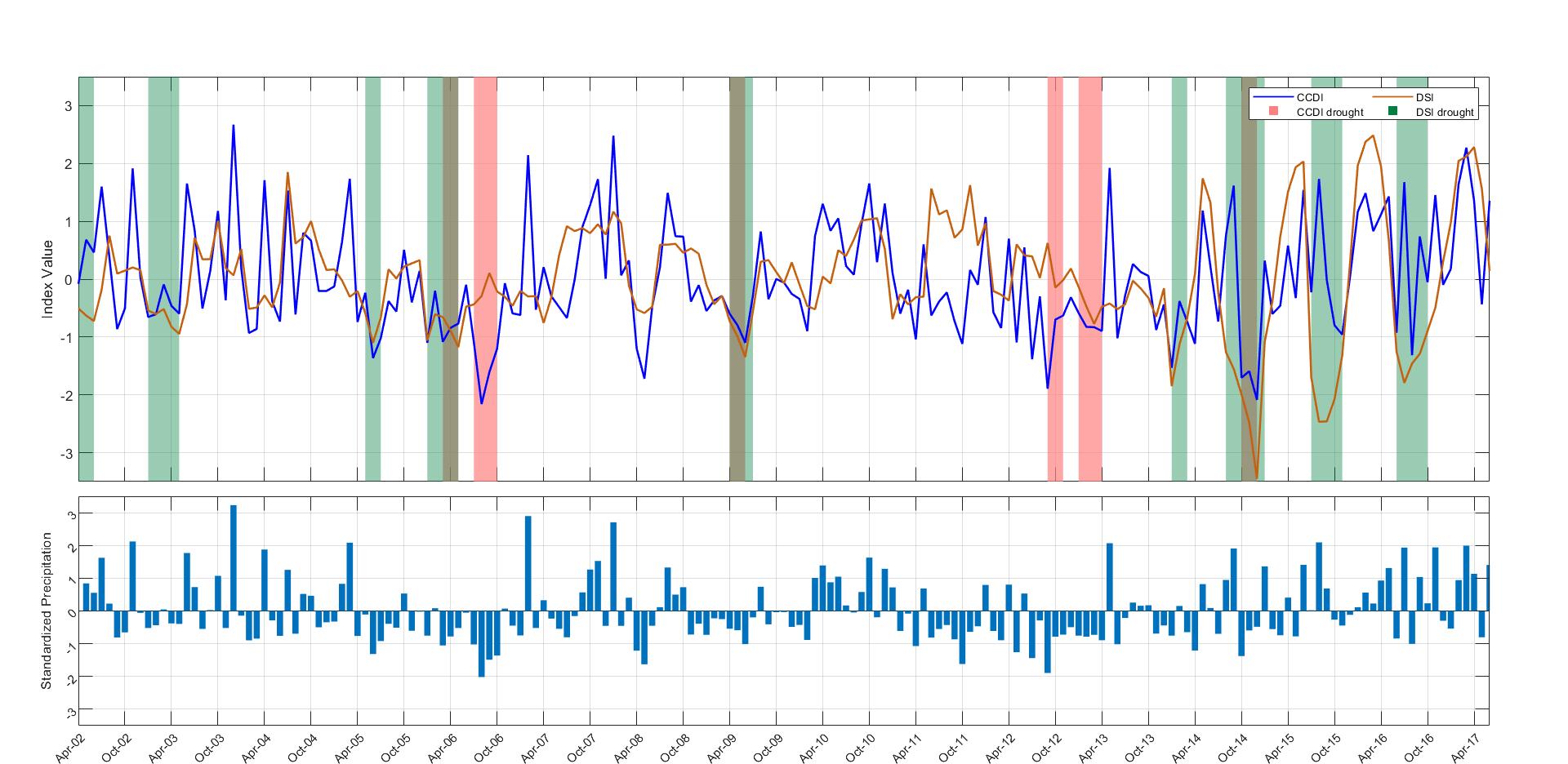
**

(XXX)

**
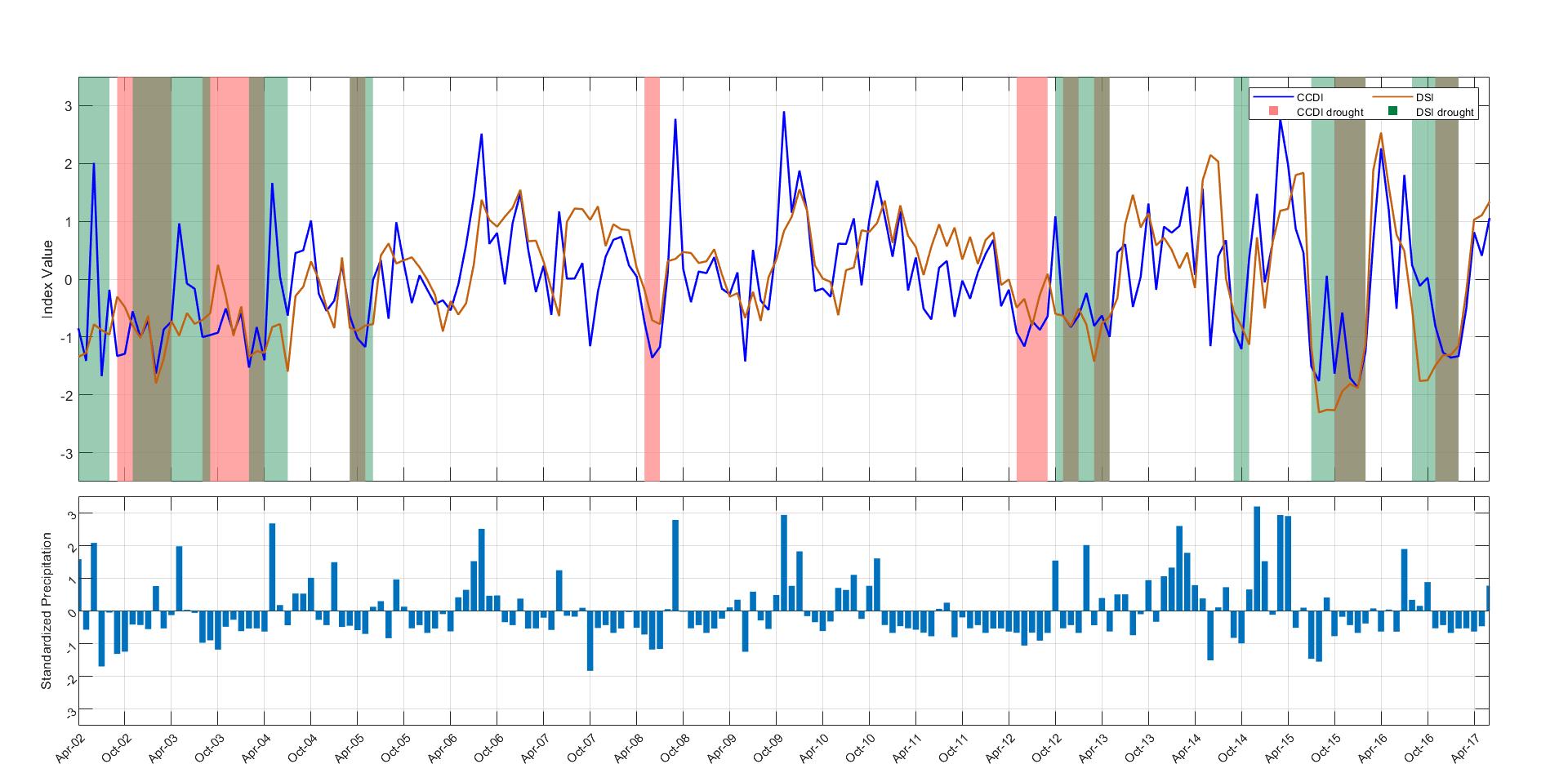
**

(XXXI)

**
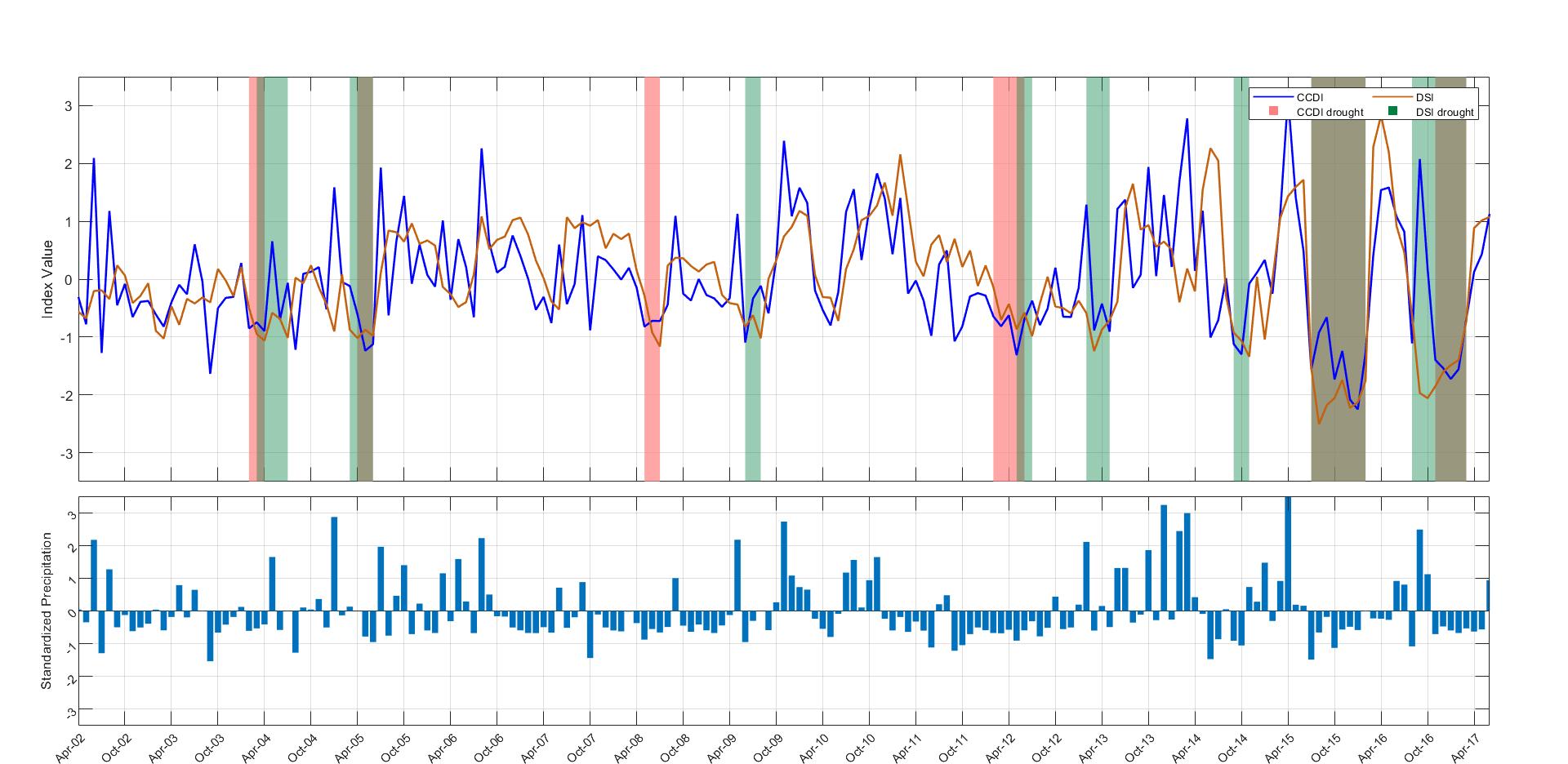
**

(XXXII)

**
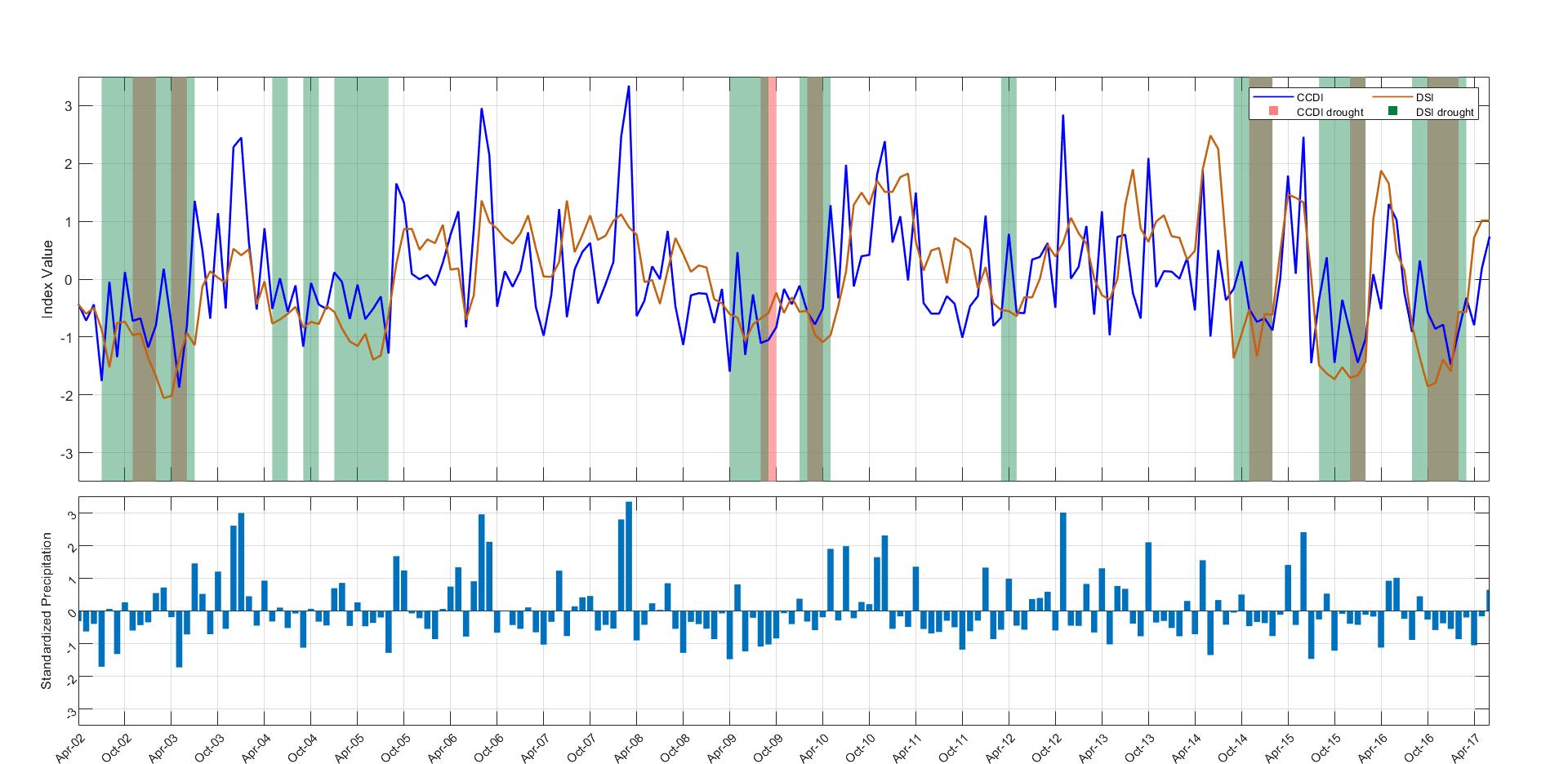
**

(XXXIII)

**
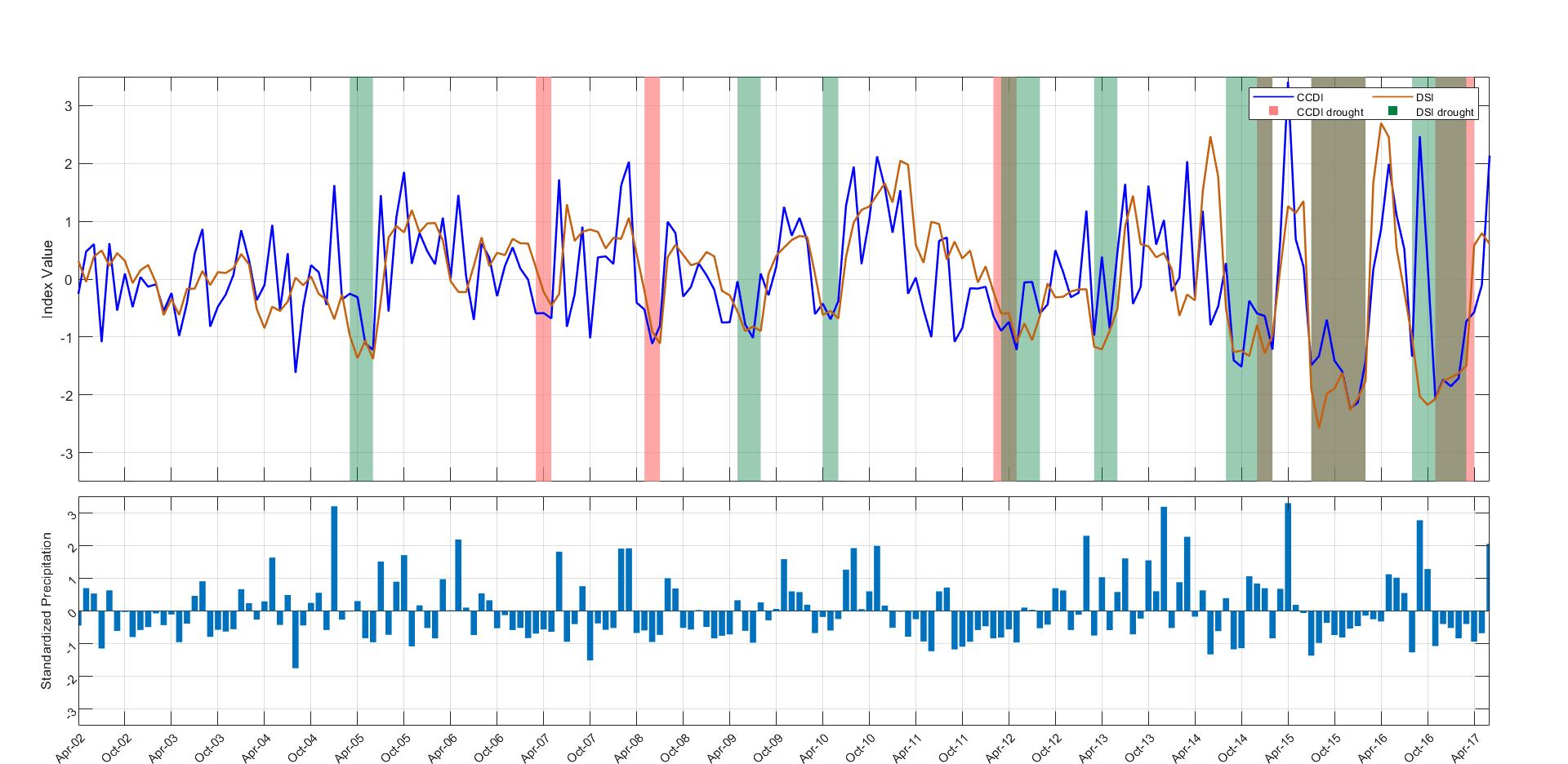
**

(XXXIV)

**
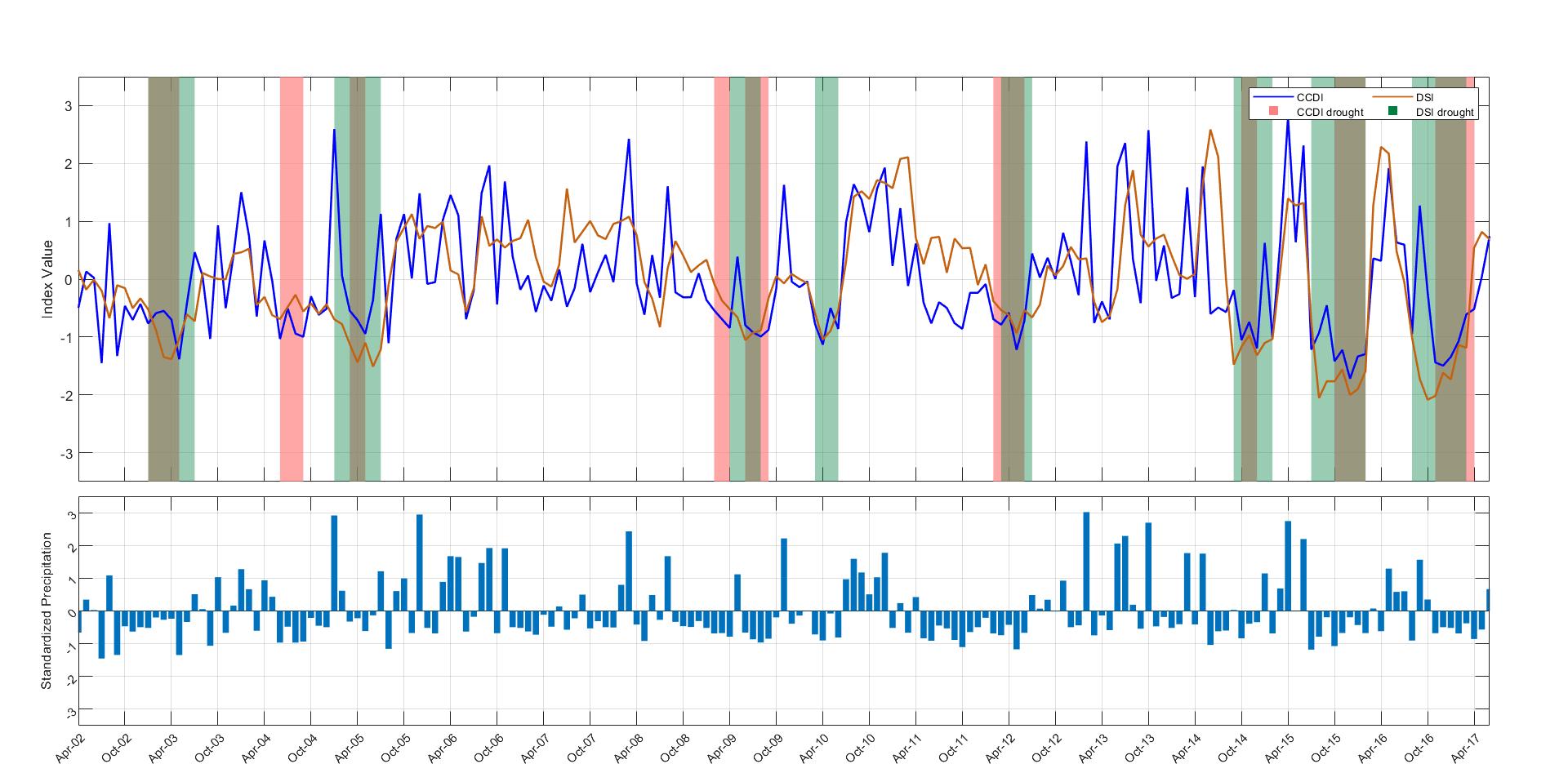
**

(XXXV)

**
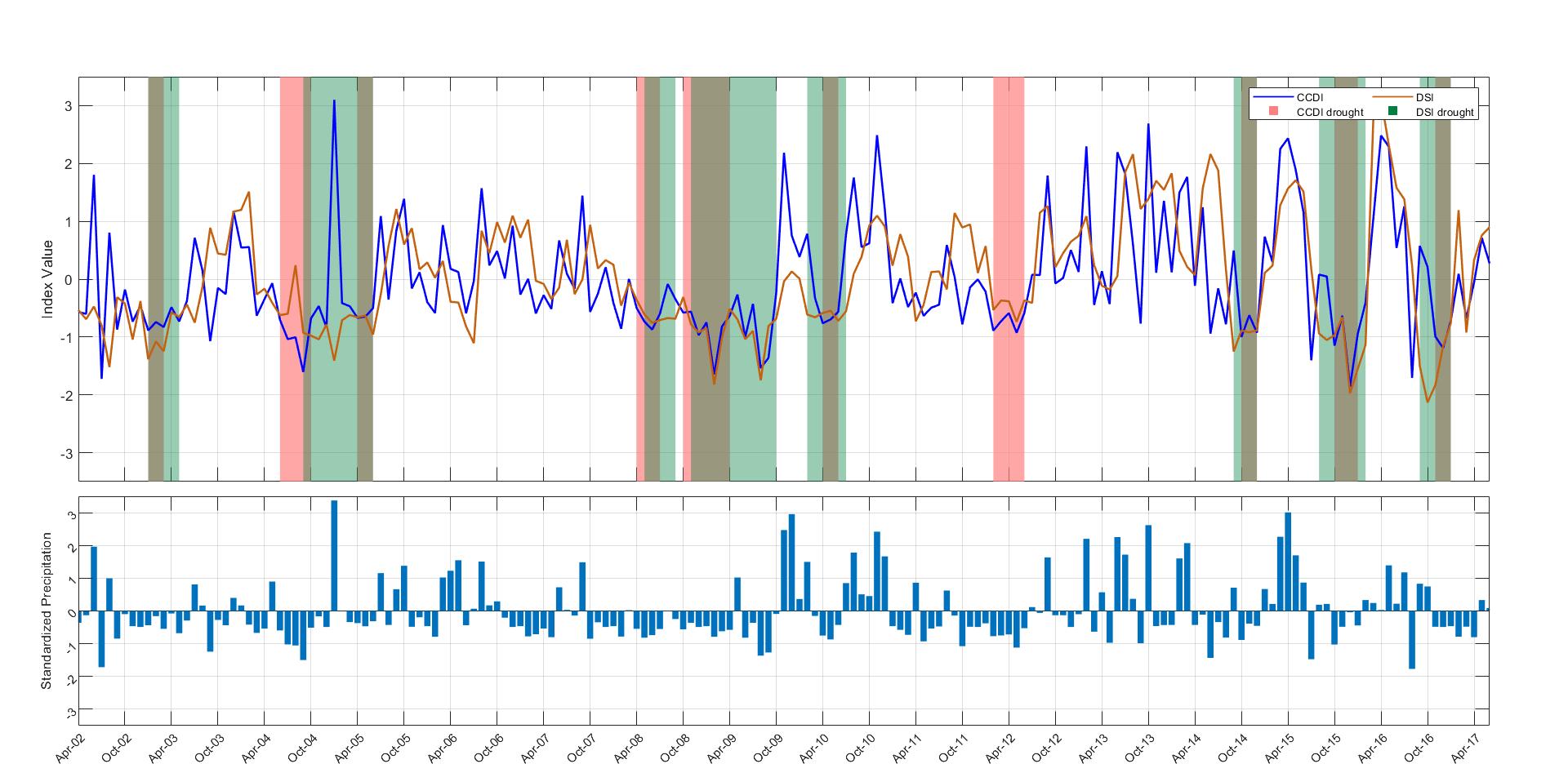
**

(XXXVI)

**
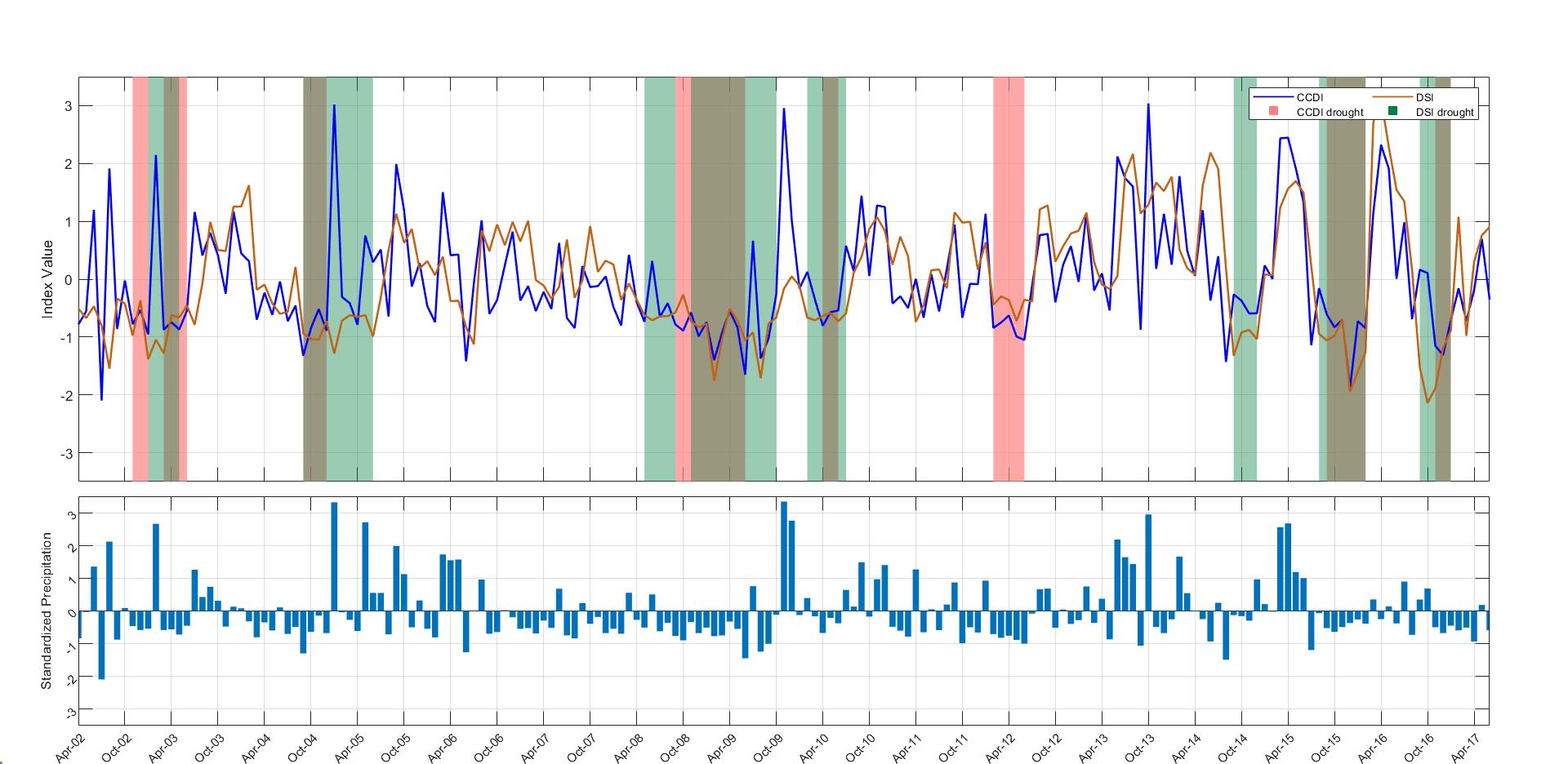
**

(XXXVII)

**
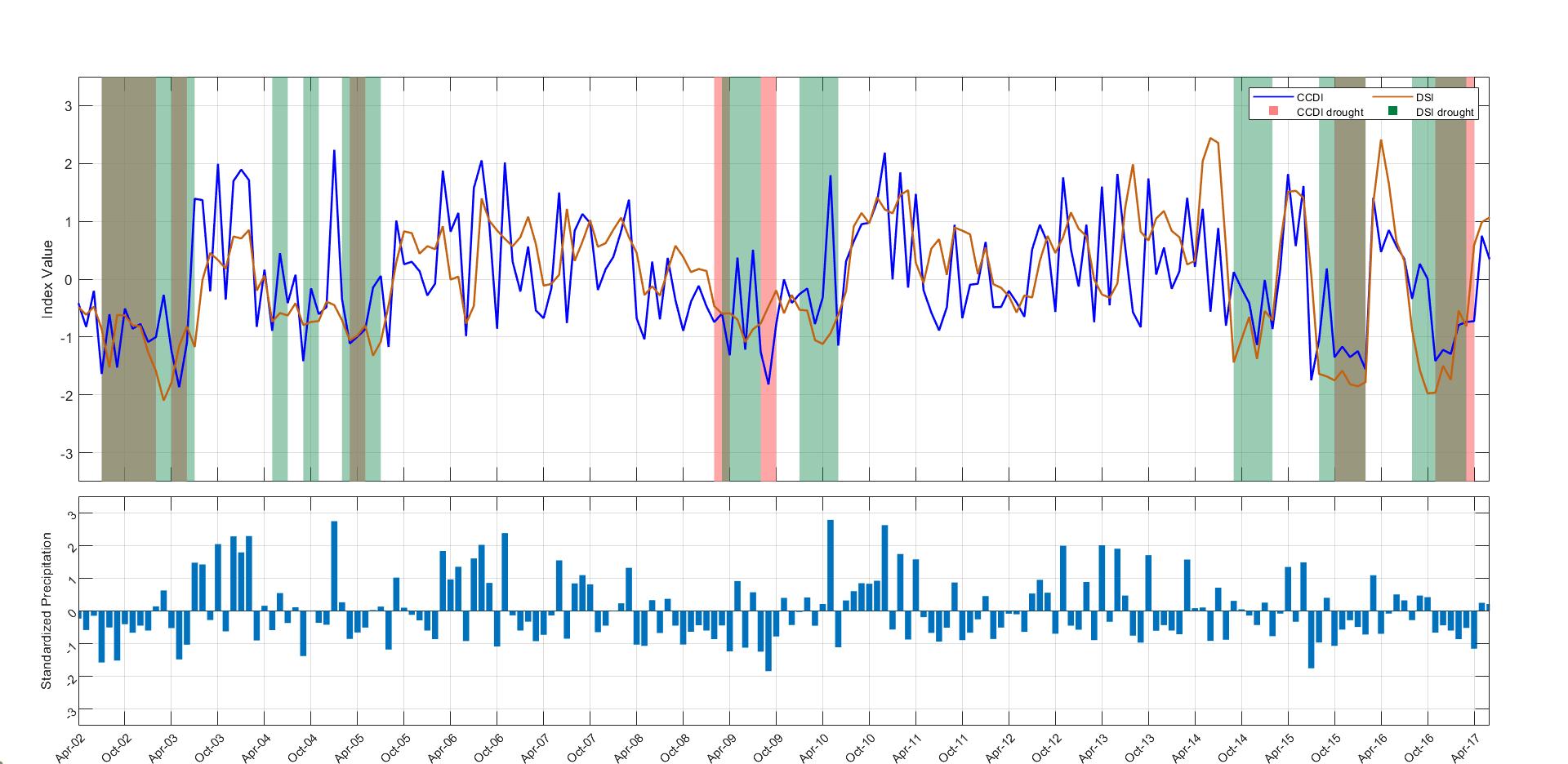
**

(XXXVIII)

**
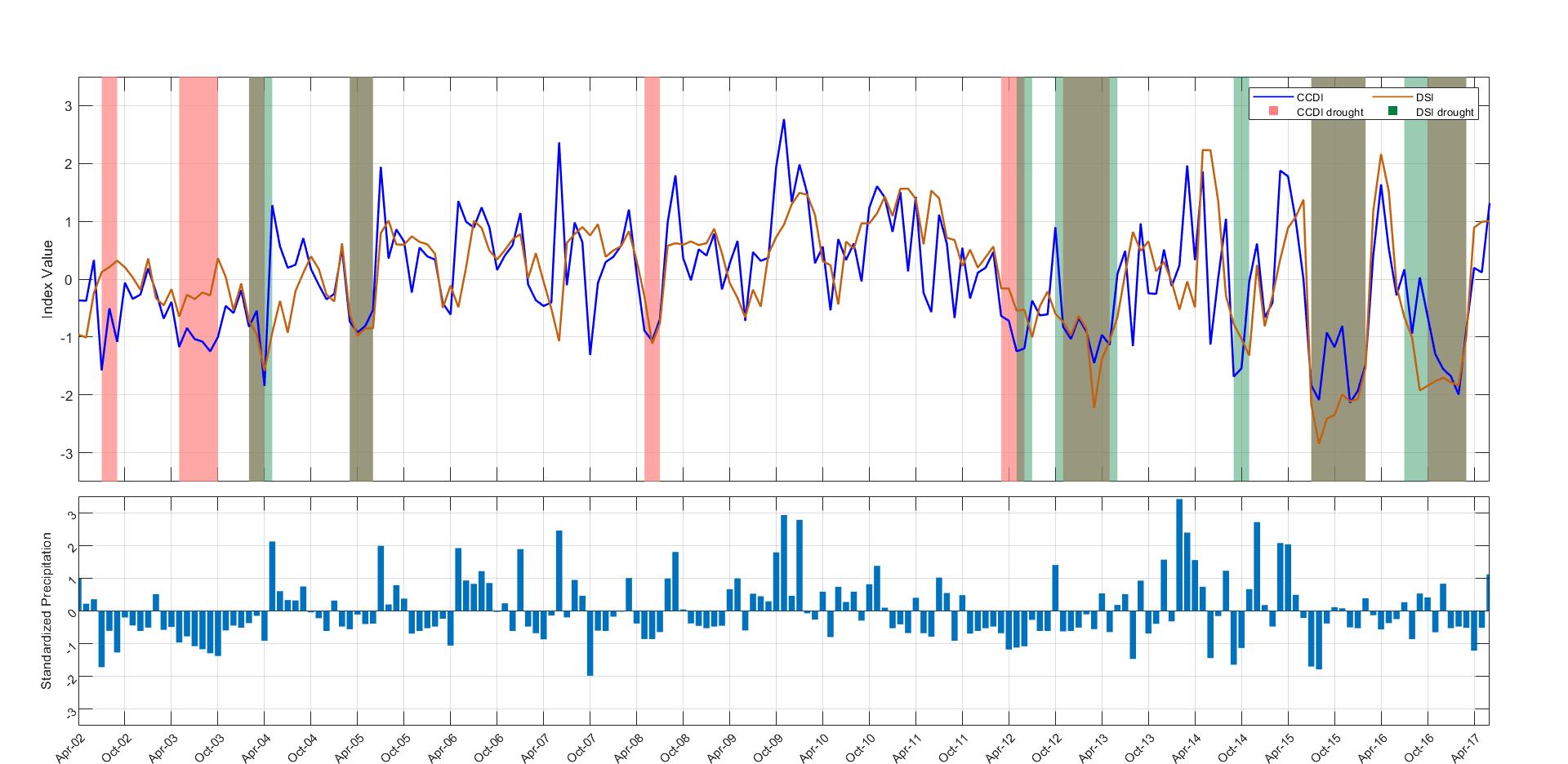
**

(XXXIX)

**
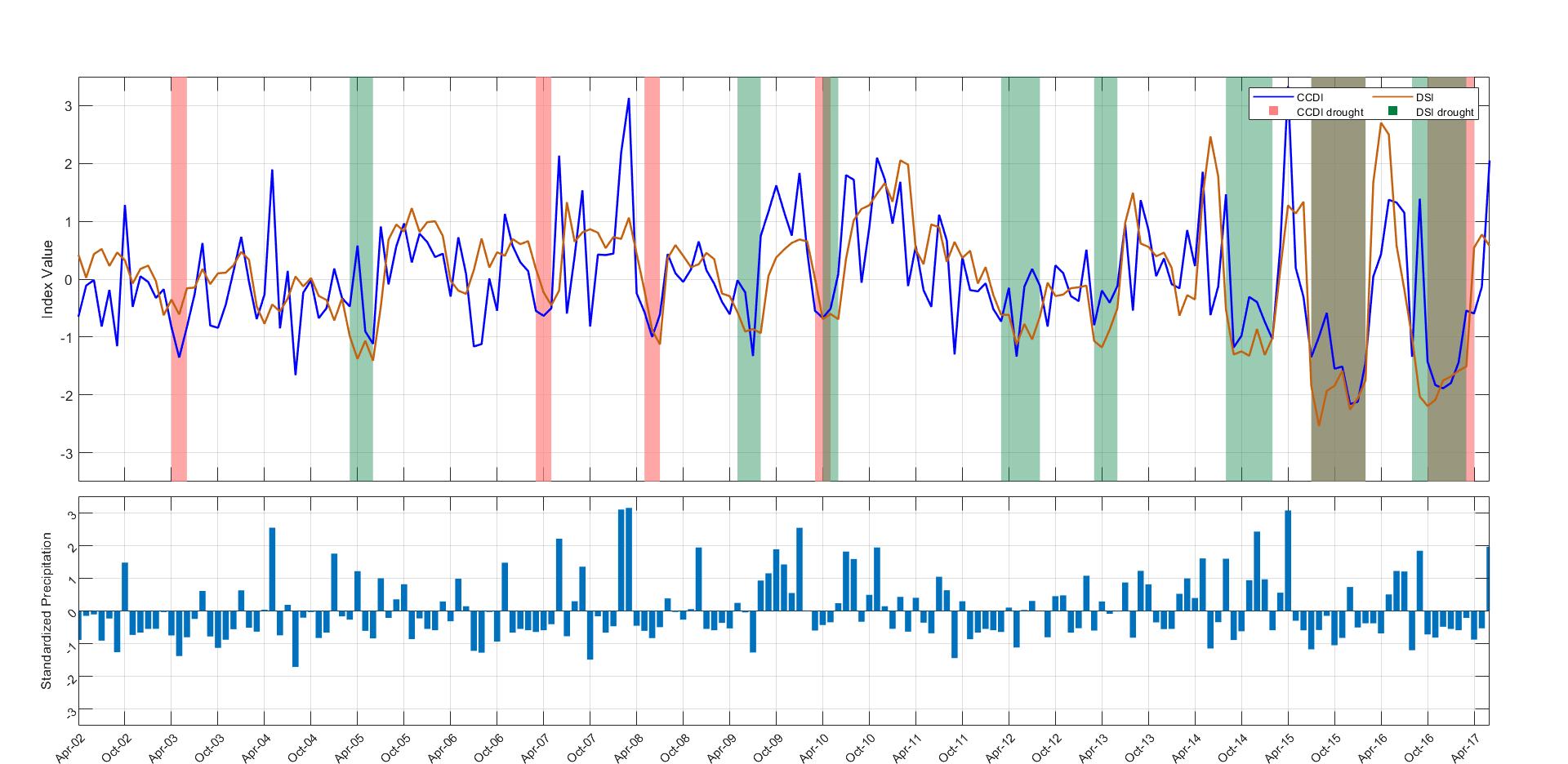
**

(XL)

**
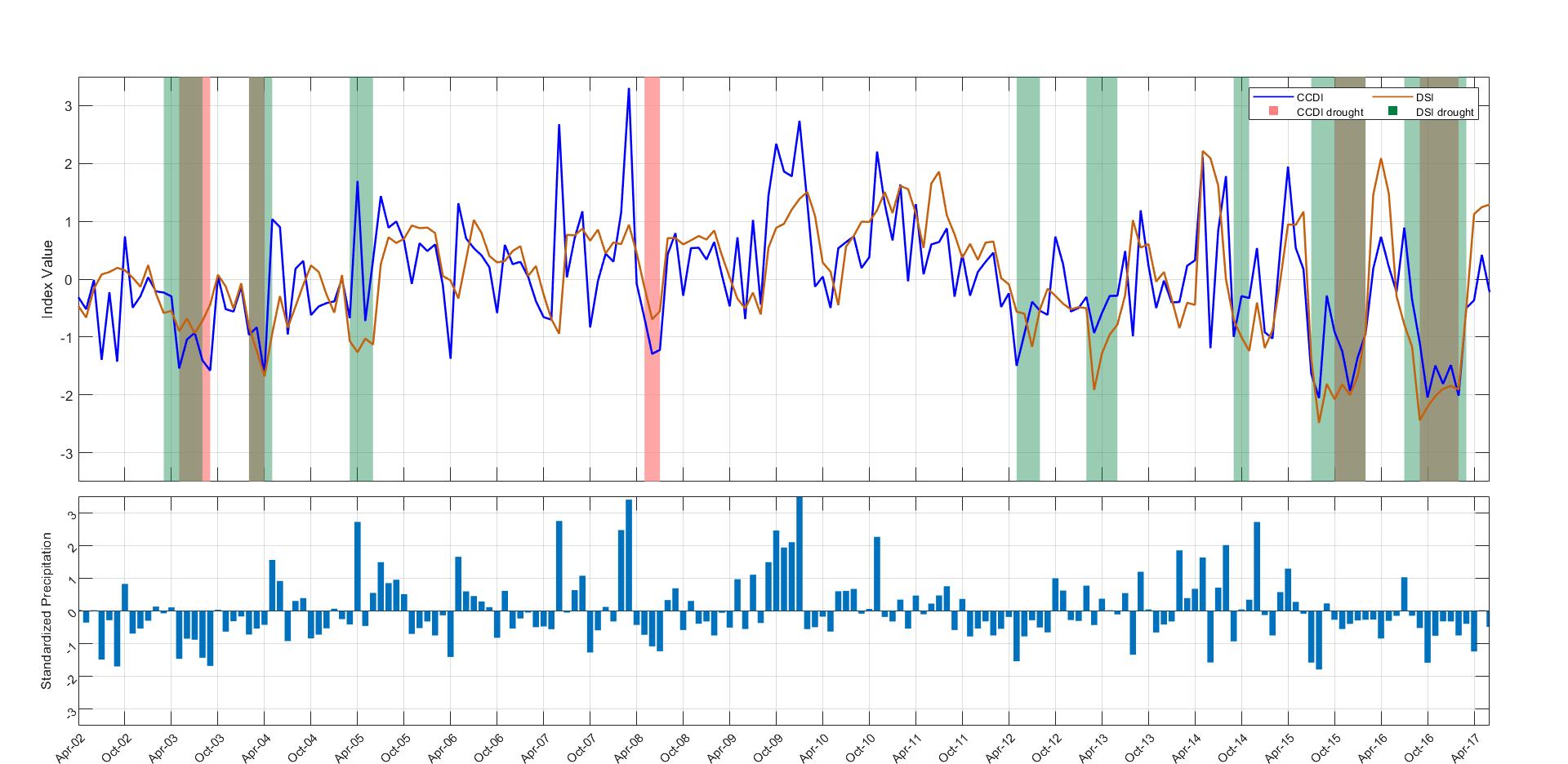
**

(XLI)

**
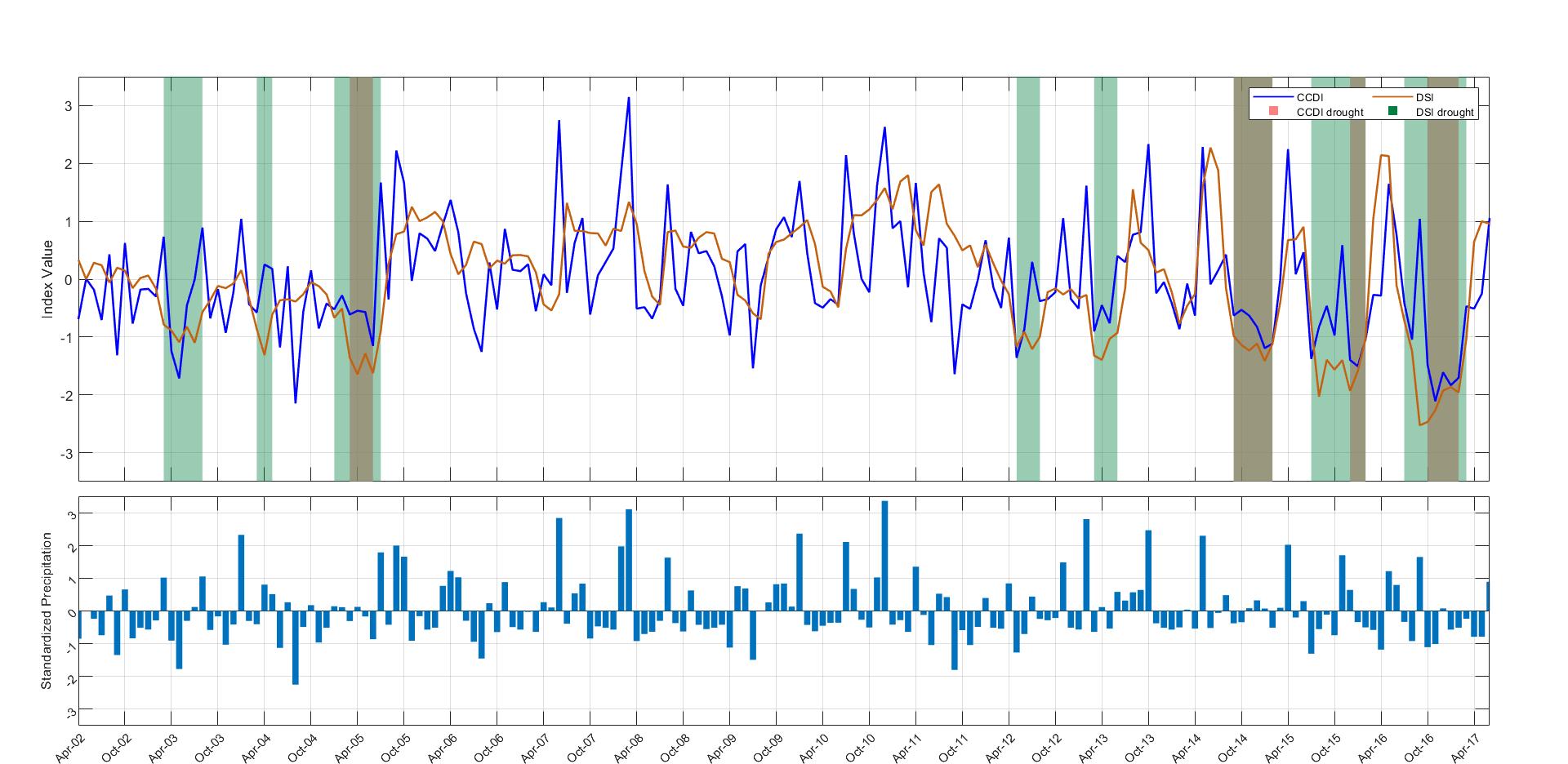
**

(XLII)

**
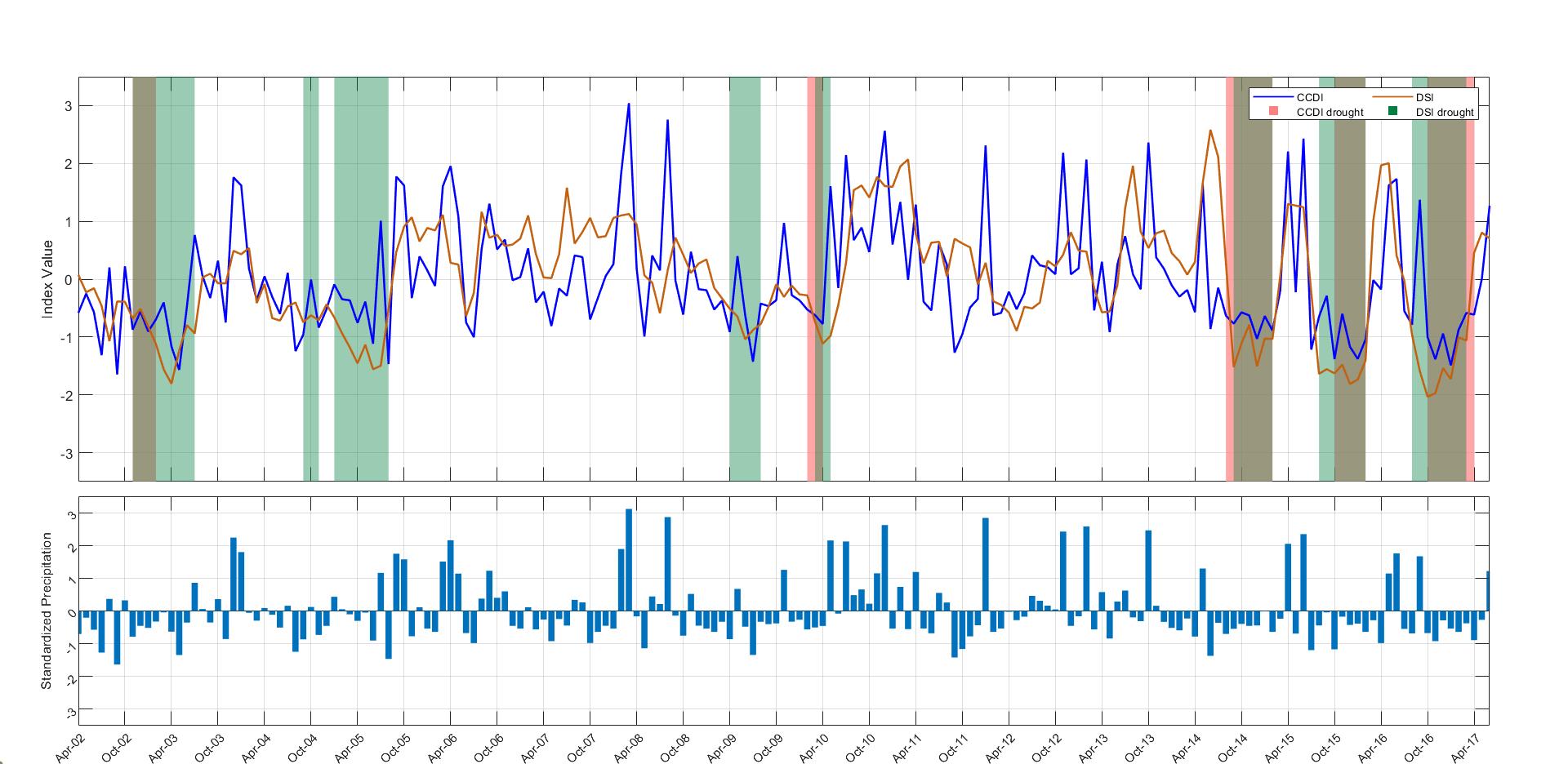
**

(XLIII)

**
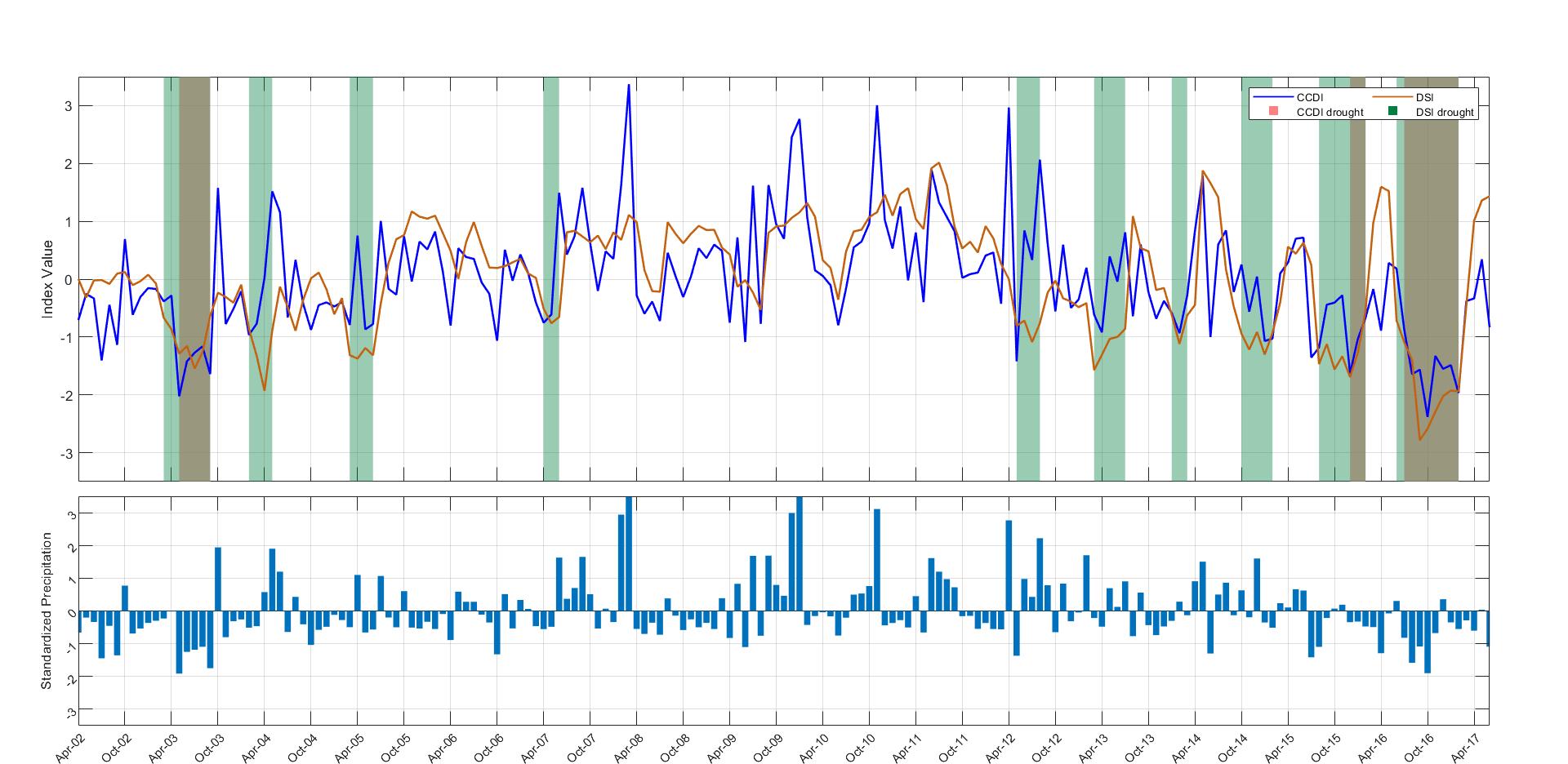
**

(XLIV)

**
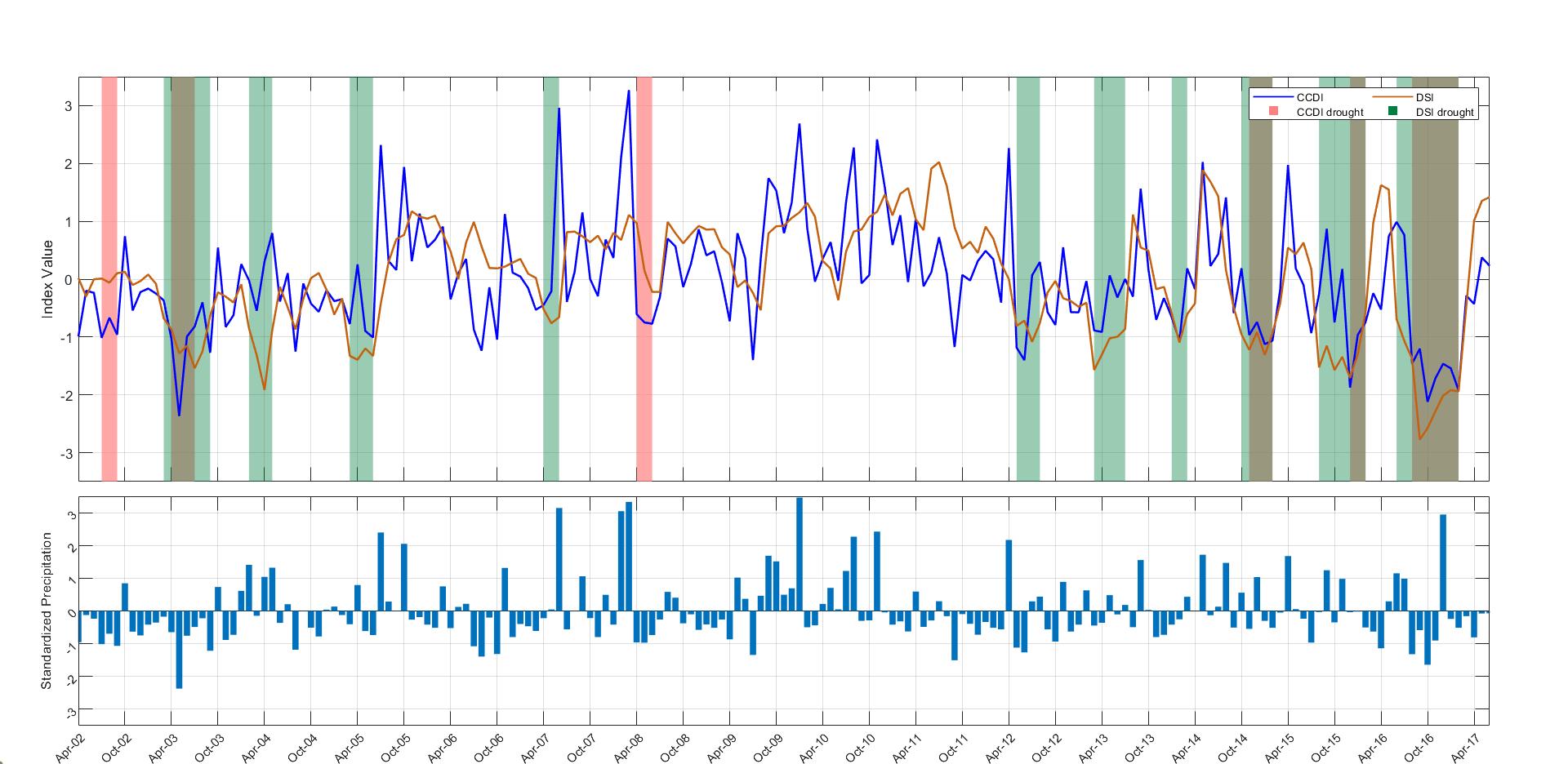
**

(XLV)

**
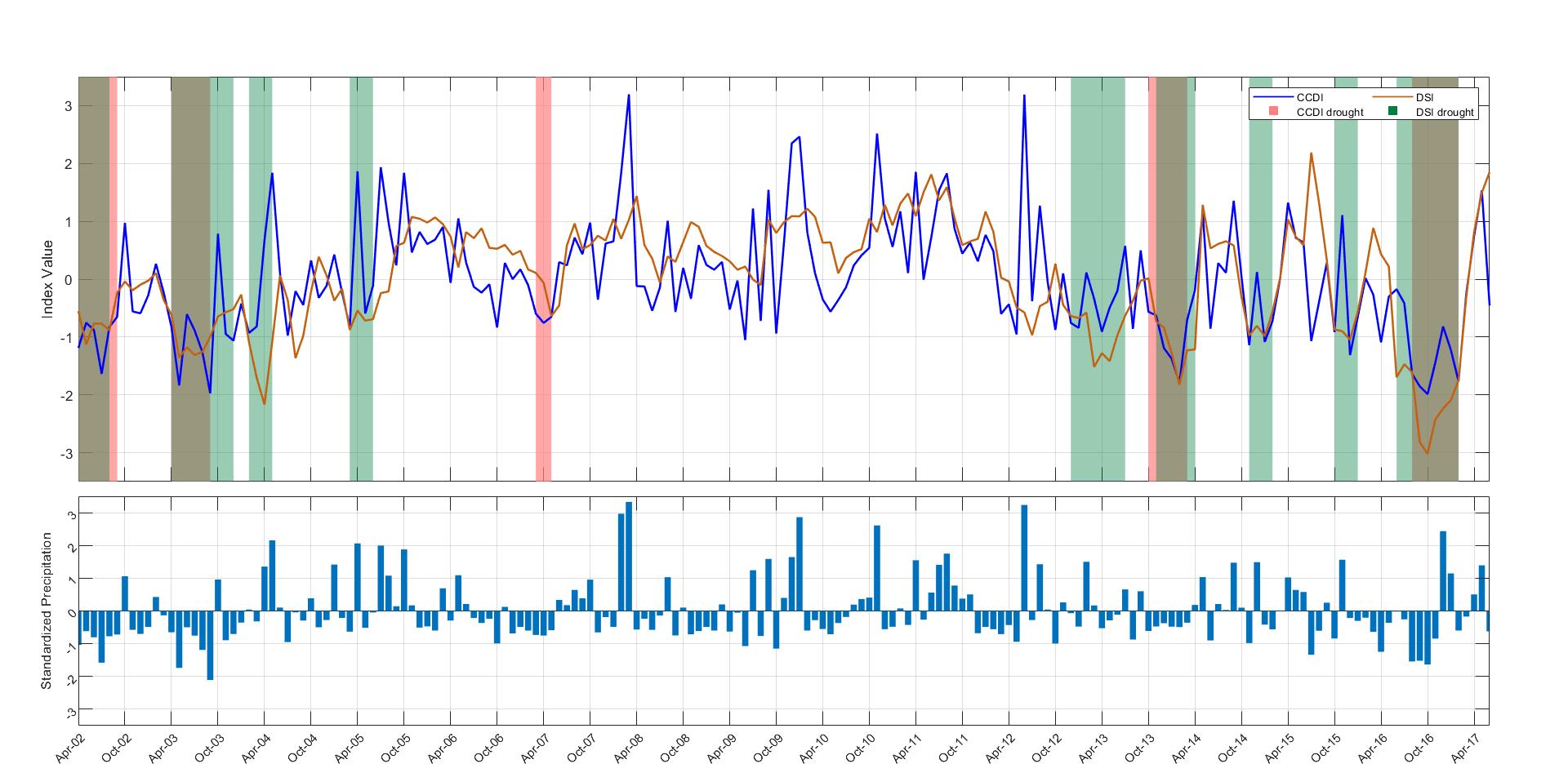
**

(XLVI)

**
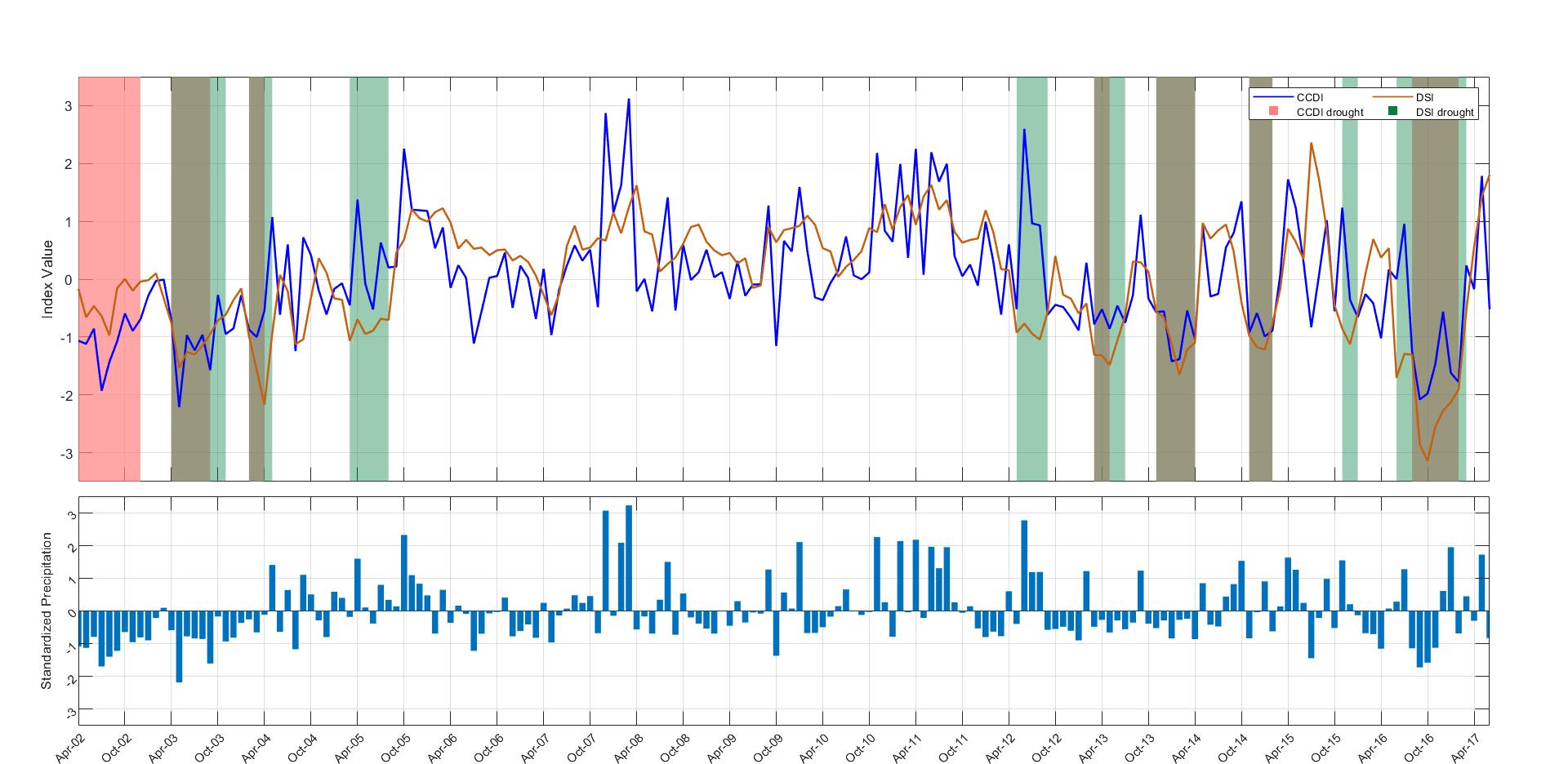
**

(XLVII)

**
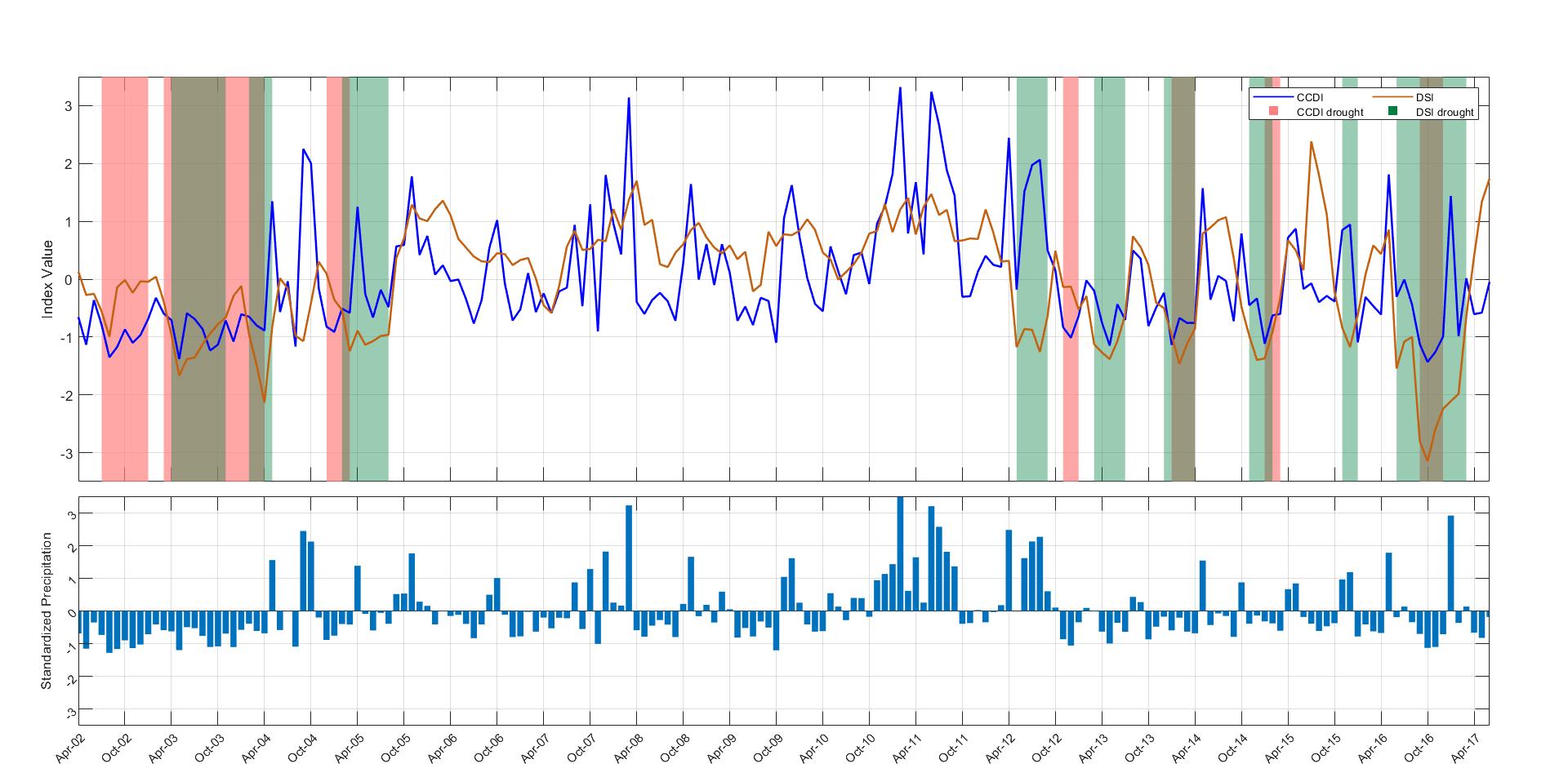
**

(XLVIII)

**
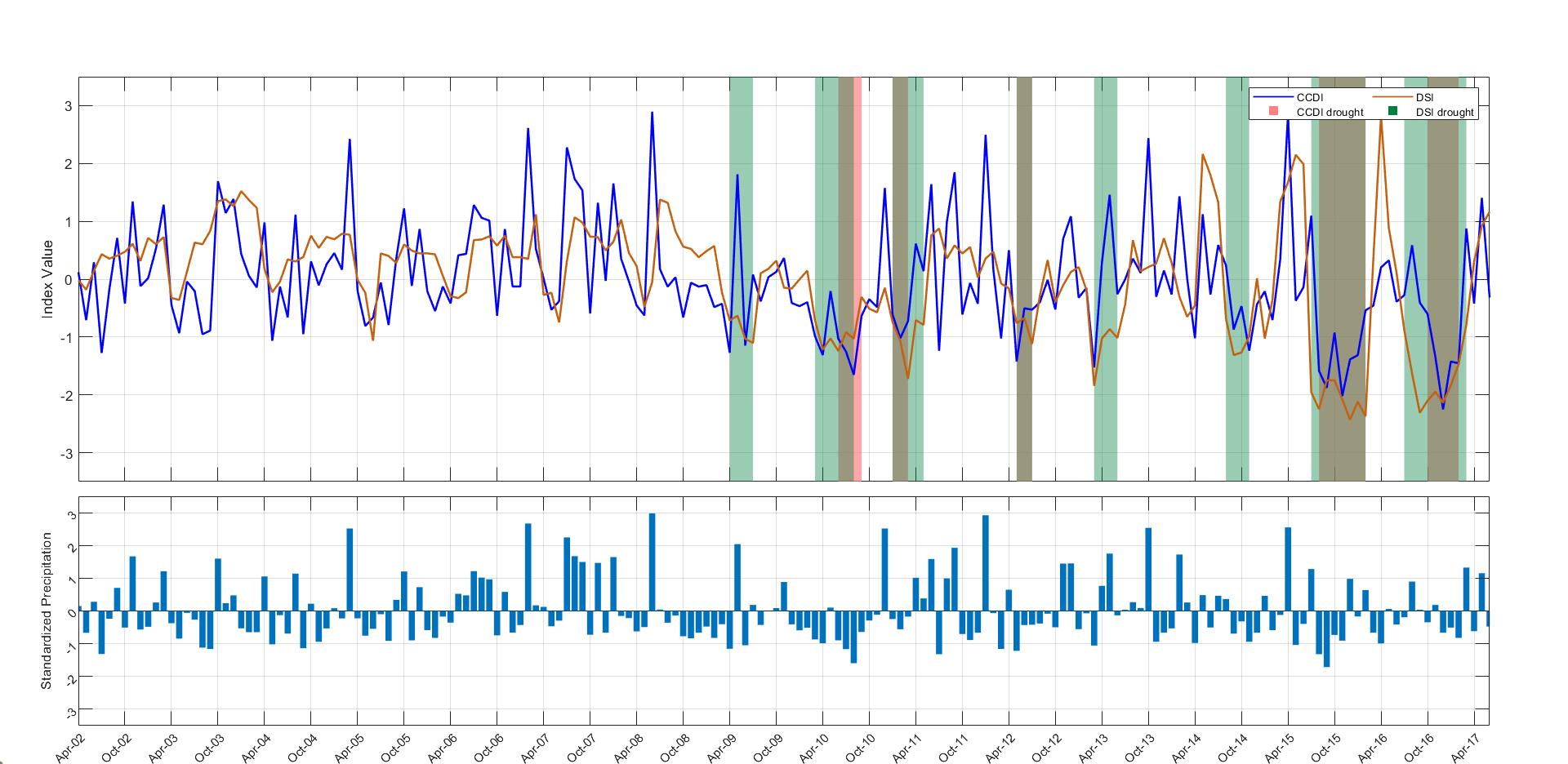
**

(XLIX)

**
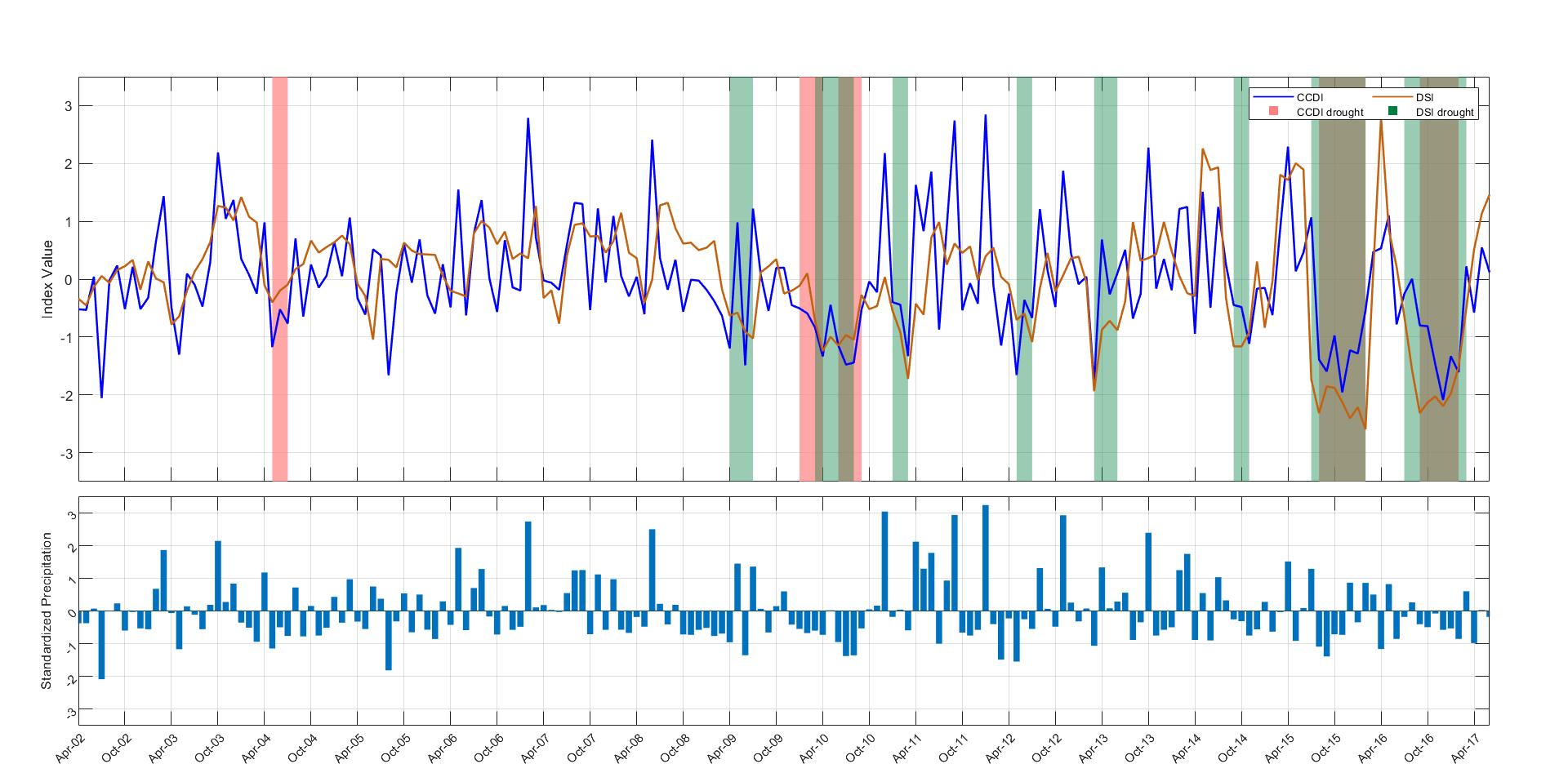
**

(L)

**
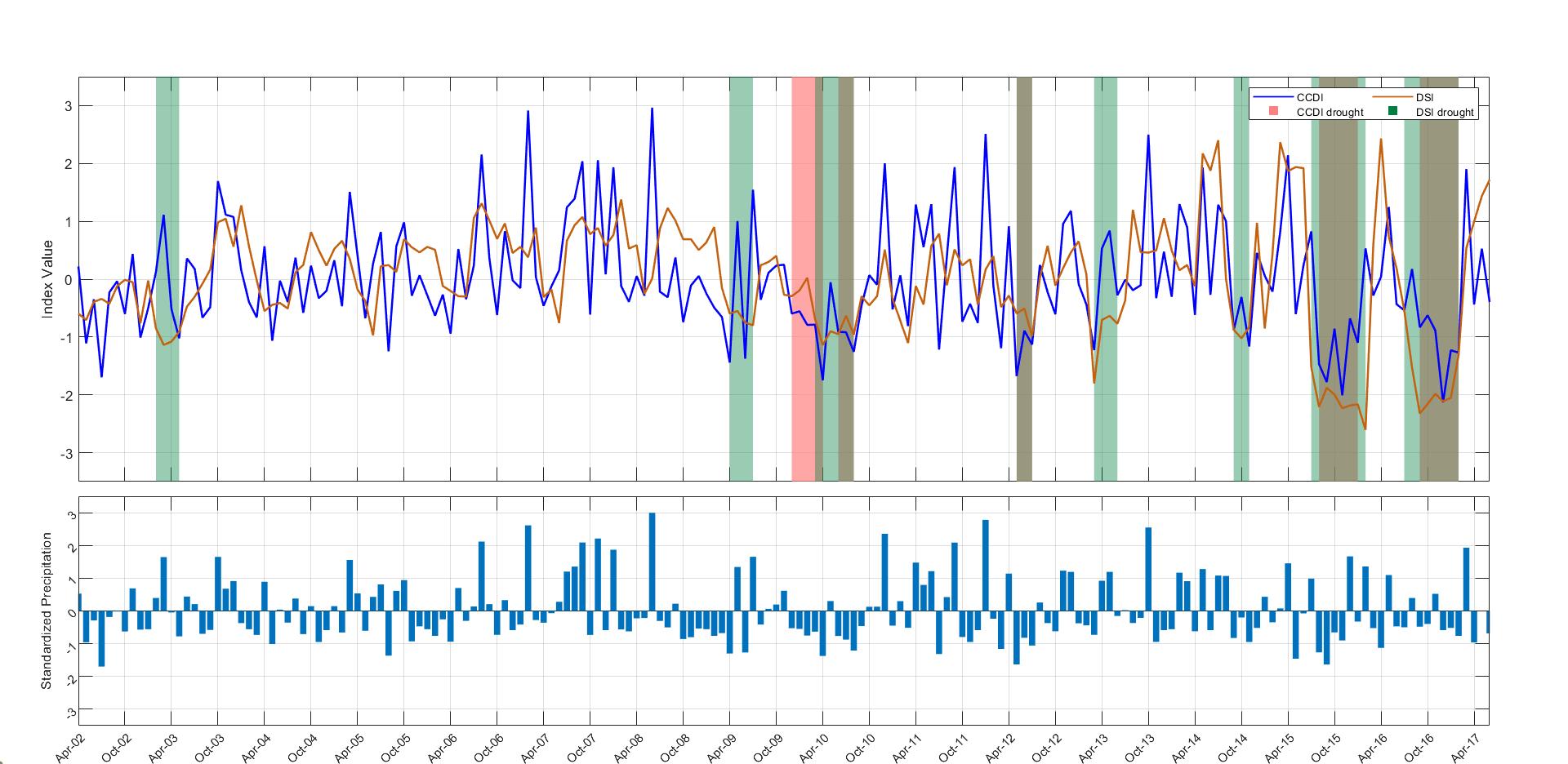
**

(LI)

**
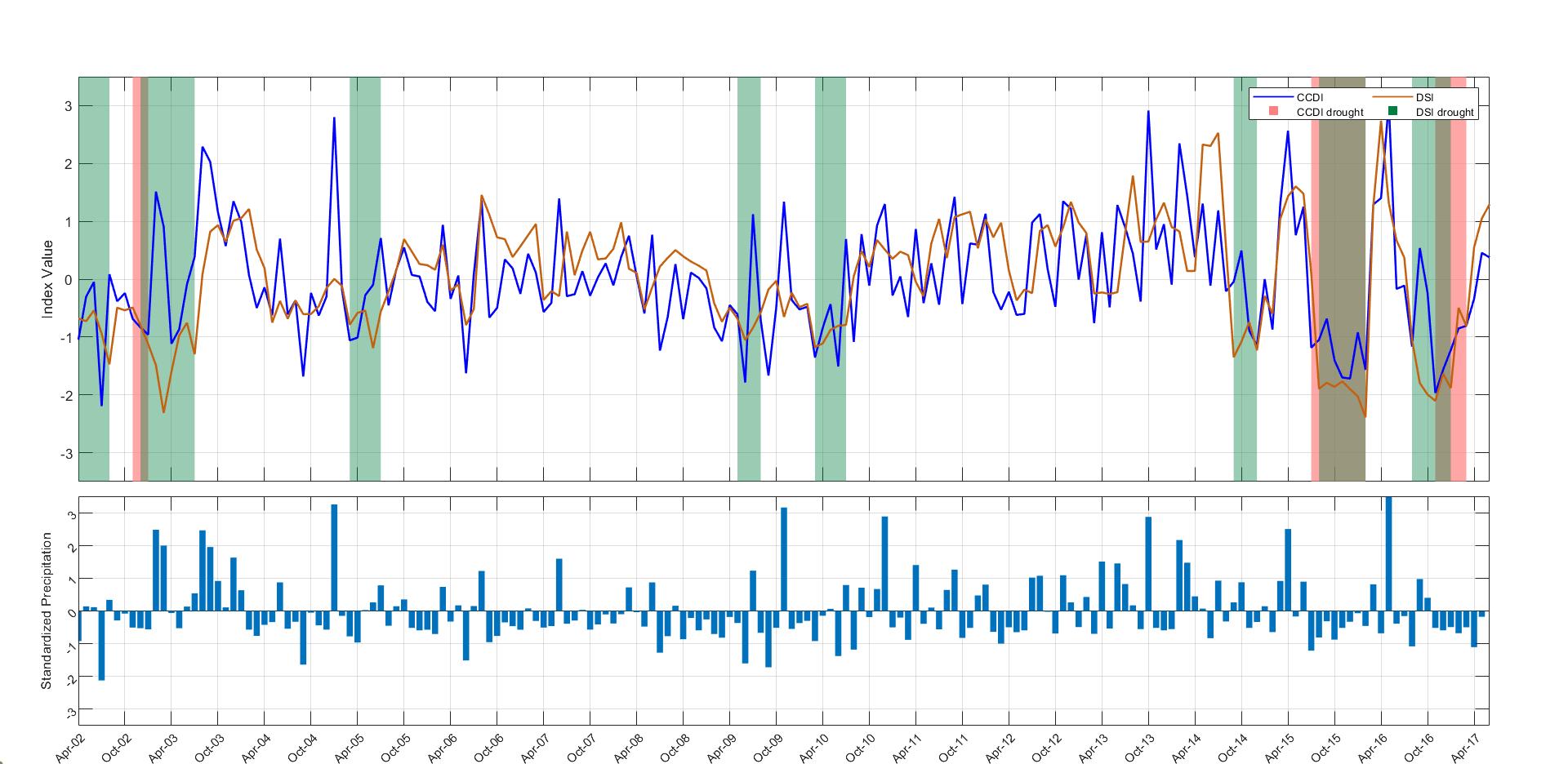
**

(LII)

**
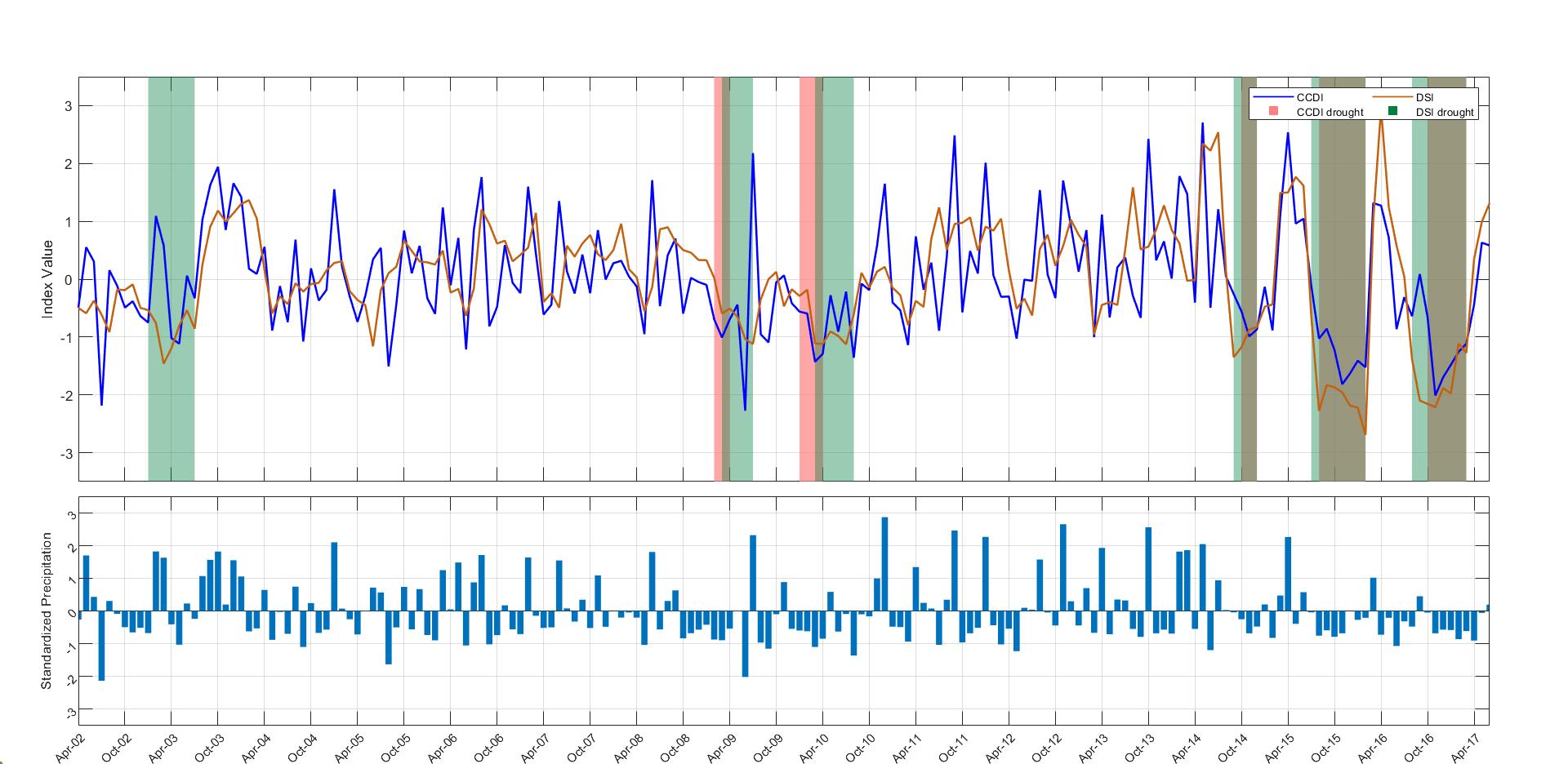
**

(LIII)

**
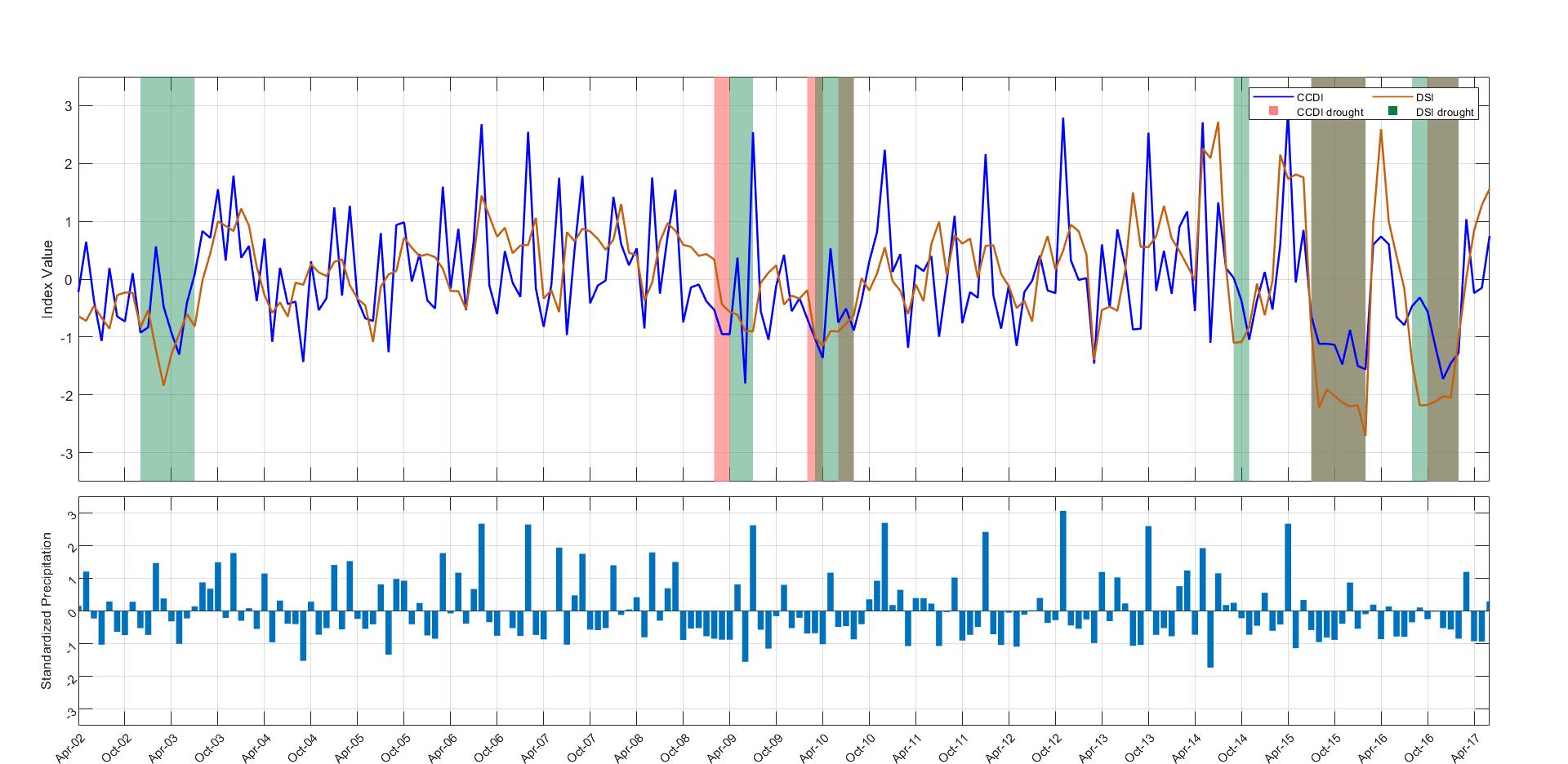
**

(LIV)

**
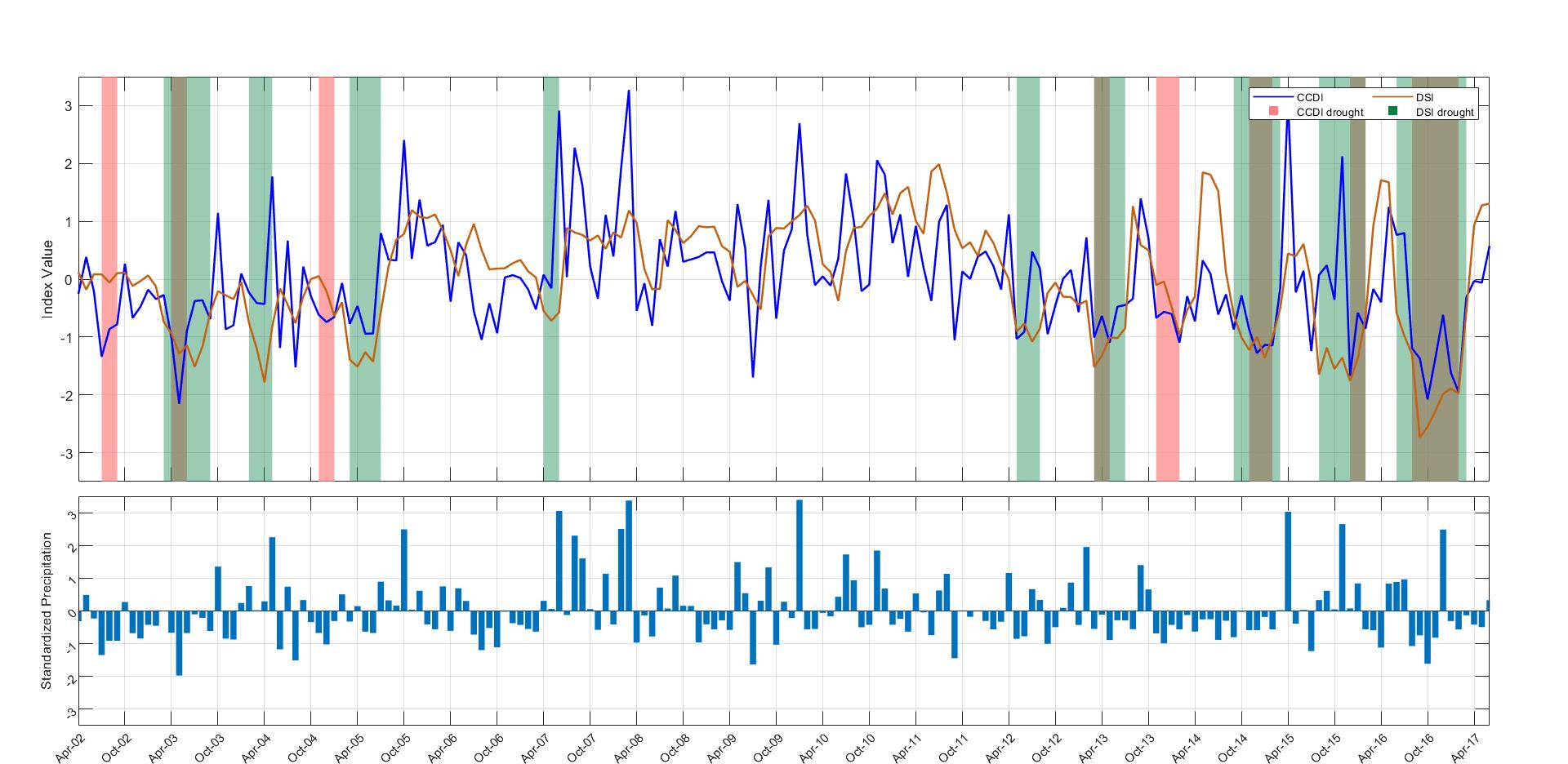
**

(LV)

**
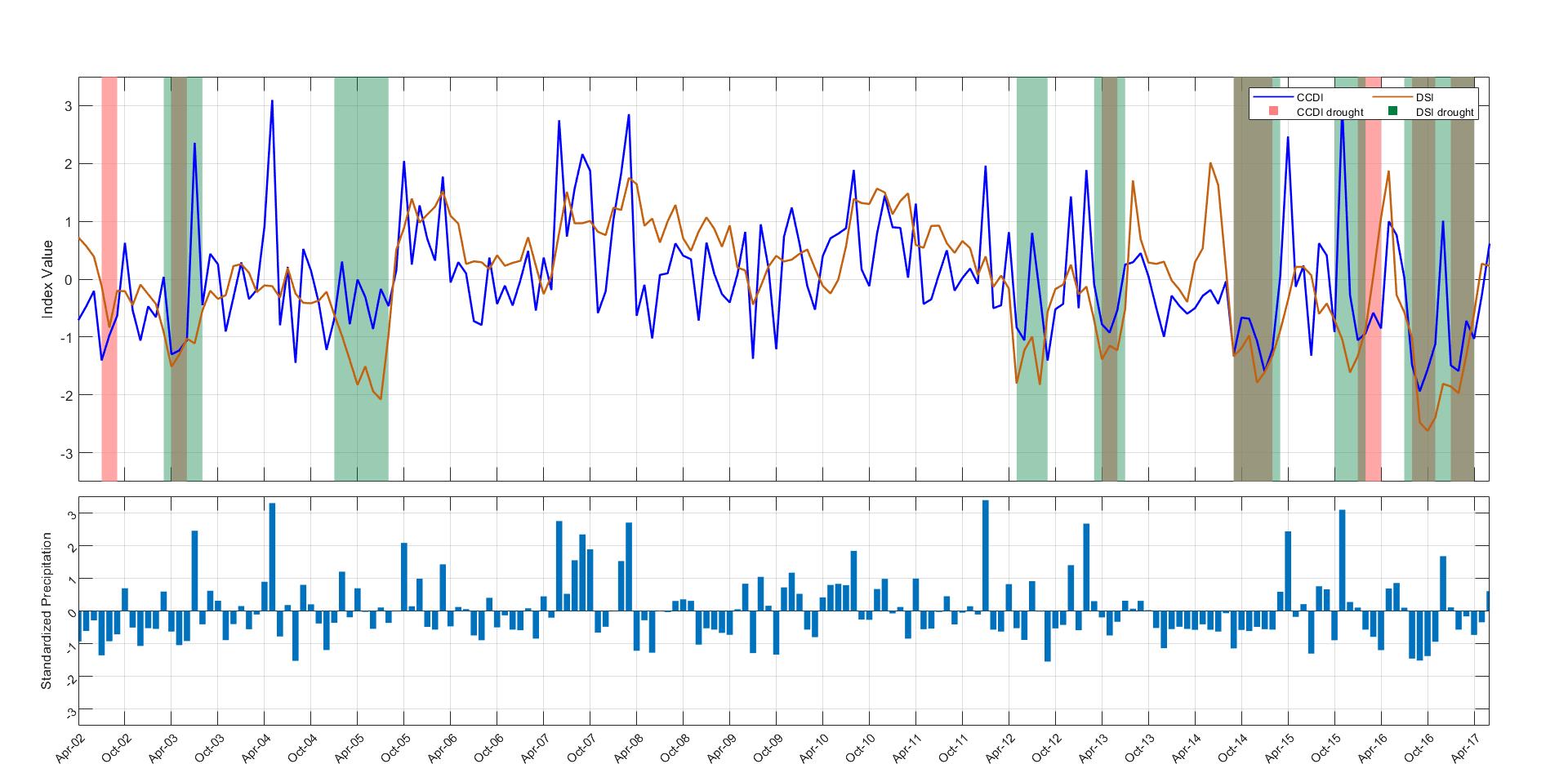
**

(LVI)

**
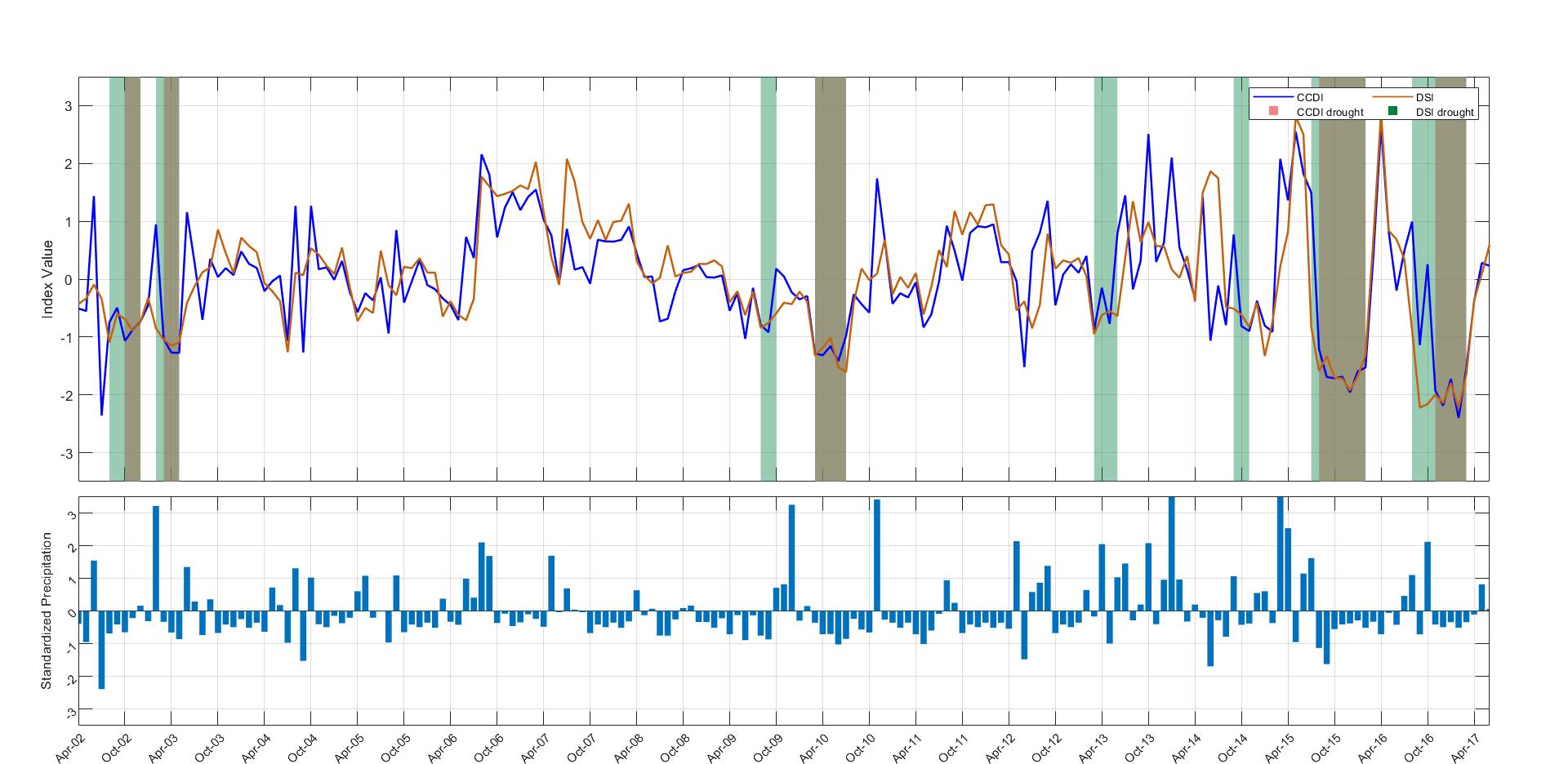
**

(LVII)

**
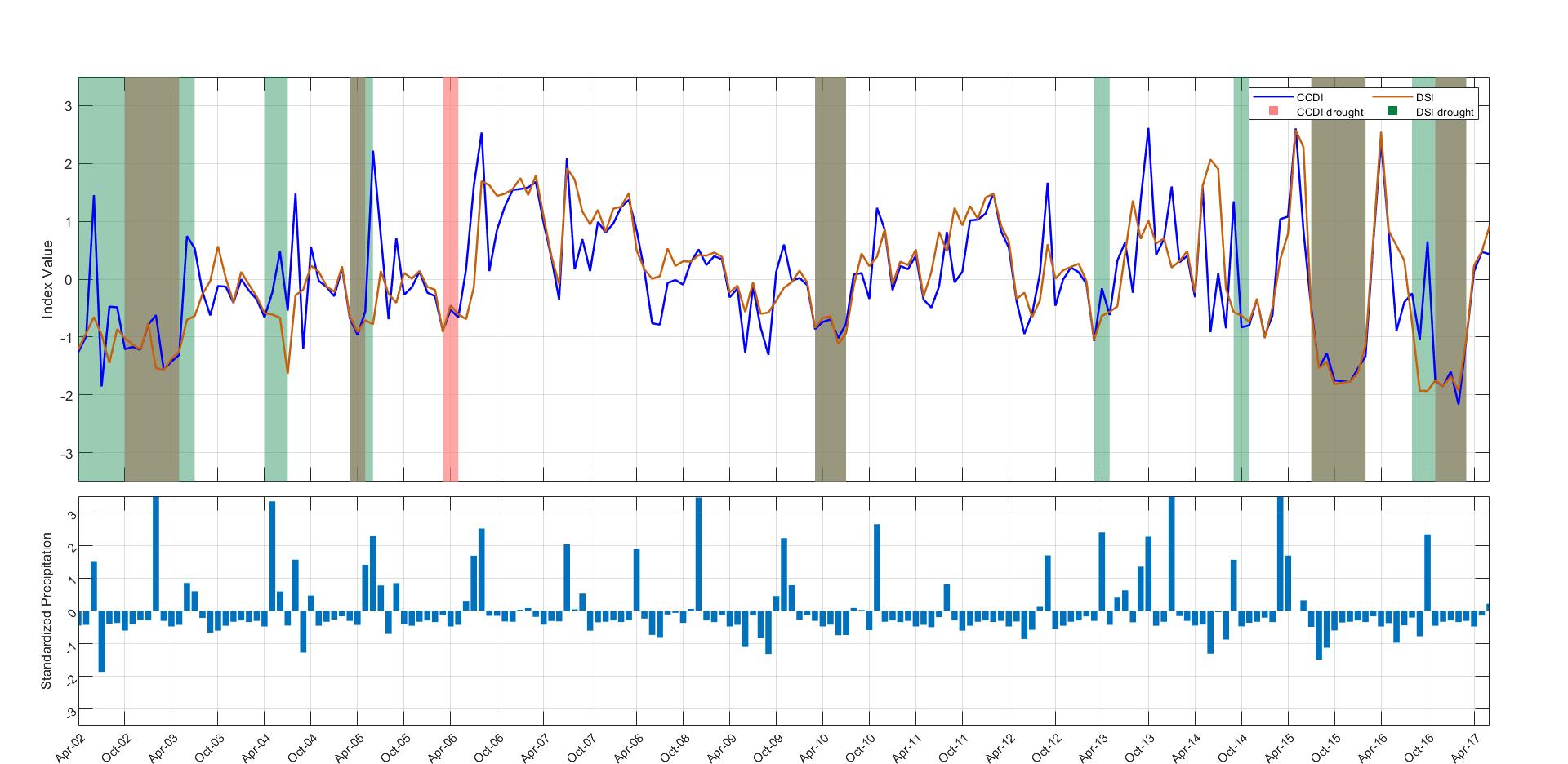
**

(LVIII)

**
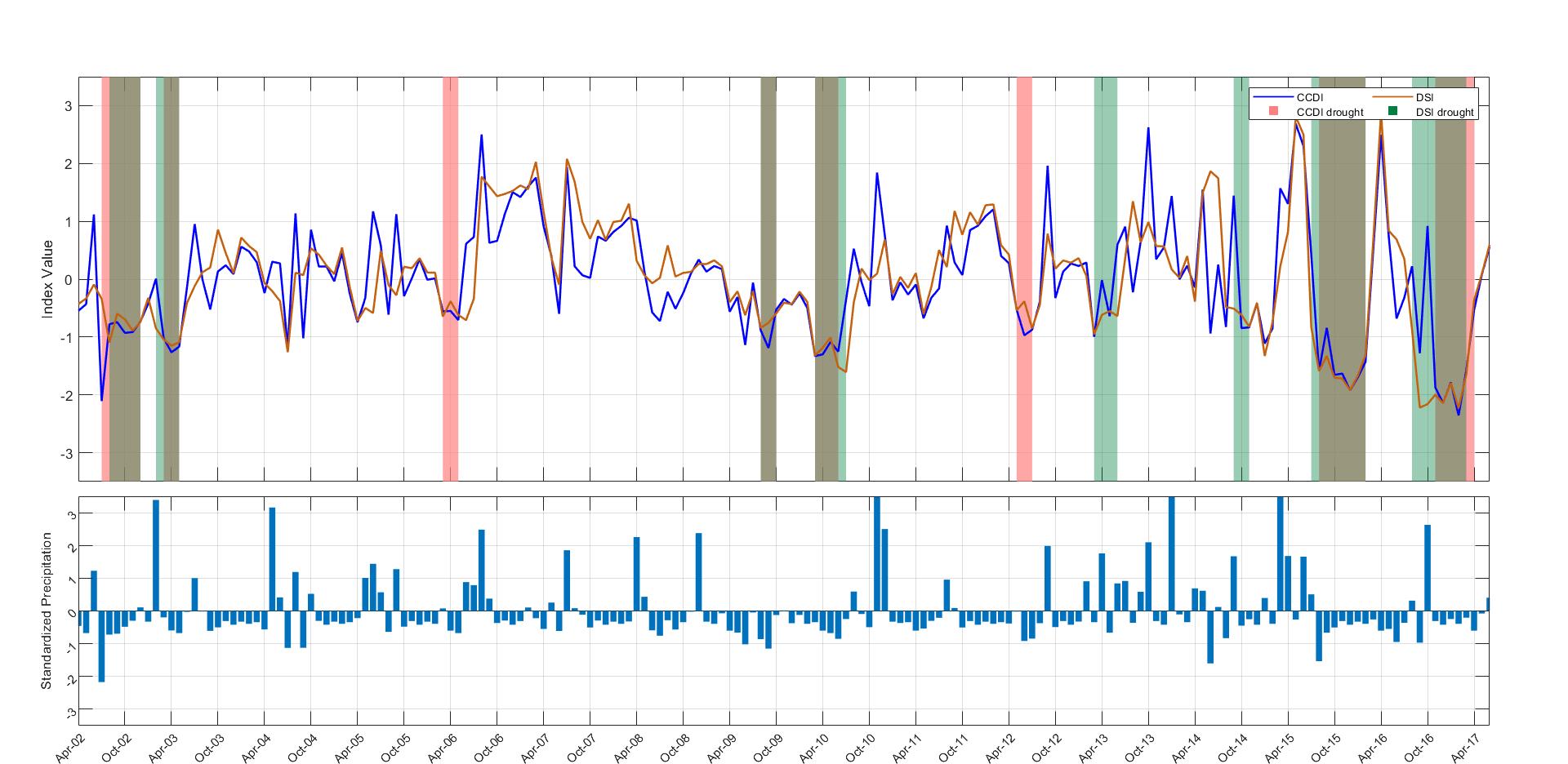
**

(LIX)

**
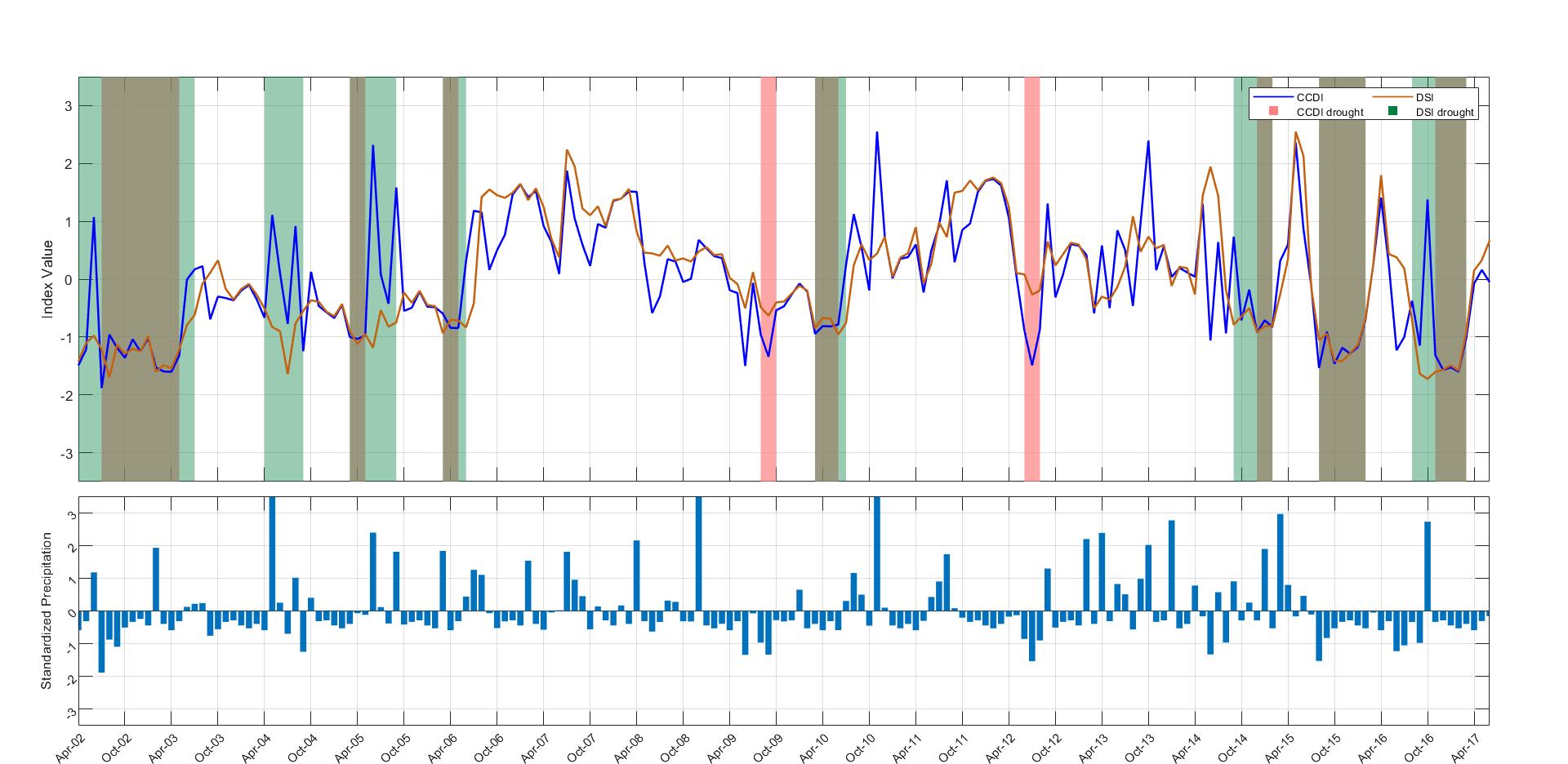
**

(LX)

**
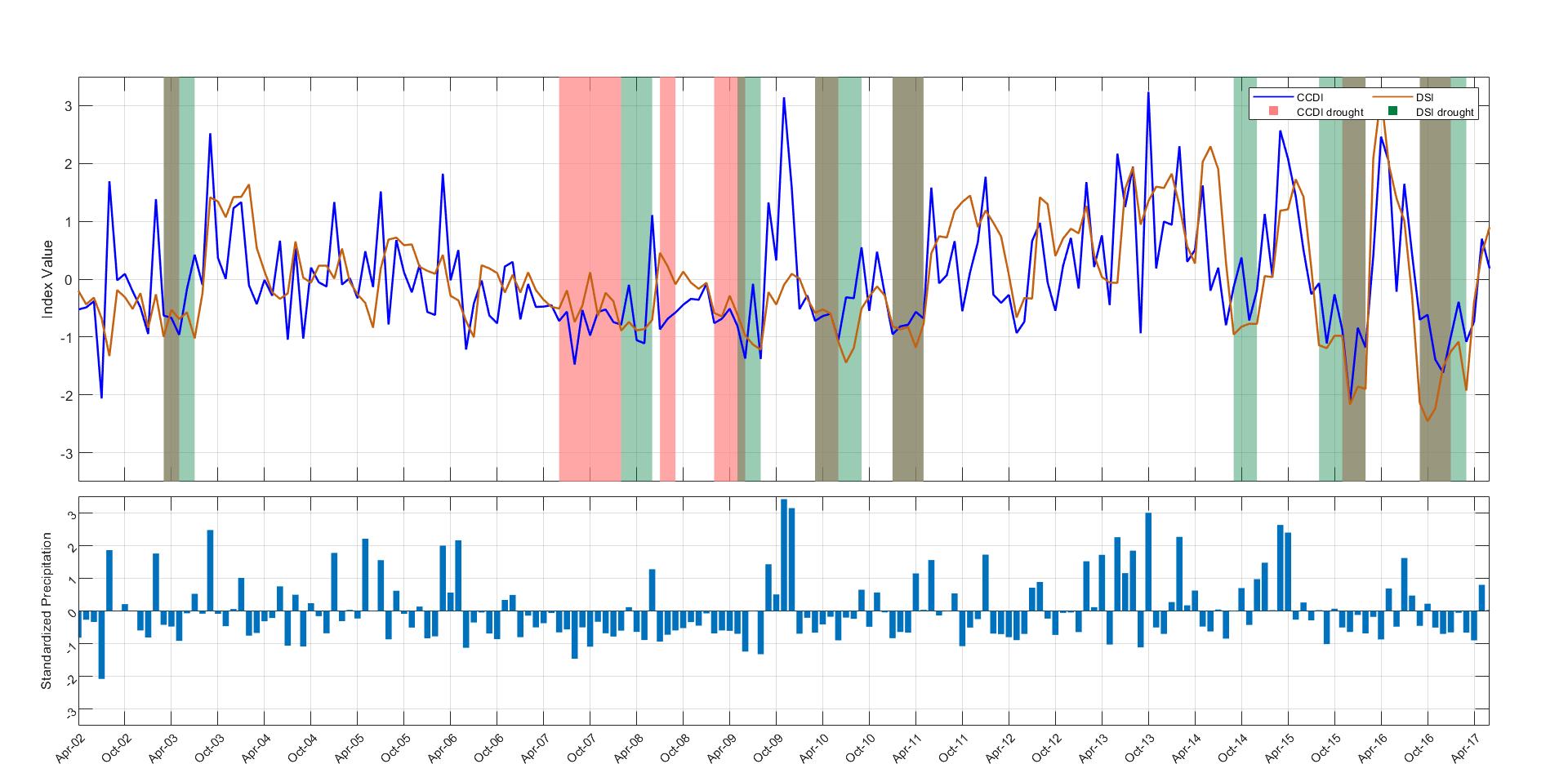
**

(LXI)

**
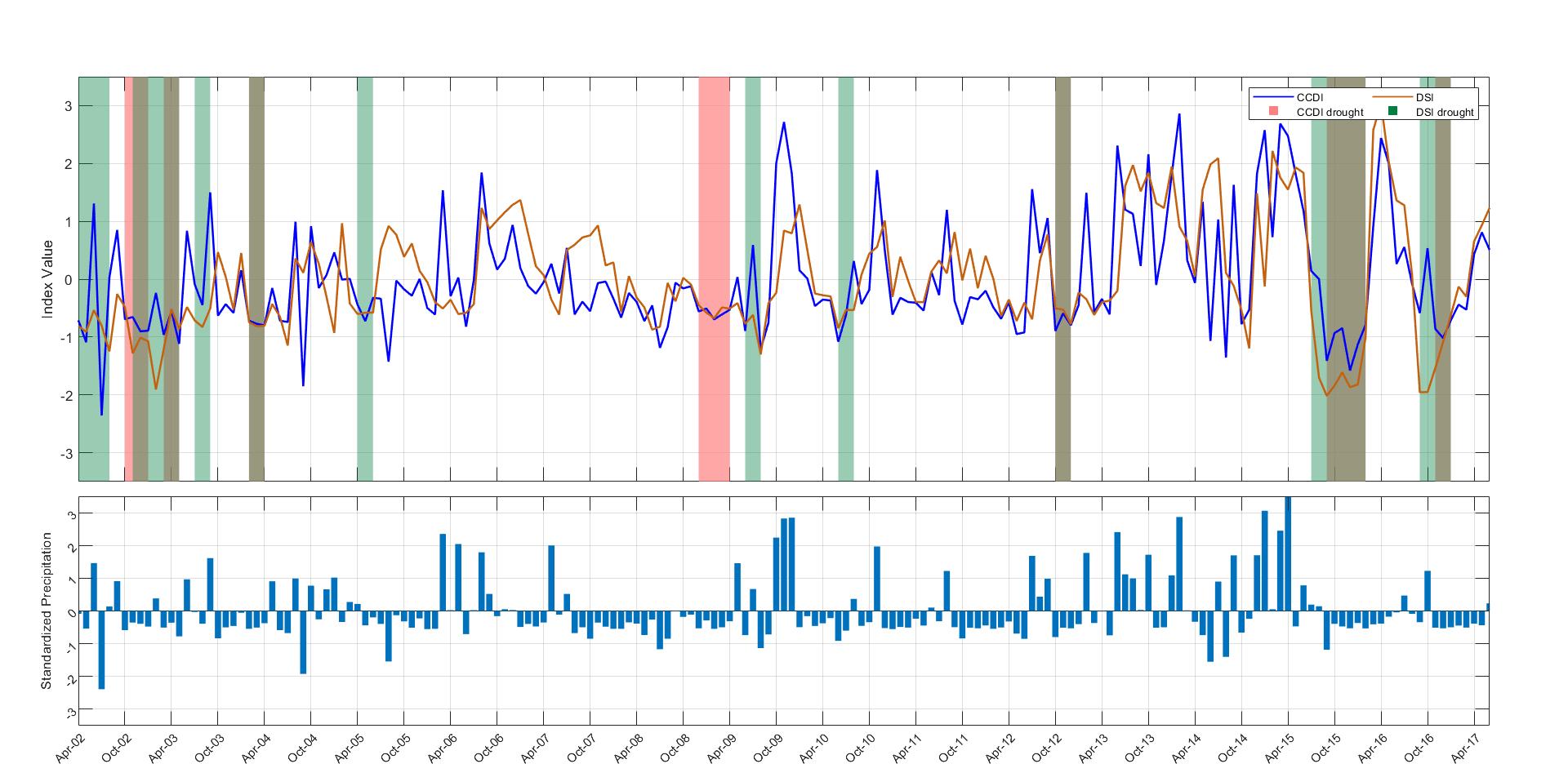
**

(LXII)

**
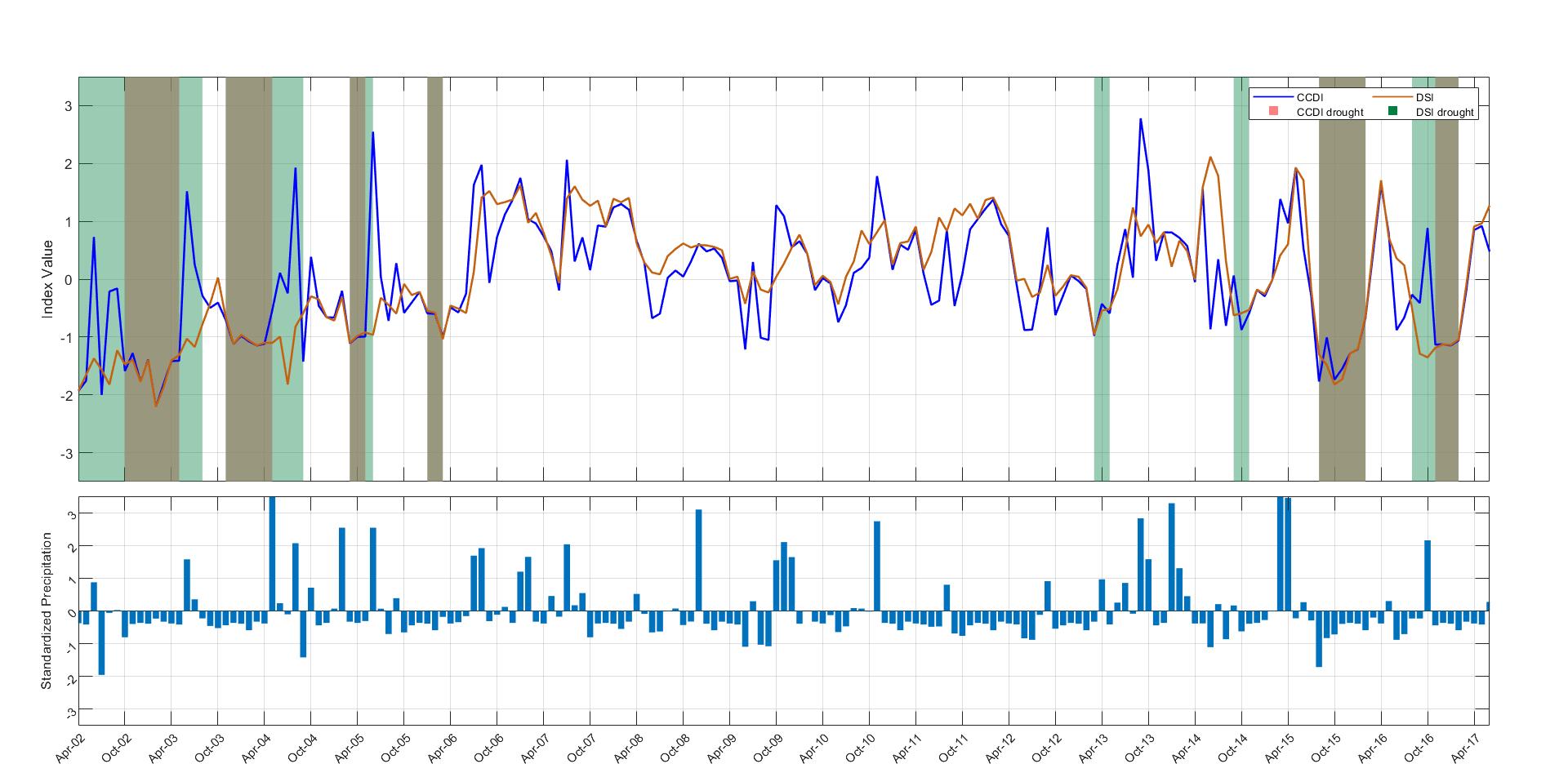
**

(LXIII)

**
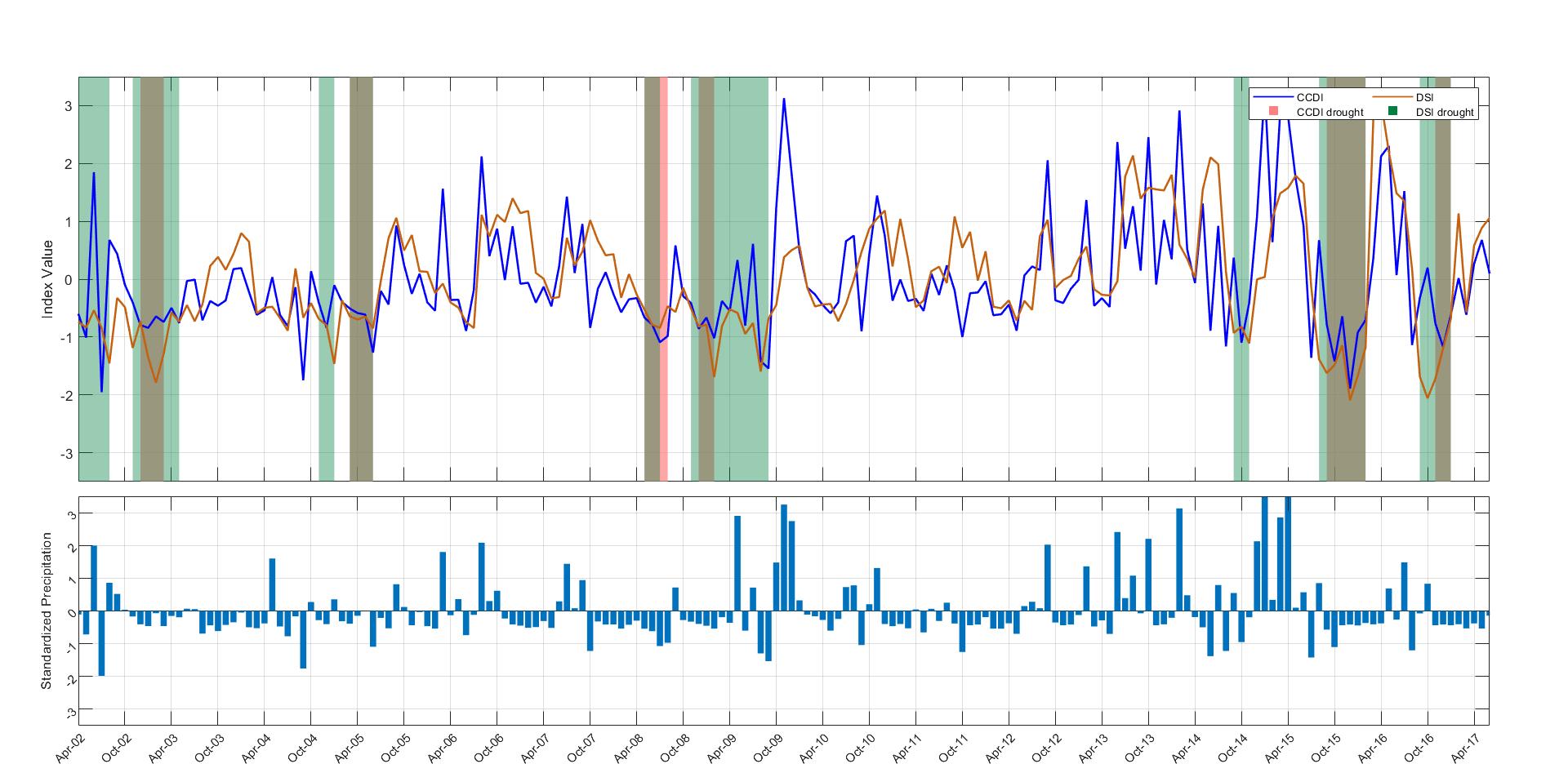
**

(LXIV)

**
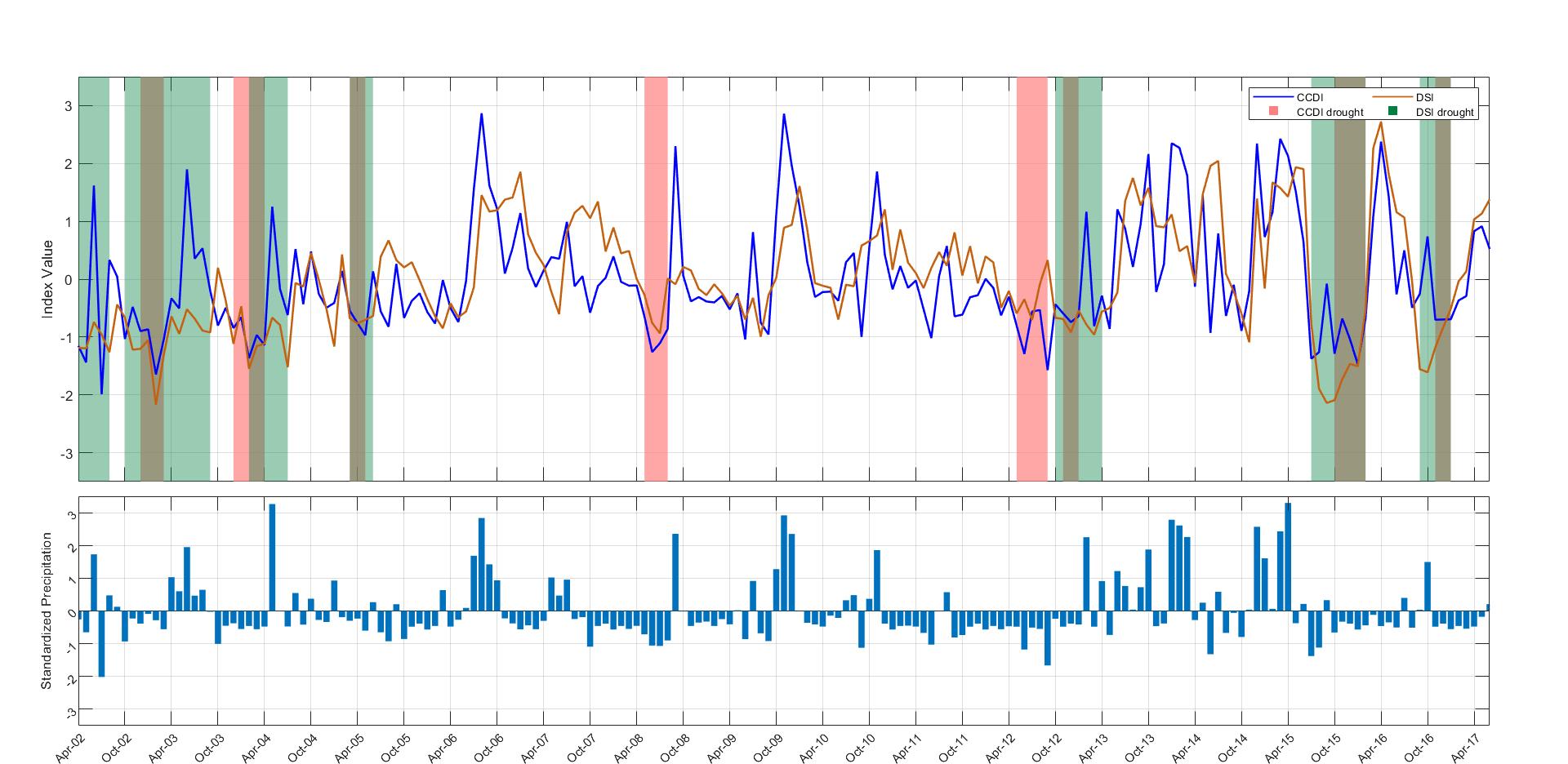
**

(LXV)

**
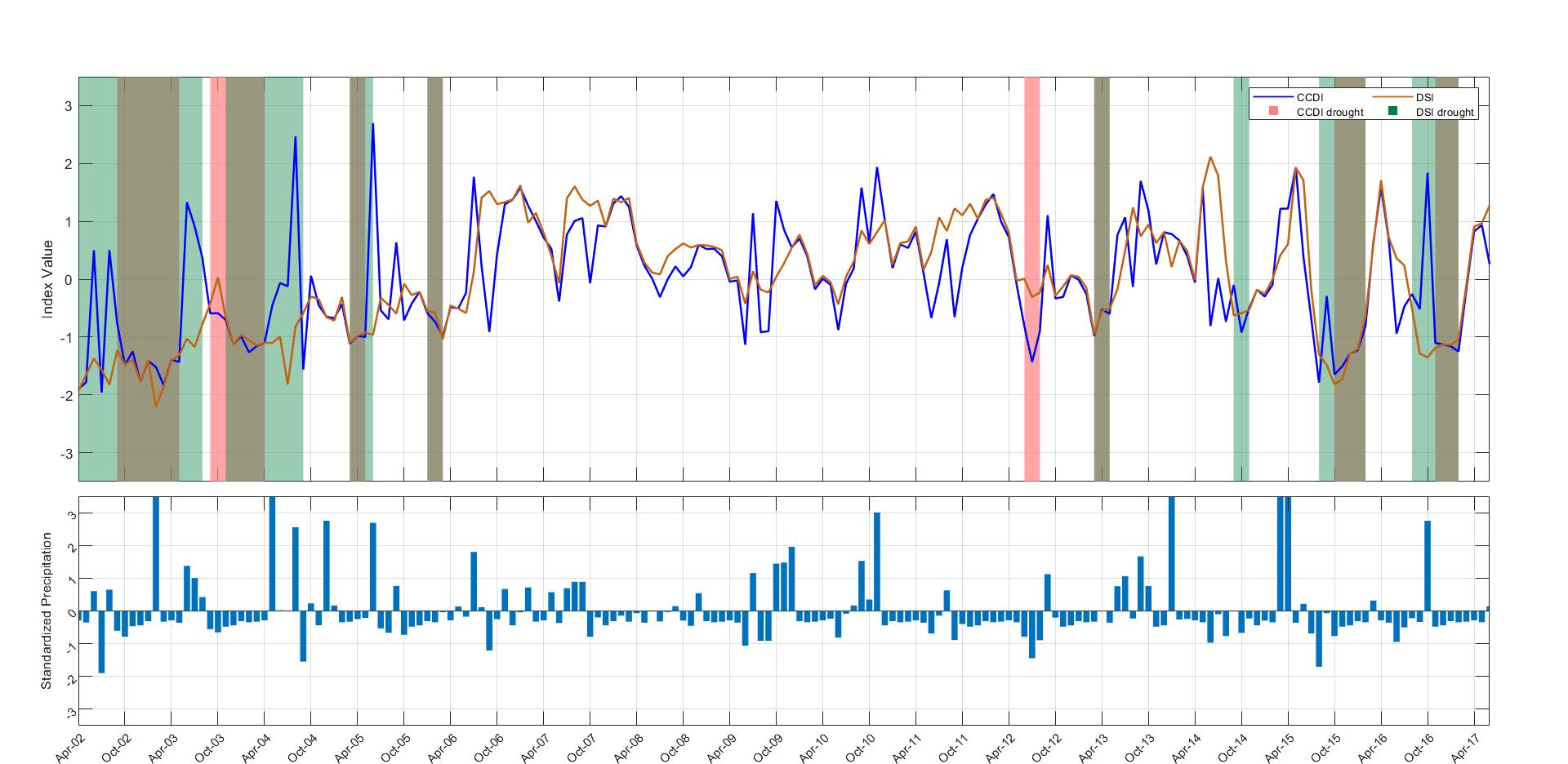
**

(LXVI)

**
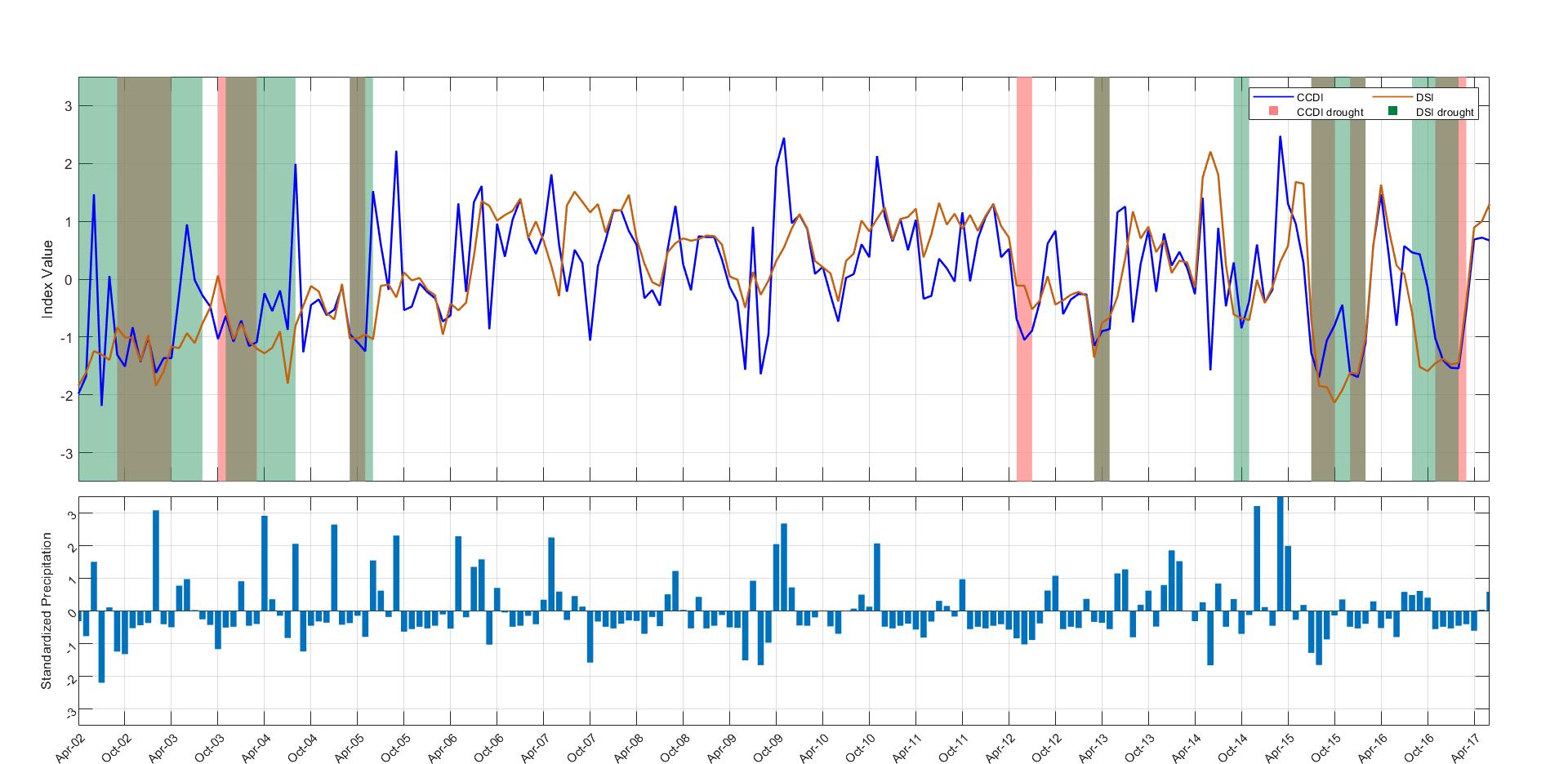
**

(LXVII)

**
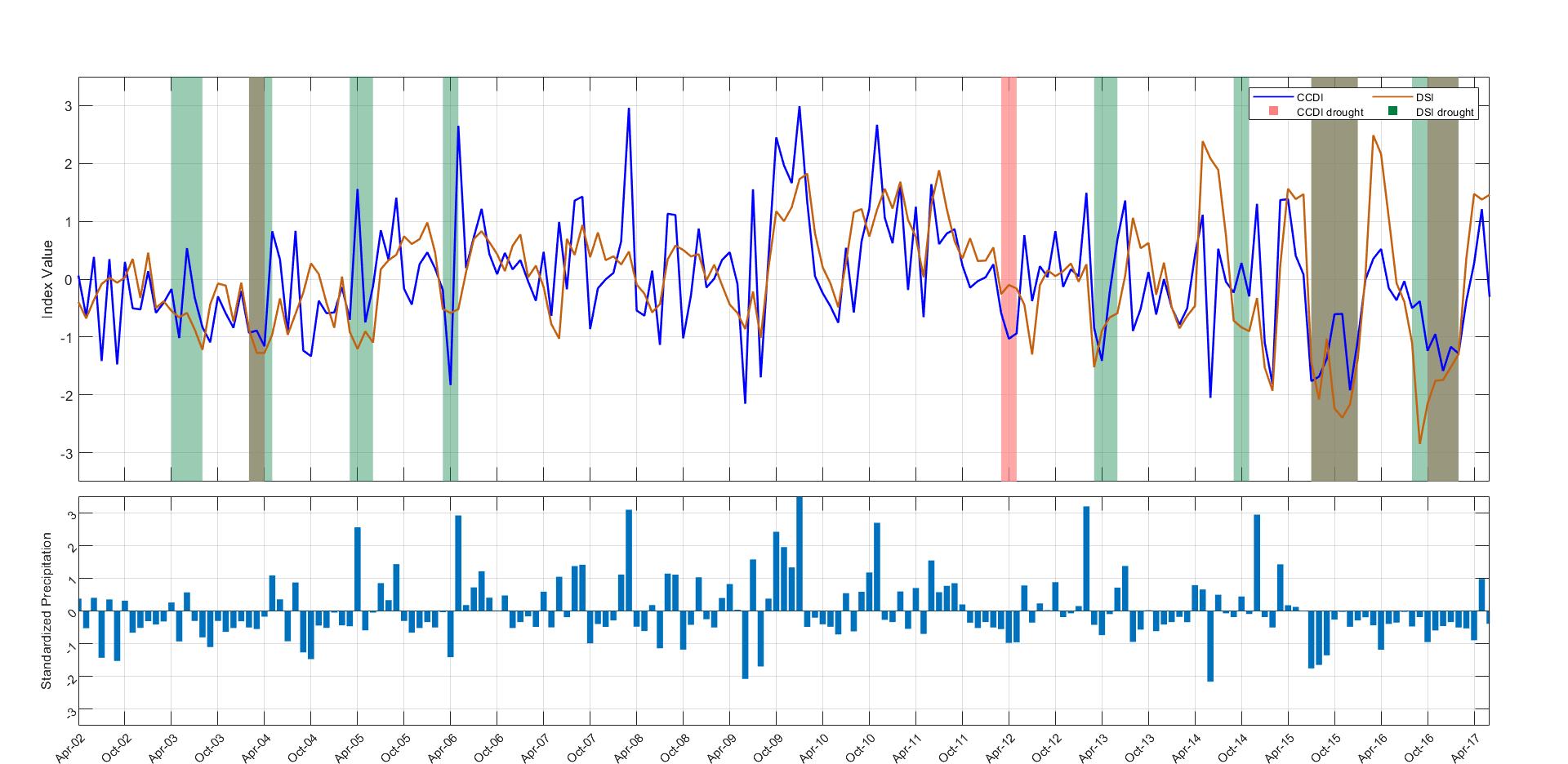
**

(LXVIII)

**
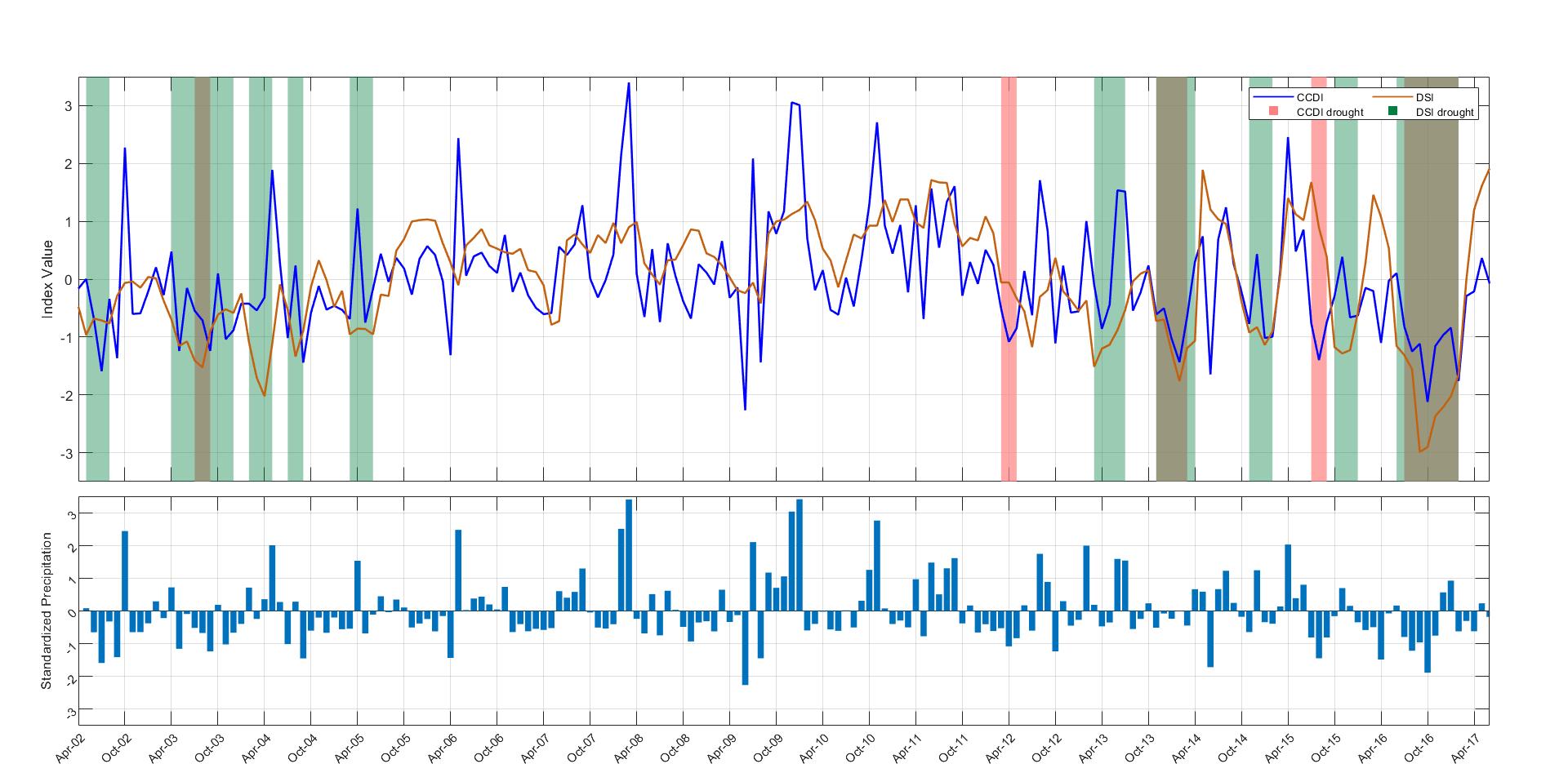
**

(LXIX)

(LXX)

(LXXI)

(LXXII)

(LXXIII)

(LXXIV)

(LXXV)

(LXXVI)

(LXXVII)

(LXXVIII)

(LXXIX)

(LXXX)

(LXXXI)

(LXXXII)

(LXXXIII)

(LXXXIV)

(LXXXV)

**Figure S3.** plots of Drought events identified by CCDI and GRACE-DSI, including the CCDI and GRACE-DSI time series. The standardized precipitation bar graph is also plotted for identification of drought, whether occurring due to deficit in precipitation or deficit in TWS or due to both. Blue lines represent the time-series of CCDI; the orange line represents the time-series of GRACE-DSI, Green bands show drought events identified by GRACE-DSI, and the Pink bands show drought events identified by CCDI. The blue bar graph shows standardized precipitation variation with time. Each plot corresponds to different sub-basins like (I)1a, (II)1b, (III)1e, (IV)1f, (V)1g, (VI)1h, (VII)1i, (VIII)1j, (IX)1k, (X)2a, (XI)2d, (XII)2e, (XIII)2f, (XIV)2g, (XV)2h, (XVI)2i, (XVII)2j, (XVIII)2k, (XIX)2l, (XX)2m. (XXI)2n, (XXII)2o, (XXIII)2p, (XXIV)2q, (XXV)2r, (XXVI)2s, (XXVII)3a, (XXVIII)3b, (XXIX)4, (XXX)5a, (XXXI)5b, (XXXII)5c, (XXXIII)5d, (XXXIV)5e, (XXXV)5f, (XXXVI)5g, (XXXVII)5h, (XXXVIII)6a, (XXXIX)6b, (XL)6c, (XLI)6d, (XLII)6e, (XLIII)6f, (XLIV)6g, (XLV)7a, (XLVI)7b, (XLVII)7c, (XLVIII)8, (XLIX)9a, (L)9b, (LI)10a, (LII)10b, (LIII)10c, (LIV)11a, (LV)11b, (LVI)12a, (LVII)12b, (LVIII)13a, (LIX)13b, (LX)14a, (LXI)14b, (LXII)14c, (LXIII)15a, (LXIV)15b, (LXV)15c, (LXVI)16a, (LXVII)16b, (LXVIII)17a, (LXIX)17b, (LXX)17c, (LXXI)18a, (LXXII)18b, (LXXIII)19, (LXXIV)20, (LXXV)21a, (LXXVI)21b, (LXXVII)22a, (LXXVIII)22b, (LXXIX)23a, (LXXX)23b, (LXXXI)23c, (LXXXII)23d, (LXXXIII)23e, (LXXXIV)23f, (LXXXV)24. we have named sub-basins by notation here; refers to Table 1 for detailed sub-basins names with notation.

**Table S2.** Drought Events obtained through CCDI over major sub-basins of India. DE represents drought events. We have named sub-basins with notation 1a,1b…etc. Detailed naming and notation of basins and sub-basins are presented in Appendix 1. The drought duration and drought severity of each event are listed in the table. Drought duration is the period of time where the CCDI value is below the fixed threshold value (for this study, we have taken threshold value =-0.5). Drought Severity is the cumulative value of CCDI within the drought duration.

| Basin | Sub-basins | Drought events | Start month | End month | Drought duration  (months) | Drought Severity |
| --- | --- | --- | --- | --- | --- | --- |
| 1 | 1a  1b  1c  1d  1e  1f  1g  1h  1i  1j  1k | DE1  DE2  DE3  DE1  DE2  DE1  DE2  DE3  DE4  DE1  DE2  DE3  DE4  DE5  DE6  DE7  DE1  DE2  DE3  DE1  DE2  DE3  DE1  DE2  DE3  DE1  DE2  DE1  DE2  DE3  DE4  DE5  DE6  DE1  DE2  DE3  DE4  DE1  DE2  DE3 | Sept 2007  Jan 2009  Dec 2009  Feb 2009  Apr 2016  July 2009  Oct 2012  Sept 2013  Sept 2016  Apr 2002  Jan 2004  Jan 2008  Jan 2013  July 2013  Dec 2013  Jan 2016  Aug 2007  Dec 2009  Sept 2016  July 2007  Oct 2012  Sept 2016  Dec 2009  Oct 2012  Sept 2016  Oct 2012  Sept 2016  Feb 2011  Dec 2011  July 2013  Aug 2014  Aug 2015  Sept 2016  Dec 2009  Aug 2015  Apr 2016  Sept 2016  Aug 2015  Apr 2016  Feb 2017 | Dec 2007  May 2009  Apr 2010  May 2009  Dec 2016  Oct 2009  Jan 2013  Dec 2013  Dec 2016  Aug 2002  July 2004  Oct 2008  May 2013  Oct 2013  June 2015  Oct 2016  Nov 2007  March 2010  Dec 2016  Nov 2007  Jan 2013  Dec 2016  Apr 2010  Jan 2013  Dec 2016  Jan 2013  Dec 2016  May 2011  June 2012  Dec 2013  Nov 2014  Feb 2016  Dec 2016  June 2010  Feb 2016  July 2016  May 2017  Feb 2016  Dec 2016  May 2017 | 3  4  4  3  8  3  3  3  3  4  6  8  4  3  17  9  3  3  3  4  3  3  4  3  3  3  3  3  6  5  3  6  3  6  6  3  8  6  8  3 | -3.41903  -5.13296  -4.87092  -3.26845  -7.57409  -3.14157  -3.58596  -2.58055  -4.36056  -3.31506  -4.87769  -6.34177  -3.61905  -2.58862  -13.4392  -8.30728  -3.55418  -3.04673  -3.79706  -3.37578  -4.81273  -4.799  -5.72904  -4.86557  -5.08557  -4.58183  -5.42948  -3.51414  -7.15261  -4.37263  -4.33074  -5.71451  -6.43464  -6.21096  -8.35479  -3.81963  -16.6395  -8.78771  -12.6304  -7.68516 |
| 2 | 2a  2b  2c  2d  2e  2f  2g  2h  2i  2j  2k  2l  2m  2n  2o  2p  2q  2r  2s | DE1  DE2  DE3  DE4  DE1  DE2  DE1  DE2  DE3  DE4  DE1  DE2  DE3  DE1  DE2  DE3  DE4  DE5  DE6  DE1  DE2  DE3  DE4  DE1  DE2  DE3  DE1  DE2  DE3  DE1  DE2  DE1  DE2  DE3  DE1  DE2  DE3  DE1  DE2  DE3  DE1  DE2  DE3  DE4  DE1  DE2  DE3  DE4  DE1  DE2  DE3  DE4  DE5  DE1  DE2  DE3  DE4  DE1  DE2  DE3  DE1  DE2  DE3  DE4  DE5  DE1  DE2  DE3  DE4 | March 2010  Aug 2014  July 2015  Aug 2016  July 2015  Aug 2016  March 2010  June 2014  Aug 2015  Aug 2016  March 2010  July 2015  Sept 2016  Jan 2008  May 2009  March 2010  Jan 2011  Aug 2015  Sept 2016  July 2006  March 2010  July 2015  Oct 2016  Jan 2008  July 2015  Sept 2016  Jan 2008  July 2015  Sept 2016  Aug 2015  Nov 2016  June 2010  Sept 2015  Oct 2016  Oct 2012  Sept 2015  Nov 2016  May 2012  Sept 2015  Oct 2016  June 2010  Jan 2011  Aug 2015  Oct 2016  Feb 2009  March 2010  Aug 2015  Oct 2016  May 2009  March 2010  Dec 2010  Nov 2015  Sept 2016  July 2002  March 2010  Aug 2015  Sept 2016  March 2010  Sept 2015  Sept 2016  July 2002  March 2010  Jan 2011  Sept 2015  Sept 2016  March 2010  Oct 2014  Aug 2015  Sept 2016 | June 2010  Nov 2014  Apr 2016  May 2017  Apr 2016  June 2017  June 2010  Nov 2014  June 2016  May 2017  June 2010  March 2016  May 2017  May 2008  Aug 2009  June 2010  May 2011  Feb 2016  Apr 2017  Oct 2006  June 2010  Apr 2016  Apr 2017  Apr 2008  Feb 2016  May 2017  Apr 2008  Feb 2016  June 2017  Feb 2016  Apr 2017  Sept 2010  Feb 2016  Feb 2017  Apr 2013  Feb 2016  Feb 2017  Oct 2012  Jan 2016  Feb 2017  Sept 2010  Apr 2011  Jan 2016  Feb 2017  Aug 2009  Aug 2010  Feb 2016  Apr 2017  Aug 2009  Aug 2010  May 2011  Feb 2016  Apr 2017  Jan 2003  July 2010  Feb 2016  Apr 2017  June 2010  March 2016  June 2017  Nov 2002  Aug 2010  May 2011  Feb 2016  Apr 2017  June 2010  Feb 2015  March 2016  May 2017 | 3  3  9  9  9  10  3  5  10  9  3  8  8  4  3  3  4  6  7  3  3  9  6  3  7  8  3  7  9  6  5  3  5  4  6  5  3  5  4  4  3  3  5  4  6  5  6  6  3  5  5  3  7  6  4  6  7  3  6  9  4  5  4  5  7  3  4  7  8 | -3.36037  -3.96038  -13.7712  -15.5896  -12.7209  -17.6832  -3.8147  -5.67964  -13.2237  -16.6557  -4.55689  -12.1997  -17.0055  -4.18695  -2.95271  -3.22624  -4.29141  -8.22414  -10.6301  -2.70748  -4.0403  -15.6671  -11.2987  -2.72759  -11.7132  -15.8298  -3.03127  -11.7857  -13.8861  -11.6754  -9.85602  -3.28644  -10.4245  -7.5119  -5.51512  -9.82565  -6.24541  -7.62877  -7.22533  -6.20524  -4.10889  -3.68143  -8.94141  -6.23608  -6.25423  -6.07283  -9.00085  -10.1198  -3.65279  -4.5537  -5.91089  -4.95487  -9.72803  -6.71267  -5.89013  -11.0826  -12.1094  -3.88833  -9.64709  -16.5455  -4.30596  -5.13951  -4.88819  -8.34712  -10.2662  -5.6737  -4.46981  -12.5186  -16.752 |
| 3 | 3a  3b | DE1  DE2  DE3  DE1  DE2 | March 2006  March 2009  Aug 2012  Aug 2012  Nov 2013 | June 2006  July 2009  Feb 2013  Apr 2013  Apr 2014 | 3  4  6  8  5 | -3.38811  -4.39883  -9.42783  -8.28175  -7.43566 |
| 4 | 4 | DE1  DE2 | July 2006  Jan 2013 | Oct 2006  Apr 2013 | 3  3 | -1.20129  -3.1392 |
| 5 | 5a  5b  5c  5d  5e  5f  5g  5h | DE1  DE2  DE3  DE4  DE5  DE1  DE2  DE3  DE1  DE2  DE3  DE1  DE2  DE3  DE1  DE2  DE3  DE4  DE5  DE6  DE1  DE2  DE3  DE4  DE1  DE2  DE3  DE4  DE5  DE1  DE2  DE3 | Sept 2002  Aug 2003  May 2012  Oct 2015  Nov 2016  Feb 2012  July 2015  Nov 2016  Nov 2002  Nov 2014  Oct 2016  Feb 2012  July 2015  Nov 2016  Jan 2003  June 2004  June 2009  Feb 2012  Oct 2015  Nov 2016  June 2004  Oct 2008  Feb 2012  Oct 2015  March 2003  Sept 2004  Sept 2008  Feb 2012  July 2015  July 2002  Oct 2015  Nov 2016 | Apr 2003  Apr 2004  Sept 2012  Feb 2016  Feb 2017  June 2012  Feb 2016  March 2017  Feb 2003  Feb 2015  Feb 2017  May 2012  Feb 2016  Apr 2017  May 2003  Sept 2004  Sept 2009  June 2012  Feb 2016  Apr 2017  Oct 2004  Apr 2009  June 2012  Jan 2016  June 2003  Dec 2004  June 2009  June 2012  Feb 2016  Feb 2003  Feb 2016  Apr 2017 | 7  8  4  4  3  4  7  4  3  3  4  3  7  5  4  3  3  4  4  5  4  6  4  3  3  3  9  4  7  7  4  5 | -8.1326  -8.68291  -4.32673  -7.04499  -4.77067  -5.76778  -11.7249  -6.90131  -3.37482  -2.81823  -4.60395  -3.4943  -12.315  -8.66865  -3.99065  -3.47951  -3.58504  -4.00376  -6.99568  -6.4828  -5.03029  -5.95792  -3.71484  -4.58404  -3.03539  -3.57967  -9.34266  -4.26739  -5.59622  -7.99821  -6.66908  -6.19097 |
| 6 | 6a  6b  6c  6d  6e  6f  6g | DE1  DE2  DE3  DE4  DE5  DE6  DE1  DE2  DE1  DE2  DE3  DE1  DE2  DE3  DE1  DE2  DE3  DE4  DE1  DE2  DE1  DE2  DE3 | May 2003  March 2005  March 2012  Nov 2012  July 2015  Oct 2016  July 2015  Oct 2016  May 2003  July 2015  Sept 2016  March 2005  Sept 2014  Oct 2016  Nov 2002  Aug 2014  Oct 2015  Oct 2016  May 2003  July 2016  Apr 2003  Nov 2014  Aug 2016 | Oct 2003  June 2005  June 2012  May 2013  Feb 2016  March 2017  Feb 2016  Apr 2017  Sept 2003  Feb 2016  Feb 2017  June 2005  Feb 2015  Feb 2017  Feb 2003  Feb 2015  Feb 2016  Apr 2017  Sept 2003  Feb 2017  July 2003  Feb 2015  Feb 2017 | 5  3  3  6  7  5  7  6  4  7  5  3  5  4  3  6  4  6  4  7  3  3  6 | -6.38403  -2.99693  -3.80066  -6.99594  -12.3836  -8.02898  -11.7049  -9.52518  -6.4991  -6.38791  -9.95827  -2.88042  -4.92262  -8.74312  -3.03276  -5.15861  -5.5714  -6.89642  -7.52103  -12.807  -5.21334  -3.89548  -11.4277 |
| 7 | 7a  7b  7c | DE1  DE2  DE3  DE4  DE1  DE2  DE3  DE4  DE5  DE1  DE2  DE3  DE4  DE5 | Apr 2002  Apr 2003  Oct 2013  Aug 2016  Apr 2002  Apr 2003  Nov 2013  Nov 2014  Aug 2016  July 2002  March 2003  Dec 2004  Jan 2014  Sept 2016 | Sept 2002  Sept 2003  March 2014  Feb 2017  Dec 2002  Sept 2003  Apr 2014  Feb 2015  Feb 2017  Jan 2003  Apr 2004  March 2005  Apr 2014  Dec 2016 | 5  5  5  6  8  5  5  3  6  6  13  3  3  3 | -5.93807  -7.39857  -6.25334  -10.7336  -9.65784  -7.65992  -5.50612  -3.39452  -10.7387  -6.94078  -11.9268  -2.82565  -3.32252  -4.80889 |
| 8 | 8 | DE1  DE2  DE3 | June 2010  Aug 2015  Oct 2016 | Sept 2010  Feb 2016  Feb 2017 | 3  6  4 | -4.57341  -9.6502  -7.07036 |
| 9 | 9a  9b | DE1  DE2  DE3  DE4  DE1  DE2  DE3 | Jan 2010  June 2010  Aug 2015  Sept 2016  Jan 2010  Aug 2015  Sept 2016 | Apr 2010  Sept 2010  Feb 2016  Feb 2017  Apr 2010  Jan 2016  Feb 2017 | 3  3  6  5  3  5  5 | -3.26735  -4.59533  -8.97217  -8.12096  -4.47946  -7.88955  -6.97581 |
| 10 | 10a  10b  10c | DE1  DE2  DE1  DE2  DE3  DE1  DE2 | July 2015  Nov 2016  Jan 2010  Aug 2015  Oct 2016  July 2015  Oct 2016 | Feb 2016  March 2017  Apr 2010  Feb 2016  March 2017  Feb 2016  Feb 2017 | 7  4  3  6  5  7  4 | -10.2273  -6.41944  -3.87441  -9.49296  -8.2219  -9.41724  -6.17063 |
| 11 | 11a  11b | DE1  DE2  DE3  DE1  DE2  DE3  DE4 | Nov 2013  Nov 2014  Aug 2016  Sept 2014  Jan 2016  Aug 2016  Jan 2017 | Feb 2014  Feb 2015  Feb 2017  Feb 2015  Apr 2016  Nov 2016  Apr 2017 | 3  3  6  5  3  3  3 | -2.59358  -4.43655  -10.1997  -6.53212  -3.42546  -6.11126  -4.82947 |
| 12 | 12a  12b | DE1  DE2  DE3  DE1  DE2  DE3  DE4 | March 2010  Aug 2015  Nov 2016  Oct 2002  March 2010  July 2015  Nov 2016 | July 2010  Feb 2016  March 2017  May 2003  June 2010  Feb 2016  March 2017 | 4  6  4  7  3  6  4 | -6.16075  -11.3846  -9.78872  -9.31585  -4.09391  -11.5008  -8.42836 |
| 13 | 13a  13b | DE1  DE2  DE3  DE4  DE1  DE2  DE3 | July 2002  March 2010  Aug 2015  Nov 2016  July 2002  Aug 2015  Nov 2016 | Dec 2002  June 2010  Feb 2016  Apr 2017  May 2003  Feb 2016  March 2017 | 5  4  5  5  10  5  4 | -6.20568  -4.96857  -10.768  -10.2655  -14.7505  -8.25692  -7.02427 |
| 14 | 14a  14b  14c | DE1  DE2  DE3  DE4  DE5  DE1  DE2  DE3  DE1  DE2  DE3  DE4 | June 2007  Feb 2009  March 2010  Nov 2015  Sept 2016  Aug 2002  Dec 2008  Sept 2015  Oct 2002  Nov 2003  Aug 2015  Nov 2016 | Feb 2008  June 2009  June 2010  Feb 2016  Jan 2017  Jan 2003  Apr 2009  Feb 2016  May 2003  May 2004  Feb 2016  Feb 2017 | 8  4  3  3  4  5  4  5  7  6  6  3 | -6.89161  -4.1396  -3.01611  -5.03306  -5.31994  -3.14045  -2.90346  -6.69123  -12.8709  -6.69003  -9.22507  -4.46634 |
| 15 | 15a  15b  15c | DE1  DE2  DE3  DE4  DE5  DE1  DE2  DE3  DE4  DE1  DE2  DE3 | Dec 2002  March 2005  May 2008  Sept 2015  Dec 2002  Dec 2003  May 2008  Oct 2015  Sept 2002  Sept 2003  Oct 2015  Nov 2016 | March 2003  June 2005  Aug 2008  Feb 2016  March 2003  Apr 2004  Aug 2008  Feb 2016  May 2003  Apr 2004  Feb 2016  Feb 2017 | 3  3  3  5  3  4  3  4  8  7  4  3 | -3.021  -2.9754  -3.52321  -6.36403  -4.46517  -4.97852  -3.87259  -5.16407  -12.8708  -7.52307  -6.47997  -4.6409 |
| 16 | 16a  16b | DE1  DE2  DE3  DE4  DE1  DE2 | Sept 2002  Oct 2002  July 2015  Nov 2016  July 2015  Oct 2016 | Apr 2003  March 2004  Oct 2015  March 2017  Jan 2016  Feb 2017 | 7  5  3  4  6  4 | -10.4333  -5.71243  -4.83975  -6.0301  -9.00419  -6.22749 |
| 17 | 17a  17b  17c | DE1  DE2  DE1  DE2  DE3  DE4  DE1  DE2  DE3 | Nov 2013  July 2016  May 2003  Oct 2012  Nov 2013  July 2016  Sept 2012  Dec 2013  July 2016 | March 2014  Feb 2017  Sept 2003  Jan 2013  Apr 2014  Feb 2017  Jan 2013  Apr 2014  Feb 2017 | 4  7  4  3  5  7  4  4  7 | -4.22331  -10.0331  -5.43942  -3.46715  -6.19622  -12.356  -4.39119  -4.67454  -10.7703 |
| 18 | 18a  18b | DE1  DE1  DE2 | Nov 2016  May 2005  Oct 2016 | Feb 2017  Aug 2005  Jan 2017 | 3  3  3 | -4.45643  -3.74682  -3.5428 |
| 19 | 19 | DE1  DE2  DE3 | Nov 2002  Aug 2014  Oct 2016 | Feb 2003  Feb 2015  Feb 2017 | 3  6  4 | -3.1779  -5.31757  -5.17883 |
| 20 | 20 | DE1  DE2 | Aug 2014  Jan 2017 | March 2015  Apr 2017 | 7  3 | -6.3178  -3.37003 |
| 21 | 21a  21b | DE1  DE2  DE3  DE4  DE5  DE6  DE1  DE2  DE3  DE4  DE5 | June 2002  Aug 2003  Nov 2013  Nov 2014  Aug 2016  Jan 2017  Apr 2002  Apr 2003  Nov 2013  Nov 2014  Aug 2016 | Sept 2002  Dec 2003  Apr 2014  Feb 2015  Nov 2017  Apr 2017  Jan 2003  Dec 2003  Apr 2014  Feb 2015  Dec 2016 | 3  4  5  3  3  3  9  8  5  3  4 | -5.29938  -4.75514  -5.25175  -3.90352  -5.32093  -3.62502  -10.5874  -10.7572  -5.0073  -3.3428  -6.20099 |
| 22 | 22a  22b | DE1  DE2  DE3  DE4  DE1  DE2  DE3  DE4 | July 2002  Aug 2003  Sept 2013  Aug 2016  July 2002  July 2003  Jan 2014  Aug 2016 | Jan 2003  Apr 2004  Apr 2014  Dec 2016  Jan 2003  Dec 2003  Apr 2014  Feb 2017 | 6  8  7  4  6  5  3  6 | -6.04228  -0.58006  -7.00225  -5.30243  -5.72429  -5.6195  -3.1782  -7.57356 |
| 23 | 23a  23b  23c  23d  23e  23f | DE1  DE2  DE3  DE4  DE1  DE2  DE3  DE4  DE5  DE6  DE7  DE1  DE2  DE3  DE4  DE5  DE6  DE1  DE2  DE3  DE4  DE5  DE1  DE2  DE3  DE1  DE2  DE3  DE4 | Aug 2009  Oct 2014  Aug 2015  Nov 2016  July 2002  Nov 2004  Sept 2009  March 2010  Dec 2014  Aug 2015  Nov 2016  July 2002  Nov 2004  Sept 2009  March 2010  Aug 2015  Nov 2016  July 2002  Sept 2009  Oct 2014  Aug 2015  Nov 2016  July 2002  July 2015  Nov 2016  Sept 2002  Nov 2003  Oct 2015  Nov 2016 | June 2010  Feb 2015  March 2016  Apr 2017  May 2003  Apr 2005  Jan 2010  June 2010  March 2015  Feb 2016  Apr 2017  May 2003  May 2005  Jan 2010  June 2010  Feb 2016  March 2017  June 2003  Dec 2009  Feb 2015  Feb 2016  Apr 2017  June 2003  Feb 2016  March 2017  May 2003  Apr 2004  Feb 2016  Feb 2017 | 10  4  7  5  10  5  4  3  3  6  5  10  6  4  3  6  4  11  3  4  6  5  11  7  4  8  5  4  3 | -11.9381  -5.31625  -9.9202  -11.3636  -10.203  -4.77535  -3.45547  -3.73486  -3.61124  -5.63669  -7.08703  -12.9316  -5.84703  -3.9497  -4.07851  -6.62815  -6.63629  -11.1232  -3.0491  -4.25087  -8.29978  -9.32857  -13.9222  -7.10948  -7.78225  -13.9231  -6.21497  -5.83897  -4.25608 |
| 24 | 24 | DE1  DE2  DE3  DE4 | Oct 2009  Oct 2014  Aug 2015  Sept 2016 | May 2010  Feb 2015  Feb 2016  May 2017 | 7  4  6  8 | -7.26686  -4.70927  -8.53341  -16.8877 |
| 25 | 25 | DE1  DE2  DE3  DE4  DE5 | Apr 2002  Aug 2003  Feb 2008  Apr 2014  Jan 2016 | Feb 2003  July 2004  May 2008  Aug 2014  Sept 2016 | 10  11  3  4  8 | -10.8656  -9.65287  -3.34756  -3.01211  -6.64137 |

**Table S3.** Drought Events obtained through GRACE-DSI over major sub-basins of India. DE represents drought events. We have named sub-basins with notation 1a,1b…etc. Detailed naming and notation of basins and sub-basins are presented in Appendix 1. The drought duration and drought severity of each event are listed in the table. Drought duration is the period of time where the GRACE-DSI value is below the fixed threshold value (for this study, we have taken threshold value =-0.5). Drought Severity is the cumulative value of GRACE-DSI within the drought duration.

| Basin | Sub-basins | Drought events | Start month | End month | Drought duration  (months) | Drought Severity |
| --- | --- | --- | --- | --- | --- | --- |
| 1 | 1a  1b  1c  1d  1e  1f  1g  1h  1i  1j  1k | DE1  DE2  DE3  DE4  DE5  DE6  DE7  DE1  DE2  DE3  DE4  DE5  DE6  DE7  DE1  DE2  DE3  DE4  DE1  DE2  DE3  DE4  DE5  DE6  DE7  DE8  DE1  DE2  DE3  DE4  DE5  DE6  DE1  DE2  DE3  DE4  DE1  DE2  DE3  DE4  DE1  DE2  DE3  DE4  DE1  DE2  DE3  DE4  DE5  DE1  DE2  DE3  DE4  DE5  DE1  DE2  DE3  DE4  DE5 | July 2002  Jan 2004  Nov 2007  July 2008  Apr 2015  March 2016  March 2017  July 2002  Jan 2004  Nov 2007  July 2008  June 2015  March2016  March2017  Dec 2009  Dec 2011  Apr 2014  May 2016  Oct 2008  Aug 2009  Dec 2011  Oct 2012  Aug 2013  July 2014  May 2016  Feb 2017  Sept 2008  Aug 2009  Jan 2012  Aug 2013  May 2016  Jan 2017  Aug 2009  Dec 2011  July 2014  May 2016  Aug 2009  Dec 2011  July 2014  May 2016  Dec 2009  Dec 2011  Apr 2014  May 2016  Jan 2010  May 2012  Oct 2012  July 2014  July 2015  May 2012  Oct 2012  Apr 2013  Feb 2014  July 2015  May 2012  Oct 2012  Apr 2013  June 2014  July 2015 | March 2003  Apr 2004  March 2008  Feb 2009  Oct 2015  Aug 2016  June 2017  March 2003  Apr 2004  March 2008  Feb 2009  Oct 2015  Aug 2016  June 2017  May 2010  Feb 2014  Nov 2014  June 2017  Jan 2009  Apr 2010  Apr 2012  Feb 2013  Dec 2013  Nov 2014  Sept 2016  June 2017  Jan 2009  Apr 2010  Feb 2013  Dec 2013  Sept 2016  June 2017  May 2010  Dec 2013  Oct 2014  June 2017  May 2010  Dec 2013  Nov 2014  June 2017  May 2010  Feb 2014  Nov 2014  June 2017  June 2010  Aug 2012  Dec 2013  Nov 2014  June 2017  Aug 2012  Jan 2013  Dec 2013  Feb 2015  June 2017  Aug 2012  Jan 2013  Dec 2013  Feb 2015  June 2017 | 8  3  4  7  6  5  3  8  3  4  7  4  5  3  5  26  7  13  3  8  4  4  4  4  4  4  4  8  13  4  4  5  9  24  3  13  9  24  4  13  5  26  7  13  5  3  14  4  23  3  3  8  12  23  3  3  8  8  23 | -9.44722  -3.30337  -4.3933  -9.45898  -5.70409  -8.99444  -5.94077  -9.59357  -3.26322  -4.34076  -9.38495  -5.02905  -9.05068  -5.8155  -4.96018  -31.5826  -8.57722  -20.1643  -3.24182  -9.98544  -4.9241  -5.63587  -5.42817  -7.30165  -8.49376  -7.39527  -4.61277  -8.18465  -15.2724  -5.62252  -7.20514  -12.2819  -7.66814  -29.6241  -4.34688  -20.2409  -7.63545  -29.686  -4.89352  -20.1677  -4.69269  -29.2924  -8.18183  -21.0053  -3.63218  -3.60416  -12.073  -5.58417  -36.8563  -2.96927  -2.41329  -5.35365  -9.99701  -39.2301  -2.96002  -2.38623  -5.16271  -7.28305  -39.7965 |
| 2 | 2a  2b  2c  2d  2e  2f  2g  2h  2i  2j  2k  2l  2m  2n  2o  2p  2q  2r  2s | DE1  DE2  DE3  DE4  DE5  DE1  DE2  DE3  DE1  DE2  DE3  DE4  DE5  DE1  DE2  DE3  DE4  DE5  DE1  DE2  DE3  DE4  DE5  DE1  DE2  DE3  DE4  DE1  DE2  DE3  DE4  DE1  DE2  DE3  DE4  DE1  DE2  DE3  DE4  DE5  DE6  DE1  DE2  DE3  DE4  DE5  DE6  DE7  DE1  DE2  DE3  DE4  DE5  DE6  DE7  DE1  DE2  DE3  DE4  DE5  DE6  DE7  DE8  DE1  DE2  DE3  DE4  DE5  DE6  DE7  DE1  DE2  DE3  DE4  DE5  DE1  DE2  DE3  DE4  DE5  DE6  DE1  DE2  DE3  DE4  DE5  DE1  DE2  DE3  DE4  DE1  DE2  DE3  DE4  DE1  DE2  DE3  DE4  DE5  DE6 | Apr 2010  May 2012  Oct 2012  July 2014  July 2015  Apr 2010  July 2014  July 2015  Apr 2010  May 2012  Oct 2012  July 2014  July 2015  Apr 2010  May 2012  Aug 2014  July 2015  May 2016  May 2009  March 2010  Jan 2011  Aug 2015  Aug 2016  Apr 2010  Aug 2014  July 2015  May 2016  Apr 2010  Aug 2014  July 2015  June 2016  Apr 2010  Sept 2014  July 2015  June 2016  Apr 2010  Dec 2010  March 2013  Aug 2014  July 2015  July 2016  Apr 2009  March 2010  Jan 2011  March 2013  Aug 2014  July 2015  July 2016  March 2010  Feb 2011  March 2012  Oct 2012  Aug 2014  July 2015  May 2016  Apr 2009  March 2010  Jan 2011  March 2012  Feb 2013  Aug 2014  July 2015  June 2016  Apr 2009  March 2010  Jan 2011  March 2013  Aug 2014  July 2015  July 2016  Apr 2010  Oct 2010  Sept 2014  July 2015  Aug 2016  May 2009  May 2010  Dec 2010  Sept 2014  Aug 2015  Aug 2016  Aug 2002  Feb 2003  March 2010  July 2015  Aug 2016  March 2010  Aug 2014  July 2015  June 2016  May 2009  Feb 2010  Aug 2015  Aug 2016  March 2010  May 2012  March 2013  Sept 2014  July 2015  June 2016 | July 2010  Aug 2012  Jan 2013  Nov 2014  June 2017  July 2010  Feb 2015  June 2017  July 2010  Aug 2012  Jan 2013  Nov 2014  June 2017  July 2010  Aug 2012  Feb 2015  March 2016  June 2017  Aug 2009  Aug 2010  May 2011  Feb 2016  Apr 2017  July 2010  Dec 2014  March 2016  June 2017  July 2010  Dec 2014  March 2016  June 2017  Aug 2010  Dec 2014  March 2016  June 2017  July 2010  May 2011  June 2013  Jan 2015  March 2016  Apr 2017  July 2009  Aug 2010  May 2011  June 2013  Nov 2014  Feb 2016  March 2017  July 2010  May 2011  Aug 2012  June 2013  Nov 2014  Feb 2016  June 2017  July 2009  Aug 2010  May 2011  Aug 2012  June 2013  Nov 2014  Feb 2016  March 2017  July 2009  Aug 2010  May 2011  June 2013  Nov 2014  Feb 2016  March 2017  Aug 2010  May 2011  Dec 2014  Feb 2016  Apr 2017  Aug 2009  Aug 2010  May 2011  Dec 2014  Feb 2016  Apr 2017  Dec 2002  May 2003  July 2010  Feb 2016  March 2017  Aug 2010  Nov 2014  Feb 2016  June 2017  Oct 2009  Sept 2010  Feb 2016  Apr 2017  July 2010  Aug 2012  June 2013  Feb 2015  March 2016  June 2017 | 3  3  3  4  23  3  7  23  3  3  3  4  23  3  3  6  8  13  3  5  4  6  8  3  4  8  13  3  4  8  12  4  3  8  12  3  5  3  5  8  9  3  5  4  3  3  7  8  4  3  5  8  3  7  13  3  5  4  5  4  3  7  9  3  5  4  3  3  7  8  4  7  3  7  8  3  3  5  3  6  8  4  3  4  7  7  5  3  7  12  5  7  6  8  4  3  3  5  8  12 | -2.59478  -2.87018  -2.32542  -5.03031  -41.9882  -2.8354  -6.58792  -42.4152  -2.5974  -2.89963  -2.31426  -5.01744  -41.4251  -4.18048  -3.1702  -3.92852  -13.9079  -26.2394  -4.04233  -6.51412  -4.23967  -12.4382  -17.9231  -4.268  -5.014  -17.0549  -26.2462  -4.49888  -4.96167  -15.7875  -22.5267  -5.06259  -4.0562  -16.708  -23.0172  -5.79283  -5.20847  -3.46527  -5.90317  -17.2992  -17.0351  -3.49898  -6.00012  -5.18933  -4.88266  -4.83195  -16.2588  -15.3521  -4.56787  -3.17317  -5.55095  -10.0908  -4.37474  -15.4384  -19.4093  -3.42404  -4.24394  -5.18064  -5.13747  -6.42025  -4.87134  -14.4315  -14.3002  -3.77992  -5.40775  -5.79659  -5.2015  -5.07045  -14.7329  -13.7761  -5.87386  -7.43611  -4.85301  -14.0602  -15.6606  -3.67774  -4.73013  -5.88882  -4.70541  -11.3404  -14.6648  -4.01549  -4.13512  -6.64129  -12.077  -15.0682  -6.18965  -3.7202  -13.5656  -24.8833  -5.21981  -8.59085  -10.8959  -16.448  -6.18646  -3.54093  -2.98827  -6.34673  -14.3895  -25.5112 |
| 3 | 3a  3b | DE1  DE2  DE3  DE4  DE5  DE6  DE7  DE1  DE2  DE3  DE4  DE5  DE6 | Jan 2010  Dec 2011  Oct 2012  Aug 2013  July 2014  July 2015  May 2016  Jan 2011  Oct 2012  Aug 2013  July 2014  July 2015  May 2016 | Apr 2010  Apr 2010  June 2013  Apr 2014  Jan 2015  Nov 2015  Nov 2016  May 2011  June 2013  March 2014  Jan 2015  Nov 2015  Nov 2016 | 3  4  8  8  6  4  6  4  8  7  6  4  6 | -4.06921  -4.42648  -10.4947  -8.62238  -11.2977  -8.82935  -10.986  -4.48812  -8.86426  -9.38495  -9.82359  -9.32693  -12.0759 |
| 4 | 4 | DE1  DE2  DE3  DE4  DE5  DE6 | Jan 2003  Jan 2006  Apr 2009  Aug 2014  July 2015  June 2016 | May 2003  May 2006  July 2009  Jan 2015  Nov 2015  Oct 2016 | 4  4  3  5  4  4 | -3.43303  -4.39156  -3.61743  -11.8203  -10.0172  -6.69903 |
| 5 | 5a  5b  5c  5d  5e  5f  5g  5h | DE1  DE2  DE3  DE4  DE5  DE6  DE1  DE2  DE3  DE4  DE5  DE1  DE2  DE3  DE4  DE5  DE6  DE7  DE1  DE2  DE3  DE4  DE5  DE6  DE7  DE1  DE2  DE3  DE4  DE5  DE6  DE7  DE8  DE1  DE2  DE3  DE4  DE5  DE6  DE7  DE1  DE2  DE3  DE4  DE5  DE6  DE7  DE1  DE2  DE3  DE4  DE5  DE6  DE7 | Apr 2002  Nov 2002  Feb 2004  March 2005  July 2015  Aug 2016  March 2004  March 2005  Feb 2013  July 2015  Aug 2016  July 2002  Jan 2005  Apr 2009  Jan 2010  Sept 2014  Aug 2015  Aug 2016  March 2005  May 2009  March 2012  March 2013  Aug 2014  July 2015  Aug 2016  Jan 2003  Jan 2005  Apr 2009  March 2010  March 2013  Sept 2014  July 2015  Aug 2016  Jan 2003  Sept 2004  May 2008  Nov 2008  Feb 2010  Aug 2015  Sept 2016  Jan 2003  Sept 2004  May 2008  Nov 2008  Feb 2010  Aug 2015  Sept 2016  July 2002  Feb 2005  March 2009  Jan 2010  Sept 2014  Aug 2015  Aug 2016 | Aug 2002  Sept 2003  July 2004  June 2005  Feb 2016  Feb 2017  July 2004  June 2005  May 2013  Feb 2016  March 2017  July 2003  Aug 2005  Sept 2009  May 2010  Feb 2015  Feb 2016  March 2017  June 2005  Aug 2009  Aug 2012  May 2013  Feb 2015  Feb 2016  March 2017  July 2003  July 2005  Aug 2009  June 2010  July 2013  Feb 2015  Feb 2016  March 2017  May 2003  June 2005  Sept 2008  Oct 2009  July 2010  Feb 2016  Jan 2017  May 2003  June 2005  Sept 2008  Oct 2009  July 2010  Feb 2016  Jan 2017  July 2003  July 2005  Aug 2009  June 2010  Feb 2015  Feb 2016  March 2017 | 4  10  5  3  7  6  4  3  3  7  7  12  7  5  4  5  6  7  3  3  5  3  6  7  7  6  6  4  3  4  5  7  7  4  9  4  11  5  6  4  4  9  4  11  5  6  4  12  5  5  5  5  6  7 | -5.24541  -9.98313  -7.06714  -3.31395  -14.7528  -9.28634  -4.29689  -3.74974  -3.41639  -16.1339  -11.7334  -16.334  -7.92202  -4.39242  -4.13143  -5.40023  -11.1725  -9.92539  -4.78306  -3.17173  -4.73934  -3.79339  -7.38502  -16.0616  -13.8717  -6.50325  -7.86275  -4.04707  -3.06081  -3.28887  -7.06951  -13.3815  -12.5231  -4.93606  -8.71554  -3.42861  -11.7737  -3.68099  -8.28225  -7.38663  -4.99898  -8.64248  -3.19393  -11.4704  -3.91844  -8.48978  -7.63217  -15.1424  -5.94795  -4.59923  -4.80305  -5.7776  -12.1021  -10.9844 |
| 6 | 6a  6b  6c  6d  6e  6f  6g | DE1  DE2  DE3  DE4  DE5  DE1  DE2  DE3  DE4  DE5  DE6  DE7  DE1  DE2  DE3  DE4  DE5  DE6  DE7  DE1  DE2  DE3  DE4  DE5  DE6  DE7  DE1  DE2  DE3  DE4  DE5  DE6  DE1  DE2  DE3  DE4  DE5  DE6  DE7  DE8  DE1  DE2  DE3  DE4  DE5  DE6  DE7  DE8 | Feb 2004  March 2005  Oct 2012  July 2015  July 2016  March 2005  May 2009  March 2012  March 2013  Aug 2014  July 2015  Aug 2016  March 2003  Feb 2004  March 2005  May 2012  Feb 2013  July 2015  July 2016  March 2003  Jan 2005  May 2012  Feb 2013  Sept 2014  July 2015  July 2016  Nov 2002  Jan 2005  Apr 2009  Sept 2014  Aug 2015  Aug 2016  March 2003  Feb 2004  March 2005  May 2012  Feb 2013  Oct 2014  Aug 2015  June 2016  March 2003  Feb 2004  March 2005  May 2012  Feb 2013  Oct 2014  Aug 2015  June 2016 | May 2004  June 2005  June 2013  Feb 2016  March 2017  June 2005  Aug 2009  Aug 2012  June 2013  Feb 2015  Feb 2016  March 2017  Aug 2003  May 2004  June 2005  Aug 2012  June 2013  Feb 2016  March 2017  Aug 2003  July 2005  Aug 2012  June 2013  Feb 2015  Feb 2016  March 2017  July 2003  Aug 2005  Aug 2009  Feb 2015  Feb 2016  March 2017  Sept 2003  May 2004  June 2005  Aug 2012  July 2013  Feb 2015  Feb 2016  Feb 2017  Sept 2003  May 2004  June 2005  Aug 2012  July 2013  Feb 2015  Feb 2016  Feb 2017 | 3  3  8  7  8  3  3  5  3  6  7  7  5  3  3  3  4  7  8  5  6  3  4  5  7  8  8  7  4  5  6  7  6  3  3  3  5  4  6  8  6  3  3  3  5  4  6  8 | -4.18222  -3.28811  -9.14576  -17.4943  -13.5103  -4.84324  -3.27323  -4.83313  -3.62552  -7.59377  -15.8087  -13.8507  -4.37052  -4.68465  -4.4948  -2.85423  -5.4481  -14.1663  -14.7861  -5.2561  -7.98196  -4.29018  -4.67551  -7.00138  -11.726  -16.0145  -9.47351  -8.93956  -3.8563  -6.98732  -11.254  -11.8894  -7.42909  -5.01249  -5.1942  -3.36931  -5.77345  -5.30611  -9.00677  -16.7553  -7.4033  -4.96715  -5.24461  -3.36959  -5.75489  -5.34162  -9.16783  -16.6421 |
| 7 | 7a  7b  7c | DE1  DE2  DE3  DE4  DE5  DE6  DE7  DE8  DE9  DE1  DE2  DE3  DE4  DE5  DE6  DE7  DE8  DE1  DE2  DE3  DE4  DE5  DE6  DE7  DE8 | Apr 2002  Apr 2003  Feb 2004  March 2005  Dec 2012  Nov 2013  Nov 2014  Oct 2015  June 2016  Apr 2003  Feb 2004  March 2005  May 2012  March 2013  Nov 2013  Nov 2014  June 2016  Apr 2003  Feb 2004  Feb 2005  May 2012  March 2013  Dec 2013  Nov 2014  June 2016 | Aug 2002  Dec 2003  May 2004  June 2005  July 2013  Apr 2014  Feb 2015  Jan 2016  Feb 2017  Nov 2003  May 2004  Aug 2005  Sept 2012  July 2013  Apr 2014  Feb 2015  March 2017  Nov 2003  May 2004  Aug 2005  Sept 2012  July 2013  Apr 2014  Feb 2015  March 2017 | 4  8  3  3  7  5  3  3  8  7  3  5  4  4  5  3  9  7  3  6  4  4  4  3  9 | -4.10219  -8.4507  -6.04509  -2.82977  -7.70174  -7.12144  -3.33449  -3.3812  -19.1167  -8.28157  -5.66988  -4.99167  -4.24983  -5.80099  -6.31757  -4.13943  -19.6984  -8.87994  -5.40487  -6.82058  -4.81137  -4.84499  -4.9136  -4.66217  -19.1502 |
| 8 | 8 | DE1  DE2  DE3  DE4  DE5  DE6  DE7 | Apr 2009  March 2010  Jan 2011  March 2013  Aug 2014  July 2015  July 2016 | July 2009  Aug 2010  May 2011  June 2013  Nov 2014  Feb 2016  March 2017 | 3  5  4  3  3  7  8 | -3.48018  -6.14065  -5.03697  -4.25577  -4.75133  -16.7006  -15.1085 |
| 9 | 9a  9b | DE1  DE2  DE3  DE4  DE5  DE1  DE2  DE3  DE4  DE5  DE6 | Apr 2009  March 2010  March 2013  July 2015  July 2016  Feb 2003  Apr 2009  March 2010  March 2013  July 2015  July 2016 | July 2009  Aug 2010  June 2013  Feb 2016  March 2017  May 2003  July 2009  Aug 2010  June 2013  Feb 2016  Feb 2017 | 3  5  3  7  8  3  3  5  3  7  7 | -3.14635  -6.11449  -4.41559  -17.1265  -14.8821  -3.97772  -2.69396  -5.26867  -3.9138  -16.7898  -14.0167 |
| 10 | 10a  10b  10c | DE1  DE2  DE3  DE4  DE5  DE6  DE7  DE8  DE1  DE2  DE3  DE4  DE5  DE6  DE1  DE2  DE3  DE4  DE5 | Apr 2002  Dec 2002  March 2005  May 2009  March 2010  Sept 2014  Aug 2015  Aug 2016  Jan 2003  March 2009  March 2010  Sept 2014  July 2015  Aug 2016  Dec 2002  Apr 2009  March 2010  July 2015  Aug 2016 | Aug 2002  July 2003  July 2005  Aug 2009  July 2010  Dec 2014  Feb 2016  Jan 2017  July 2003  July 2009  Aug 2010  Dec 2014  Feb 2016  March 2017  July 2003  July 2009  Aug 2010  Feb 2016  Feb 2017 | 4  7  4  3  4  3  6  5  6  4  5  3  7  7  7  3  5  7  6 | -4.36355  -10.3477  -3.66813  -3.15733  -4.76239  -4.40209  -13.6328  -11.7737  -6.1529  -3.9123  -5.85197  -4.22841  -15.6706  -14.1043  -8.10374  -2.97815  -5.36447  -16.2644  -13.0088 |
| 11 | 11a  11b | DE1  DE2  DE3  DE4  DE5  DE6  DE7  DE1  DE2  DE3  DE4  DE5  DE6  DE7 | Dec 2002  Feb 2004  May 2012  March 2013  Sept 2014  Aug 2015  June 2016  March 2003  Jan 2005  May 2012  March 2013  Sept 2014  Oct 2015  July 2016 | July 2003  May 2004  Aug 2012  July 2013  March 2015  Feb 2016  March 2017  Sept 2003  Aug 2005  Sept 2012  July 2013  March 2015  Feb 2016  Apr 2017 | 7  3  3  4  6  6  9  6  7  4  4  6  4  9 | -7.37419  -4.56187  -3.61046  -5.71874  -6.7099  -9.53489  -16.8455  -6.41582  -11.3079  -6.41451  -4.99125  -9.10836  -5.56635  -16.6328 |
| 12 | 12a  12b | DE1  DE2  DE3  DE4  DE5  DE6  DE1  DE2  DE3  DE4  DE5 | Aug 2002  Feb 2003  March 2010  March 2013  July 2015  Aug 2016  Apr 2002  Apr 2004  March 2010  July 2015  Aug 2016 | Dec 2002  May 2003  July 2010  June 2013  Feb 2016  March 2017  July 2003  July 2004  July 2010  Feb 2016  March 2017 | 4  3  4  3  7  7  15  3  4  7  7 | -4.01549  -4.13512  -6.64129  -2.76223  -12.077  -15.0682  -17.2861  -3.50073  -4.21425  -11.7074  -12.865 |
| 13 | 13a  13b | DE1  DE2  DE3  DE4  DE5  DE6  DE1  DE2  DE3  DE4  DE5  DE6  DE7  DE8 | Aug 2002  Feb 2003  March 2010  March 2013  July 2015  Aug 2016  Apr 2002  Apr 2004  March 2005  March 2006  March 2010  Sept 2014  Aug 2015  Aug 2016 | Dec 2002  May 2003  July 2010  June 2013  Feb 2016  March 2017  July 2003  Sept 2004  Sept 2005  June 2006  July 2010  Feb 2015  Feb 2016  March 2017 | 4  3  4  3  7  7  15  5  6  3  4  5  6  7 | -4.01549  -4.13512  -6.64129  -2.76223  -12.077  -15.0682  -19.5338  -5.19504  -6.26858  -3.1858  -3.91873  -4.46765  -7.93837  -11.238 |
| 14 | 14a  14b  14c | DE1  DE2  DE3  DE4  DE5  DE6  DE1  DE2  DE3  DE4  DE1  DE2  DE3  DE4  DE5 | March 2003  May 2009  March 2010  Sept 2014  Aug 2015  Sept 2016  Apr 2002  Nov 2002  July 2015  Sept 2016  Apr 2002  Nov 2003  March 2005  Aug 2015  Aug 2016 | July 2003  Aug 2009  Sept 2010  Dec 2014  Feb 2016  March 2017  Aug 2002  May 2003  Feb 2016  Jan 2017  Aug 2003  Sept 2004  June 2005  Feb 2016  Feb 2017 | 4  3  6  3  6  6  4  6  7  4  16  10  3  6  6 | -3.81366  -3.9341  -5.92003  -3.3244  -10.2167  -12.6102  -4.31386  -7.87263  -12.4293  -7.16235  -25.3915  -11.3698  -3.96737  -9.47999  -7.65142 |
| 15 | 15a  15b  15c | DE1  DE2  DE3  DE4  DE5  DE6  DE1  DE2  DE3  DE4  DE5  DE6  DE1  DE2  DE3  DE4  DE5 | Apr 2002  Nov 2002  March 2005  Nov 2008  Aug 2015  Sept 2016  Apr 2002  Nov 2002  Feb 2004  March 2005  July 2015  Sept 2016  Apr 2002  Jan 2004  March 2005  Aug 2015  Aug 2016 | Aug 2002  May 2003  June 2005  Sept 2009  Feb 2016  Jan 2017  Aug 2002  Sept 2003  July 2004  June 2005  Feb 2016  Jan 2017  Aug 2003  Sept 2004  June 2005  Feb 2016  Feb 2017 | 4  6  3  10  6  4  4  10  5  3  7  4  16  8  3  6  6 | -4.41432  -7.66649  -2.83809  -9.70819  -10.5881  -7.35035  -5.34713  -12.2409  -6.80596  -2.7849  -12.1371  -5.69524  -25.3915  -11.3698  -3.96737  -9.47999  -7.65142 |
| 16 | 16a  16b | DE1  DE2  DE3  DE4  DE5  DE1  DE2  DE3  DE4  DE5  DE6 | Apr 2002  Jan 2004  March 2005  July 2015  Aug 2016  Apr 2003  Feb 2004  March 2005  March 2013  July 2015  Aug 2016 | Aug 2003  Aug 2004  June 2005  Feb 2016  Feb 2017  Aug 2003  May 2004  June 2005  June 2013  Jan 2016  Feb 2017 | 16  7  3  7  6  4  3  3  3  6  6 | -21.2438  -10.6104  -4.04778  -12.7498  -9.4454  -3.88381  -4.37769  -4.11785  -3.6662  -12.7118  -12.4217 |
| 17 | 17a  17b  17c | DE1  DE2  DE3  DE4  DE5  DE6  DE7  DE8  DE9  DE1  DE2  DE3  DE4  DE5  DE6  DE7  DE8  DE9  DE1  DE2  DE3  DE4 | May 2002  Apr 2003  Feb 2004  March 2005  March 2013  Nov 2013  Nov 2014  Oct 2015  June 2016  Apr 2002  Apr 2003  Feb 2004  March 2005  Dec 2012  Nov 2013  Nov 2014  Oct 2015  June 2016  May 2012  Nov 2012  Nov 2014  Apr 2016 | Aug 2002  Dec 2003  May 2004  June 2005  July 2013  Apr 2014  Feb 2015  Jan 2016  Feb 2017  Aug 2002  Dec 2003  May 2004  June 2005  July 2013  Apr 2014  Feb 2015  Jan 2016  Feb 2017  Sept 2012  Apr 2014  Feb 2015  March 2017 | 3  8  3  3  4  5  3  3  8  4  8  3  3  7  5  3  3  8  4  17  3  11 | -3.12861  -8.48715  -5.93093  -3.62438  -5.26802  -6.70321  -3.79532  -4.27401  -18.1786  -4.10219  -8.4507  -6.04509  -2.82977  -7.70174  -7.12144  -3.33449  -3.3812  -19.1167  -5.0249  -21.0177  -3.24142  -24.9815 |
| 18 | 18a  18b | DE1  DE2  DE3  DE4  DE5  DE6  DE1  DE2  DE3  DE4  DE5  DE6 | Apr 2002  Nov 2002  Apr 2009  March 2010  July 2015  Aug 2016  Apr 2002  March 2005  Apr 2009  Jan 2010  Aug 2015  Aug 2016 | Sept 2002  July 2003  July 2009  June 2010  Feb 2016  Feb 2017  Aug 2003  July 2005  July 2009  June 2010  Feb 2016  Jan 2017 | 5  8  3  3  7  6  16  4  3  5  6  5 | -4.90592  -11.2347  -2.79015  -3.55618  -15.9038  -11.8175  -25.0378  -4.0269  -2.64621  -4.11667  -12.0044  -7.63463 |
| 19 | 19 | DE1  DE2  DE3  DE4  DE5  DE6  DE7 | July 2002  Jan 2005  Apr 2009  Jan 2010  Sept 2014  Aug 2015  Aug 2016 | July 2003  Aug 2005  Sept 2009  May 2010  Feb 2015  Feb 2016  March 2017 | 12  7  5  4  5  6  7 | -13.9258  -8.92976  -4.67217  -4.27231  -6.52803  -10.5761  -10.6287 |
| 20 | 20 | DE1  DE2  DE3  DE4  DE5  DE6  DE7 | March 2003  Jan 2005  May 2012  March 2013  Sept 2014  Oct 2015  July 2016 | Aug 2003  Aug 2005  Sept 2012  July 2013  March 2015  Feb 2016  Apr 2017 | 5  7  4  4  6  4  9 | -6.41582  -11.3079  -6.41451  -4.99125  -9.10836  -5.56635  -16.6328 |
| 21 | 21a  21b | DE1  DE2  DE3  DE4  DE5  DE6  DE7  DE8  DE1  DE2  DE3  DE4  DE5  DE6  DE7  DE8 | March 2003  Feb 2004  Feb 2005  May 2012  March 2013  Jan 2014  Oct 2014  June 2016  Apr 2003  Feb 2004  Feb 2005  May 2012  March 2013  Dec 2013  Nov 2014  June 2016 | Nov 2003  May 2004  Aug 2005  Sept 2012  July 2013  Apr 2014  Feb 2015  March 2017  Nov 2003  May 2004  Aug 2005  Sept 2012  July 2013  Apr 2014  Feb 2015  March 2017 | 8  3  6  4  4  3  4  9  7  3  6  4  4  4  3  9 | -8.98235  -4.30967  -8.07172  -5.67768  -5.68373  -3.65737  -5.73824  -19.1459  -8.87994  -5.40487  -6.82058  -4.81137  -5.4639  -4.9136  -4.66217  -19.1502 |
| 22 | 22a  22b | DE1  DE2  DE3  DE4  DE5  DE1  DE2  DE3  DE4  DE5 | May 2012  Nov 2012  Aug 2013  Nov 2014  Apr 2016  May 2012  Nov 2012  Aug 2013  Nov 2014  Apr 2016 | Sept 2012  June 2013  June 2014  Feb 2015  March 2017  Sept 2012  June 2013  June 2014  Feb 2015  March 2017 | 4  7  10  3  11  4  7  10  3  11 | -5.21817  -8.5063  -14.1419  -3.19702  -25.8222  -5.21817  -8.5063  -14.1419  -3.19702  -25.8222 |
| 23 | 23a  23b  23c  23d  23e  23f | DE1  DE2  DE3  DE4  DE1  DE2  DE3  DE4  DE5  DE6  DE7  DE8  DE1  DE2  DE3  DE4  DE5  DE6  DE7  DE1  DE2  DE3  DE4  DE5  DE6  DE7  DE1  DE2  DE3  DE4  DE5  DE6  DE1  DE2  DE3  DE4  DE5 | Aug 2009  Aug 2014  July 2015  May 2016  Apr 2002  May 2004  Jan 2006  Aug 2009  March 2010  Dec 2014  Oct 2015  Aug 2016  Apr 2002  May 2004  Jan 2006  March 2010  Dec 2014  Aug 2015  Aug 2016  July 2002  March 2006  Aug 2009  March 2010  Sept 2014  July 2015  Aug 2016  Apr 2002  Apr 2004  March 2006  March 2010  Aug 2015  Aug 2016  Apr 2002  Nov 2003  March 2005  Aug 2015  Aug 2016 | July 2010  March 2015  Feb 2016  June 2017  June 2003  Sept 2005  July 2006  Dec 2009  July 2010  March 2015  Jan 2016  March 2017  June 2003  Sept 2005  July 2006  July 2010  March 2015  Feb 2016  March 2017  May 2003  July 2006  Nov 2009  July 2010  Feb 2015  Feb 2016  Apr 2017  July 2003  July 2004  June 2006  July 2010  Feb 2016  March 2017  Aug 2003  Sept 2004  June 2005  Feb 2016  Feb 2017 | 11  7  7  13  14  16  6  4  4  3  3  7  14  16  6  4  3  6  7  10  4  3  4  5  7  8  15  3  3  4  6  7  16  10  3  6  6 | -12.252  -8.25148  -11.1074  -26.2762  -15.7679  -15.9402  -5.45343  -3.06462  -4.71349  -5.94919  -3.65355  -11.416  -17.741  -15.4911  -5.10622  -4.49831  -5.54597  -5.95348  -10.8666  -10.065  -3.83953  -2.56127  -5.83244  -5.09847  -9.60087  -15.4199  -17.6749  -3.07898  -2.80097  -3.94999  -10.6443  -12.9903  -25.3915  -11.3698  -3.96737  -9.47999  -7.65142 |
| 24 | 24 | DE1  DE2  DE3  DE4 | Aug 2009  July 2014  July 2015  May 2016 | July 2010  March 2015  March 2016  June 2017 | 11  8  8  13 | -9.28042  -8.06468  -11.6567  -29.1737 |
| 25 | 25 | DE1  DE2  DE3 | Apr 2002  Nov 2007  Oct 2009 | June 2004  Feb 2008  Apr 2010 | 26  3  6 | -44.2835  -2.78388  -5.91004 |
